# Diagnostic Accuracy of Symptoms, Physical Signs, and Laboratory Tests for Giant Cell Arteritis: A Systematic Review and Meta-analysis

**DOI:** 10.1001/jamainternmed.2020.3050

**Published:** 2020-08-17

**Authors:** Kornelis S. M. van der Geest, Maria Sandovici, Elisabeth Brouwer, Sarah L. Mackie

**Affiliations:** 1 Department of Rheumatology and Clinical Immunology, University of Groningen, University Medical Center Groningen, Groningen, the Netherlands; 2 Leeds Institute of Rheumatic and Musculoskeletal Medicine, NIHR (National Institute for Health Research) Leeds Biomedical Research Centre, Leeds Teaching Hospitals NHS (National Health Service) Trust, University of Leeds, Leeds, United Kingdom

## Abstract

**Importance:**

Current clinical guidelines recommend selecting diagnostic tests for giant cell arteritis (GCA) based on pretest probability that the disease is present, but how pretest probability should be estimated remains unclear.

**Objective:**

To evaluate the diagnostic accuracy of symptoms, physical signs, and laboratory tests for suspected GCA.

**Data Sources:**

PubMed, EMBASE, and the Cochrane Database of Systematic Reviews were searched from November 1940 through April 5, 2020.

**Study Selection:**

Trials and observational studies describing patients with suspected GCA, using an appropriate reference standard for GCA (temporal artery biopsy, imaging test, or clinical diagnosis), and with available data for at least 1 symptom, physical sign, or laboratory test.

**Data Extraction and Synthesis:**

Screening, full text review, quality assessment, and data extraction by 2 investigators. Diagnostic test meta-analysis used a bivariate model.

**Main Outcome(s) and Measures:**

Diagnostic accuracy parameters, including positive and negative likelihood ratios (LRs).

**Results:**

In 68 unique studies (14 037 unique patients with suspected GCA; of 7798 patients with sex reported, 5193 were women [66.6%]), findings associated with a diagnosis of GCA included limb claudication (positive LR, 6.01; 95% CI, 1.38-26.16), jaw claudication (positive LR, 4.90; 95% CI, 3.74-6.41), temporal artery thickening (positive LR, 4.70; 95% CI, 2.65-8.33), temporal artery loss of pulse (positive LR, 3.25; 95% CI, 2.49-4.23), platelet count of greater than 400 × 10^3^/μL (positive LR, 3.75; 95% CI, 2.12-6.64), temporal tenderness (positive LR, 3.14; 95% CI, 1.14-8.65), and erythrocyte sedimentation rate greater than 100 mm/h (positive LR, 3.11; 95% CI, 1.43-6.78). Findings that were associated with absence of GCA included the absence of erythrocyte sedimentation rate of greater than 40 mm/h (negative LR, 0.18; 95% CI, 0.08-0.44), absence of C-reactive protein level of 2.5 mg/dL or more (negative LR, 0.38; 95% CI, 0.25-0.59), and absence of age over 70 years (negative LR, 0.48; 95% CI, 0.27-0.86).

**Conclusions and Relevance:**

This study identifies the clinical and laboratory features that are most informative for a diagnosis of GCA, although no single feature was strong enough to confirm or refute the diagnosis if taken alone. Combinations of these symptoms might help direct further investigation, such as vascular imaging, temporal artery biopsy, or seeking evaluation for alternative diagnoses.

## Key Points

**Question:**

In patients with suspected giant cell arteritis, which clinical and laboratory findings can help to identify the disease?

**Findings:**

This systematic review and meta-analysis of 68 unique diagnostic cohort studies (14 037 unique patients) identified combinations of symptoms, physical signs, and laboratory tests that were informative with regard to the presence or absence of giant cell arteritis, but no single feature taken alone. Headache and scalp tenderness were poorly informative in this population.

**Meaning:**

These findings suggest that in patients with suspected giant cell arteritis, no single clinical or laboratory feature is sufficient to rule in or rule out the disease; therefore, additional investigations (vascular imaging and/or temporal artery biopsy) are required.

## Teaser

This systematic review and meta-analysis evaluate the diagnostic accuracy of symptoms, physical signs, and laboratory tests in patients with suspected giant cell arteritis.

## Introduction

Giant cell arteritis (GCA) is a “do-not-miss” diagnosis. Prompt diagnosis can avert visual loss.^1^ Diagnosis can be delayed in those without the classic cranial features, such as headache.^2^ Treatment for GCA consists of high-dose glucocorticoids tapered during the course of 1 year or more, but this treatment may cause substantial toxic effects, so diagnostic uncertainty must be minimized.^3^

Making a diagnosis of GCA can be challenging. The American College of Rheumatology 1990 criteria for the classification of GCA in research studies should not be used for clinical diagnosis.^4,5^ Instead, temporal artery biopsy (TAB; highly specific but with imperfect sensitivity),^6^ vascular imaging (ultrasonography, computed tomography, magnetic resonance imaging, or positron emission tomography),^7^ or a combination of these tests are recommended.^3,7^ These further investigations should be selected based on pretest probability.^3,7^ The difficulty in practice is how to quantify pretest probability given only symptoms, signs, and, if available, laboratory features. Regression, machine learning models, or clinical scoring systems have been suggested, but these rely on complete information and still require further validation.^8,9^ Pretest probability might additionally be estimated by using likelihood ratios (LRs) of clinical features to allow sequential bayesian probability revision.^10^ A previous meta-analysis^11^ reported pooled estimates of the LRs of clinical and laboratory features for a positive TAB finding. However, this previous meta-analysis included studies comparing TAB-positive vs TAB-negative GCA, which is not appropriate for estimating diagnostic accuracy. The previous meta-analysis also included diagnostic case-control studies, which often overestimate diagnostic accuracy.^12,13^ Since the earlier meta-analysis,^11^ many more relevant studies have been published.^14,15,16,17^

We conducted a systematic review and meta-analysis of the diagnostic accuracy of symptoms, physical signs, and laboratory tests for GCA. We provide summary estimates of the sensitivity, specificity, and LRs of these features. We included studies using appropriate reference standards for GCA, including TAB and clinical diagnosis. We excluded case-control studies and studies in which all patients were classified as having GCA.

## Methods

This study is reported in accordance with the 2009 Preferred Reporting Items for Systematic Reviews and Meta-analyses (PRISMA) reporting guideline.^18^ A predefined study protocol was established but not registered. No ethical approval or informed consent was required for the current systematic review and meta-analysis.

### Data Sources and Search Strategy

We searched PubMed, EMBASE, and the Cochrane Database of Systematic Reviews from December 1940 to April 5, 2020. The search strategy included terms such as *giant cell arteritis*, *temporal arteritis*, *medical history taking*, *physical examination*, *diagnostic imaging*, and *artery biopsy*. The full search strategy was developed together with an experienced medical science librarian (eTable 1 in the Supplement). We included English language records. Case reports and conference abstracts were excluded. The reference lists of included studies were screened for additional records.

### Study Selection and Eligibility Criteria

We included clinical trials and prospective or retrospective observational studies that met the following criteria: (1) participants were consecutive patients suspected of having GCA; (2) a TAB, imaging test, or clinical diagnosis was used as the reference standard for GCA; (3) a table of the true-positive, false-positive, true-negative, and false-negative counts was either directly available or could be calculated for at least 1 index test (symptom, physical sign, or laboratory test); and (4) at least 5 patients had GCA and at least 5 did not have GCA. The reference standard *clinical diagnosis* could be based on defined criteria or judgment of 1 or more physicians. We considered *healed temporal arteritis* (ie, intimal hyperplasia and/or internal elastic lamina disruption in the absence of an arterial inflammatory infiltrate) as a negative TAB result, because it might indicate atherosclerosis rather than GCA.^6^ We excluded studies in which all patients were diagnosed with GCA and/or the closely related disease polymyalgia rheumatica.^19^ We excluded case-control studies. Titles and abstracts were screened by 2 independent reviewers (K.S.M.vdG. and M.S.). Full texts were independently assessed in Covidence by 2 reviewers (K.S.M.vdG. and M.S. or S.L.M.). Disagreement between reviewers was resolved by consensus or, if consensus could not be obtained, by consulting a third reviewer (E.B.) who made the final decision.

### Data Collection

Study characteristics and data from 2 × 2 tables were extracted by 1 reviewer (K.S.M.vdG.) and checked by a second reviewer (E.B. or S.L.M.). A standardized data sheet was used to collect information on study characteristics (eAppendix in the Supplement). We extracted any clinical or laboratory finding reported, as well as data on age and sex. Composite findings (eg, symptom A plus symptom B) were not recorded. Authors of studies were not contacted. If potential data overlap existed among studies from the same hospital, data were obtained from the largest study. When multiple reference standards were available in 1 study, the clinical diagnosis was used as the reference standard for the main study analysis. A C-reactive protein (CRP) level of less than 0.5 mg/dL (to convert to mg/L, multiply by 10) was considered the reference value unless other laboratory-specific reference values were reported. Disagreement between reviewers was either resolved by consensus or, if consensus could not be obtained, by consulting a third reviewer (E.B. or S.L.M.), who made the final decision.

### Quality Assessment

The risk of bias was evaluated by 2 reviewers (K.S.M.vdG. and E.B.) with the quality assessment of diagnostic accuracy studies (QUADAS-2) tool (eAppendix in the Supplement). The QUADAS-2 tool focuses on the bias and applicability of study results regarding patient selection, the index test, the reference standard, and study flow and timing.^20^

### Synthesis of Results

Study heterogeneity was evaluated by plotting the estimates of sensitivity and specificity in forest plots and receiver operating characteristics (ROC) space. We used hierarchical logistic regression modeling to determine summary estimates of the sensitivity, specificity, diagnostic odds ratio, and LRs by the bivariate model approach, as well as hierarchical summary ROC (HSROC) plots.^21^ Likelihood ratios of greater than 2.00 or less than 0.50 with 95% CIs not including 1.00 were considered statistically significant.^10,22^ We performed the following sensitivity analyses: (1) a predefined comparison of LRs in studies using distinct reference standards for GCA; (2) a nonpredefined comparison of LRs in prospective and retrospective studies; and (3) a predefined analysis restricted to pretreatment laboratory tests. Our primary analysis and sensitivity analyses included any index test reported by 4 or more studies. Hierarchical logistic regression modeling analysis and evaluation of funnel plot asymmetry were performed in STATA, version 15.1 (StataCorp LLC) with the metandi, metandiplot, and midas commands.^23^ Forest plots were created in Review Manager, version 5.3 (Cochrane) and StatsDirect, version 3.2.10 (StatsDirect Ltd).

## Results

### Study Characteristics

Of the 1436 reports screened, 68 studies^14,15,16,17,24,25,26,27,28,29,30,31,32,33,34,35,36,37,38,39,40,41,42,43,44,45,46,47,48,49,50,51,52,53,54,55,56,57,58,59,60,61,62,63,64,65,66,67,68,69,70,71,72,73,74,75,76,77,78,79,80,81,82,83,84,85,86,87^ fulfilled the selection criteria and were used for the systematic review and meta-analysis (eFigure 1 in the Supplement). These studies included 14 037 patients, of whom 4277 (30.5%) were classified as having GCA (Table 1 and eTable 2 in the Supplement). Most reports were retrospective cohort studies (48 [70.6%])^14,15,27,29,31,32,34,35,36,37,38,39,40,41,43,44,45,46,48,49,50,51,53,54,58,59,62,64,65,66,67,68,70,71,72,73,74,75,77,78,79,80,81,83,84,85,86,87^ and performed at academic centers (56 [82.4%]).^16,17,24,25,26,27,28,29,30,31,32,33,34,35,37,38,40,41,42,43,44,45,46,47,48,49,50,51,52,53,54,55,56,57,59,60,61,62,66,67,68,69,72,73,75,76,77,78,79,80,81,82,83,84,86,87^ TAB was the reference standard in 38 studies (55.9%).^14,15,16,26,30,32,37,38,39,40,41,42,43,44,45,48,49,52,56,58,59,60,62,64,65,67,70,72,73,78,79,80,81,83,84,85,86,87^ The mean or the median length of the TAB specimen was generally greater than 1 cm. A variable proportion of patients underwent bilateral TAB (eTable 3 in the Supplement). In 30 studies (44.1%),^17,24,25,27,28,29,31,33,34,35,36,46,47,50,51,53,54,55,57,61,63,66,68,69,71,74,75,76,77,82^ clinical diagnosis was the reference standard for GCA; in 8 of these studies,^31,46,53,71,75,76,77,82^ all patients underwent TAB, and in 9 studies,^17,33,47,51,61,63,68,69,74^ patients had a combination of TAB and imaging (eTable 4 in the Supplement). The clinical diagnosis was typically based on clinical and laboratory findings, imaging and/or TAB results, and a good initial response to glucocorticoid treatment (eTable 5 in the Supplement). In 16 of the studies using clinical diagnosis as reference standard,^17,29,31,33,34,36,47,51,53,54,57,61,68,69,76,82^ patients were all followed up to verify that the clinical diagnosis was not later revised. Only 1 study^68^ allowed us to evaluate imaging as the reference standard in addition to the clinical diagnosis and TAB.

**Table 1.** Characteristics of the 68 Included Studies

Characteristic	No. (%)^a^	
	Studies (n = 68)	Patients (n = 14 037)^b^
Year of publication		
Before 1990	9 (13.2)	797 (5.7)
1990-1999	6 (8.8)	1235 (8.8)
2000-2009	13 (19.1)	2119 (15.1)
2010-2019	40 (58.8)	9886 (70.4)
Study design		
Prospective cohort	20 (29.4)	2104 (15.0)
Retrospective cohort	48 (70.6)	11 933 (85.0)
Setting of care		
Nonacademic center	7 (10.3)	664 (4.7)
Academic center	56 (82.4)	7777 (55.4)
Nonacademic/academic center	4 (5.9)	3155 (22.5)
Unclear	1 (1.5)	2441 (17.4)
Identification of patients		
Central pathology/surgery registry	28 (41.2)	10 337 (73.6)
Central imaging registry	7 (10.3)	412 (2.9)
Central pathology/surgery and central imaging registry	2 (2.9)	55 (0.4)
Ophthalmology department	14 (20.6)	1452 (10.3)
Rheumatology department	7 (10.3)	709 (5.1)
Multiple hospital departments	9 (13.2)	1006 (7.2)
Unclear	1 (1.5)	66 (0.5)
Specialty referring patients		
Primary care	1 (1.5)	125 (0.9)
Hospital departments	3 (4.4)	481 (3.4)
Primary care and hospital departments	8 (11.8)	2701 (19.2)
Unclear	56 (82.4)	10 730 (76.4)
Laboratory results before treatment		
No	8 (11.8)	2779 (19.8)
Yes	6 (8.8)	800 (5.7)
Unclear	27 (39.7)	5824 (41.5)
Not applicable	27 (39.7)	4634 (33.0)
Type of reference standard		
TAB	38 (55.9)	11 207 (79.8)
Clinical diagnosis^c^	30 (44.1)	2830 (20.2)
Focus of diagnostic testing		
Cranial arteries	53 (77.9)	12 543 (89.4)
Systemic arteries	1 (1.5)	63 (0.4)
Cranial and systemic arteries	14 (20.6)	1431 (10.2)

Abbreviations: GCA, giant cell arteritis; TAB, temporal artery biopsy.

^a^ Percentages have been rounded and may not total 100.

^b^ A total of 4277 patients were classified as having GCA.

^c^ Seven studies with the clinical diagnosis as the reference standard^46,68,71,75,76,77,82^ also allowed evaluation of TAB as the reference standard (558 patients). One study with the clinical diagnosis as the reference standard^68^ also allowed evaluation of ultrasonography as the reference standard (23 patients).

### Evaluation of Bias

Patient selection was the principal source of bias (eFigures 2 and 3 in the Supplement). Studies using TAB as the reference standard may have been more prone to selection bias because a sufficient index of clinical suspicion is required to order this invasive test. Conversely, studies using the clinical diagnosis as the reference standard were at high risk of bias because the index test result contributed to the clinical diagnostic decision.

### Diagnostic Value of Symptoms and Demographic Features

In studies reporting the sex of patients (n = 7798), 2605 (33.4%) of patients were male and 5193 (66.6%) were female. Although headache is considered to be a key symptom for GCA, the positive and negative LRs for headache did not meet our prespecified threshold for statistical significance (Table 2). Double vision provided a positive LR of 1.72 (95% CI, 1.12-2.63). Positive LRs of more than 2.00 were found for limb claudication (6.01; 95% CI, 1.38-26.16), jaw claudication (4.90; 95% CI, 3.74-6.41), and a previous diagnosis of polymyalgia rheumatica (2.07; 95% CI, 0.92-4.65), whereas being older than 70 years had a negative LR of less than 0.50 (0.48; 95% CI, 0.27-0.86). The forest plots and HSROC curves indicated substantial heterogeneity for all statistically significant index tests except for jaw claudication (eFigures 4 and 5 in the Supplement). Overall, we found little evidence of publication bias by evaluation of funnel plot asymmetry (eFigure 6 in the Supplement). Symptoms reported by less than 4 studies are shown in eTable 6 in the Supplement.

**Table 2.** Diagnostic Accuracy of Demographics and Symptoms

Finding by study	No. of patients (No. of cohorts)	Sensitivity (95% CI), %	Specificity (95% CI), %	Diagnostic OR (95% CI)	Positive LR (95% CI)	Negative LR (95% CI)
**Demographics**						
Age, y						
>60^a^	261 (7)	96.6 (76.0-99.6)	22.6 (15.4-31.8)	8.39 (1.05-67.11)	1.25 (1.12-1.39)	0.15 (0.02-1.13)
>70^a^	261 (7)	73.5 (49.5-88.7)	55.3 (39.2-70.3)	3.42 (1.68-6.96)	1.64 (1.29-2.09)	0.48 (0.27-0.86)^x^
>80^b^	208 (6)	19.0 (10.4-32.0)	85.1 (73.4-92.1)	1.33 (0.62-2.86)	1.27 (0.67-2.40)	0.95 (0,84-1.09)
Male^c^	7798 (42)	31.7 (29.6-33.9)	64.9 (62.5-67.2)	0.86 (0.77-0.96)	0.90 (0.84-0.97)	1.05 (1.01-1.09)
**Symptoms**						
Cranial						
Headache^d^	6918 (36)	72.2 (68.3-75.8)	45.7 (39.1-52.4)	2.19 (1.72-2.78)	1.33 (1.19-1.48)	0.61 (0.53-0.70)
Temporal headache^e^	545 (4)	65.9 (37.4-86.2)	31.8 (14.1-57.1)	0.90 (0.56-1.46)	0.97 (0.82-1.14)	1.07 (0.78-1.47)
Scalp tenderness^f^	2951 (15)	38.9 (31.7-46.7)	78.9 (69.7-85.9)	2.39 (1.70-3.34)	1.85 (1.40-2.44)	0.77 (0.71-0.84)
Jaw claudication^g^	6867 (35)	37.5 (33.8-41.3)	92.3 (89.6-94.4)	7.24 (5.45-9.62)	4.90 (3.74-6.41)^x^	0.68 (0.64-0.71)
Visual disturbance^h^	3023 (25)	33.9 (29.6-38.4)	71.8 (66.7-76.4)	1.30 (1.06-1.60)	1.20 (1.04-1.39)	0.92 (0.86-0.98)
Loss of vision^i^	4585 (14)	21.7 (15.1-30.3)	85.3 (76.2-91.3)	1.61 (1.21-2.14)	1.48 (1.15-1.91)	0.92 (0.88-0.96)
Transient loss of vision^j^	1181 (9)	10.7 (7.1-16.0)	92.9 (86.6-96.4)	1.57 (0.88-2.82)	1.51 (0.88-2.60)	0.96 (0.92-1.01)
Double vision^k^	3799 (8)	6.5 (4.5-9.3)	96.2 (93.2-97.9)	1.76 (1.13-2.75)	1.72 (1.12-2.63)	0.97 (0.95-0.99)
Cerebrovascular accident^l^	1089 (5)	2.6 (1.3-5.1)	95.9 (89.0-98.5)	0.62 (0.23-1.63)	0.63 (0.25-1.59)	1.02 (0.98-1.06)
Systemic						
Constitutional symptoms^m^	1274 (8)	62.5 (35.5-83.5)	46.8 (29.1-65.2)	1.47 (0.89-2.41)	1.17 (1.00-1.38)	0.80 (0.56-1.14)
Malaise^n^	1267 (10)	55.5 (44.0-66.4)	51.7 (38.8-64.4)	1.33 (1.02-1.75)	1.15 (1.00-1.33)	0.86 (0.75-0.99)
Anorexia^o^	1932 (8)	40.2 (28.0-53.8)	74.5 (64.5-82.5)	1.97 (1.51-2.57)	1.58 (1.33-1.88)	0.80 (0.71-0.91)
Weight loss^p^	2882 (18)	39.3 (31.0-48.3)	76.7 (72.2-80.6)	2.13 (1.64-2.77)	1.69 (1.44-1.98)	0.79 (0.71-0.89)
Fever^q^	3091 (23)	26.7 (19.8-34.9)	78.0 (68.4-85.3)	1.29 (1.03-1.62)	1.21 (1.01-1.46)	0.94 (0.90-0.99)
Other						
Myalgia^r^	1855 (15)	39.8 (35.0-44.9)	57.5 (46.9-67.4)	0.90 (0.61-1.31)	0.94 (0.75-1.17)	1.05 (0.89-1.23)
PMR^s^	2814 (23)	33.4 (27.5-39.8)	74.3 (65.9-81.2)	1.45 (1.14-1.84)	1.30 (1.08-1.56)	0.90 (0.84-0.95)
Previous PMR^t^	519 (4)	19.1 (13.4-26.5)	90.8 (82.3-95.4)	2.32 (0.92-5.82)	2.07 (0.92-4.65)	0.89 (0.79-1.00)
Arthralgia^u^	656 (6)	25.4 (15.5-38.6)	73.3 (64.6-80.6)	0.94 (0.60-1.46)	0.95 (0.68-1.33)	1.02 (0.91-1.14)
Limb claudication^v^^,^^w^	405 (6)	19.6 (12.5-29.4)	96.7 (84.2-99.4)	7.23 (1.62-32.21)	6.01 (1.38-26.16)^x^	0.83 (0.76-0.91)

Abbreviations: LR, likelihood ratio; OR, odds ratio; PMR, polymyalgia rheumatica.

^a^ From 7 of the analyzed studies.^28,30,44,50,53,71,83^

^b^ From 6 of the analyzed studies.^28,30,44,50,53,71^

^c^ From 43 of the analyzed studies.^14,15,17,26,27,29,30,31,32,33,34,35,36,37,43,44,45,47,49,50,51,52,54,55,57,61,62,64,65,69,70,71,73,74,75,76,77,80,81,82,84,87^

^d^ From 36 of the analyzed studies.^15,17,29,32,35,36,37,39,43,45,46,47,48,49,52,54,55,57,58,61,64,65,66,69,71,72,73,74,75,76,77,80,82,83,84,87^

^e^ From 4 of the analyzed studies.^16,27,33,34^

^f^ From 15 of the analyzed studies.^16,17,27,33,39,49,52,54,55,60,61,66,69,73,87^

^g^ From 35 of the analyzed studies.^15,17,27,32,33,34,35,36,37,39,43,45,46,47,48,52,53,54,55,57,58,61,64,66,69,71,72,73,75,76,77,80,82,84,87^

^h^ From 25 of the analyzed studies.^24,25,27,29,32,34,35,39,41,46,47,54,58,61,63,64,65,66,69,71,73,76,80,82,84^

^i^ From 14 of the analyzed studies.^15,39,43,45,49,55,57,63,69,72,74,75,77,87^

^j^ From 9 of the analyzed studies.^16,17,39,43,55,67,69,79,86^

^k^ From 8 of the analyzed studies.^15,17,39,43,49,69,77,87^

^l^ From 5 of the analyzed studies.^43,46,61,63,73^

^m^ From 8 of the analyzed studies.^16,33,37,46,51,61,64,69^

^n^ From 10 of the analyzed studies.^16,17,36,52,58,71,73,80,82,84^

^o^ From 8 of the analyzed studies.^16,17,39,58,73,80,84,87^

^p^ From 18 of the analyzed studies.^16,24,25,34,36,39,43,54,58,61,69,71,73,79,80,82,84,87^

^q^ From 23 of the analyzed studies.^16,32,35,36,39,41,43,47,48,49,52,54,55,57,58,61,65,69,71,73,75,80,84^

^r^ From 15 of the analyzed studies.^16,35,36,43,47,52,55,57,58,61,69,73,75,80,84^

^s^ From 23 of the analyzed studies.^17,24,25,32,33,34,37,39,41,46,48,49,53,58,60,64,65,66,71,75,76,82,86^

^t^ From 4 of the analyzed studies.^16,32,54,79^

^u^ From 6 of the analyzed studies.^16,39,58,75,80,84^

^v^ From 6 of the analyzed studies.^29,35,51,69,71,82^

^w^ Limb claudication was restricted to the arms in one study^71^ and to the legs in another study.^29^

^x^ Statistically significant due to summary estimate of the positive LR of greater than 2.00 or the negative LR of less than 0.50 and a 95% CI not including 1.00.

### Diagnostic Value of Physical Signs and Laboratory Tests

A positive LR of more than 2.00 occurred for findings related to temporal artery thickening (LR, 4.70; 95% CI, 2.65-8.33), temporal artery loss of pulse (3.25; 95% CI, 2.49-4.23), temporal tenderness (3.14; 95% CI, 1.14-8.65), an abnormal temporal artery (2.29; 95% CI, 1.61-3.26), anterior ischemic optic neuropathy (2.15; 95% CI, 1.53-3.03), erythrocyte sedimentation rate (ESR) of greater than 60 (2.40; 95% CI, 1.71-3.36), 80 (2.79; 95% CI, 1.78-4.37), and 100 mm/h (3.11; 95% CI, 1.43-6.78), and a platelet count of greater than 400 × 10^3^/μL all (to convert to ×10^9^/L, multiply by 1) (3.75; 95% CI, 2.12-6.64) (Table 3). Negative LRs of less than 0.50 occurred for an ESR of more than 40 mm/h (0.18; 95% CI, 0.08-0.44), more than 50 mm/h (0.48; 95% CI, 0.38-0.62), and more than 60 mm/h (0.42; 95% CI, 0.28-0.61), CRP level of at least 2.5 mg/dL (0.38; 95% CI, 0.25-0.59), or a CRP level of greater than the reference value (0.40; 95% CI, 0.29-0.56). Overall, moderate heterogeneity and little funnel plot asymmetry was observed (eFigures 4, 5, and 6 in the Supplement). Physical findings reported by fewer than 4 studies are shown in eTable 7 in the Supplement.

**Table 3.** Diagnostic Accuracy of Physical and Laboratory Findings

Finding by study	No. of patients (No. of cohorts)	Sensitivity (95% CI), %	Specificity (95% CI), %	Diagnostic OR (95% CI)	Positive LR (95% CI)	Negative LR (95% CI)
Physical and fundoscopic abnormalities						
Any temporal artery abnormality^a^^,^^b^	3823 (13)	52.9 (39.3-66.0)	76.9 (64.6-85.9)	3.73 (2.30-6.06)	2.29 (1.61-3.26)^c^	0.61 (0.49-0.77)
Temporal tenderness^d^	658 (5)	51.4 (37.6-65.1)	83.6 (59.1-94.5)	5.41 (1.58-18.46)	3.14 (1.14-8.65)^c^	0.58 (0.43-0.79)
Temporal artery thickening^e^	929 (8)	44.4 (31.3-58.2)	90.6 (81.8-95.4)	7.65 (4.04-14.48)	4.70 (2.65-8.33)^c^	0.61 (0.50-0.76)
Temporal artery loss of pulse^f^	1227 (7)	38.2 (31.3-45.5)	88.2 (85.6-90.4)	4.63 (3.22-6.67)	3.25 (2.49-4.23)^c^	0.70 (0.62-0.79)
Temporal artery tenderness^g^	1136 (10)	36.0 (22.1-52.6)	81.4 (66.5-90.6)	2.46 (1.43-4.22)	1.93 (1.25-2.99)	0.79 (0.66-0.93)
AION^h^	1181 (7)	23.9 (13.0-40.0)	88.9 (80.8-93.8)	2.51 (1.63-3.87)	2.15 (1.53-3.03)^c^	0.86 (0.75-0.97)
Ischemic optic neuropathy^i^	682 (4)	21.9 (10.9-39.3)	87.3 (75.5-93.9)	1.94 (0.83-4.51)	1.73 (0.86-3.49)	0.89 (0.76-1.05)
CRAO^j^	647 (5)	6.5 (3.1-12.9)	95.6 (85.6-98.7)	1.53 (0.48-4.89)	1.49 (0.49-4.53)	0.98 (0.93-1.03)
Laboratory findings						
Anemia^k^	2725 (14)	54.5 (41.2-67.2)	55.3 (42.4-67.6)	1.48 (1.22-1.79)	1.22 (1.10-1.36)	0.82 (0.74-0.92)
CRP level elevated^l^^,^^m^	1849 (9)	87.4 (80.4-92.1)	31.4 (25.4-38.0)	3.16 (2.21-4.53)	1.27 (1.20-1.35)	0.40 (0.29-0.56)^c^
CRP level ≥2.5 mg/dL^n^	1121 (5)	79.2 (63.5-89.3)	54.2 (40.1-67.7)	4.50 (2.84-7.14)	1.73 (1.41-2.12)	0.38 (0.25-0.59)^c^
ESR elevated^o^^,^^p^	3429 (15)	82.6 (74.4-88.6)	33.8 (25.6-43.1)	2.43 (1.62-3.65)	1.25 (1.12-1.39)	0.51 (0.37-0.71)
ESR>40 mm/h^q^	546 (9)	93.2 (79.7-97.9)	37.5 (21.1-57.4)	8.17 (3.40-19.62)	1.49 (1.16-1.92)	0.18 (0.08-0.44)^c^
ESR>50 mm/h^r^^,^^s^	1966 (18)	78.9 (71.7-84.7)	43.5 (34.1-53.4)	2.88 (2.05-4.05)	1.40 (1.22-1.60)	0.48 (0.38-0.62)^c^
ESR>60 mm/h^t^	270 (6)	70.7 (56.2-81.9)	70.5 (57.5-80.9)	5.77 (3.26-10.23)	2.40 (1.71-3.36)^c^	0.42 (0.28-0.61)^c^
ESR>80 mm/h^t^	270 (6)	50.7 (31.2-69.9)	81.8 (74.4-87.4)	4.62 (2.07-10.29)	2.79 (1.78-4.37)^c^	0.60 (0.41-0.90)
ESR>100 mm/h^u^	368 (7)	24.2 (13.0-40.6)	92.2 (81.1-97.1)	3.79 (1.60-8.97)	3.11 (1.43-6.78)^c^	0.82 (0.70-0.96)
Platelet count >400 × 10^3^/μL^v^^,^^w^	2316 (5)	45.8 (33.0-59.3)	87.8 (81.1-92.3)	6.08 (2.74-13.49)	3.75 (2.12-6.64)^c^	0.62 (0.47-0.80)

Abbreviations: AION, anterior ischemic optic neuropathy; CRAO, central retinal artery occlusion; CRP, C-reactive protein; ESR, erythrocyte sedimentation rate; LR, likelihood ratio; OR, odds ratio.

SI conversion factors: To convert CRP to mg/L, multiply by 10; platelet count to ×10^9^/L, multiply by 1.

^a^ A precise definition was typically lacking; it was for instance reported as abnormal temporal artery or temporal artery abnormality.

^b^ From 13 of the analyzed studies.^15,33,41,42,46,49,52,54,55,64,71,80,84^

^c^ Statistically significant due to summary estimate of the positive LR of greater than 2.00 or the negative LR of less than 0.50 and a 95% CI not including 1.00.

^d^ From 5 of the analyzed studies.^34,36,43,65,77^

^e^ From 8 of the analyzed studies.^24,25,34,35,63,71,73,82^

^f^ From 7 of the analyzed studies.^16,43,47,63,71,72,73^

^g^ From 10 of the analyzed studies.^16,47,53,57,58,63,71,73,75,83^

^h^ From 7 of the analyzed studies.^43,48,56,60,63,67,86^

^i^ From 4 of the analyzed studies.^37,63,75,79^

^j^ From 5 of the analyzed studies.^43,56,67,79,86^

^k^ From 14 of the analyzed studies.^30,32,35,39,46,50,52,64,71,73,80,82,84,87^

^l^ Defined as at least 0.5 mg/dL unless other laboratory-specific reference values were reported.

^m^ From 9 of the analyzed studies.^14,28,37,59,61,63,64,66,68^

^n^ From 5 of the analyzed studies.^28,54,68,70,85^

^o^ Defined as greater than 20 mm/h in men and greater than 30 mm/h in women, unless other laboratory-specific reference values were reported. In 8 of 32 (25%) studies reporting the ESR, it was clearly stated the Westergren method was used.

^p^ From 15 of the analyzed studies.^14,16,26,28,30,37,40,44,47,48,57,59,66,78,87^

^q^ From 9 of the analyzed studies.^28,30,38,44,53,65,77,80,84^

^r^ Includes studies reporting an ESR of at least 50 mm/h.

^s^ From 18 of the analyzed studies.^26,28,30,31,32,37,38,44,54,62,64,65,70,71,73,81,82,83^

^t^ From 6 of the analyzed studies.^28,30,38,44,53,65^

^u^ From 7 of the analyzed studies.^28,30,32,38,44,53,65^

^v^ Includes studies reporting a platelet count of at least 400 × 10^3^/μL.

^w^ From 5 of the analyzed studies.^26,37,40,70,85^

### Sensitivity Analyses

Results of our sensitivity analyses are provided in eTables 8 to 10 in the Supplement. We found comparable LRs in our comparison of studies with different reference standards (TAB vs clinical diagnosis) or study design (prospective vs retrospective). A pretreatment elevated CRP level showed a sensitivity of 90.1% (95% CI, 76.3%-96.3%) and a negative LR of 0.38 (95% CI, 0.17-0.81) for a diagnosis of GCA. A pretreatment ESR of greater than 50 mm/h had a sensitivity of 87.5% (95% CI, 78.3%-93.1%) and negative LR of 0.27 (95% CI, 0.13-0.57).

## Discussion

### Main Findings

This updated meta-analysis provides more precise estimates of LRs associated with symptoms, signs, and laboratory features of GCA. Features that, if present, should upgrade the level of suspicion for GCA are limb claudication; jaw claudication; various temporal artery abnormalities; a platelet count of greater than 400 × 10^3^/μL; ESRs of greater than 60, 80, and 100 mm/h; and anterior ischemic optic neuropathy. Features that should downgrade the level of suspicion for GCA are 70 years or younger; a CRP level in the reference range or less than 2.5 mg/dL; and an ESR of no greater than 40, 50, or 60 mm/h. For most patients with suspected GCA, no single feature is likely to shift pretest probability sufficiently to render further investigation for GCA unnecessary. However, these likelihood ratios may inform clinical decisions, including selection and timing of investigations, and whether to immediately commence high-dose glucocorticoid therapy or await further test results.^88,89^

### Association With Other Studies

Our findings confirm and extend those of the previous meta-analysis,^11^ which had included 21 studies of 2680 patients. We were able to show that an elevated ESR, especially greater than 60 mm/h, is informative in suggesting a diagnosis of GCA. We improved the precision and clinical utility of the summary estimates. For example, the previous meta-analysis^11^ estimated the positive LR for double vision as 3.4 (95% CI, 1.3-8.6); with greater patient numbers, we estimate the positive LR as 1.72 (95% CI, 1.12-2.63). We were also able to evaluate the diagnostic accuracy of further features, including transient loss of vision, cerebrovascular accident, limb claudication, central retinal artery occlusion, CRP levels, and platelet counts. Furthermore, we conducted sensitivity analyses to evaluate for bias arising from choice of reference standard, prospective vs retrospective studies, and whether all laboratory tests were explicitly stated as occurring before treatment.

Various tools have been developed that could help to estimate GCA probability. These tools require assessment of a limited set of clinical and laboratory features that were originally selected by expert opinion and then weighted based on expert opinion or statistical methods.^8,9^ Interestingly, both tools contain features, such as sex, that were not very helpful in changing GCA probability according to our meta-analysis. Some clinical features in these tools, such as symptom duration and alternative diagnosis,^8^ could not be included in our meta-analysis owing to lack of published data.

Our meta-analysis indicates that some features considered classic for GCA, such as headache, scalp tenderness, and constitutional symptoms, have limited use for upgrading or downgrading the clinical probability of GCA. This does not mean, however, that these symptoms are irrelevant. Our meta-analysis shows that the prevalence of these classic features is high among patients with and without GCA, suggesting that the diagnostic value of these symptoms may have been used up earlier in the care pathway.^90^ Headache is important in prompting suspicion of GCA and onward referral to a specialist, but once that referral decision has been made, clinicians should be cautious about overvaluing the diagnostic significance of headache and should evaluate patients for the other features identified in our meta-analysis as informative for a final diagnosis of GCA.

### Limitations

Our study was limited by the quality of the studies included. Although we performed a comprehensive search for published studies, we cannot exclude that relevant data was omitted owing to exclusion of non-English articles and conference abstracts. No unpublished data were obtained via contact with authors.

Several sources of bias were present in our meta-analysis. First, studies using TAB may have been at risk of selection bias because the decision for TAB necessarily depends on the presence of clinical and laboratory features to justify this invasive test. Second, clinical diagnosis is subjective and relies on clinical and laboratory features as well as further tests; this circularity could lead to overestimation of the diagnostic accuracy of index tests. Third, many studies were retrospective cohort studies, which could have introduced further selection bias. We mitigated these risks of bias by performing sensitivity analyses, which did not show substantial differences between studies with distinct reference standards or between studies with retrospectively and prospectively gathered data.

The reference standards for GCA may have additional limitations. Although the sensitivity of TAB may be 77% for fulfilment of the American College of Rheumatology 1990 criteria for GCA,^91^ it is likely lower for the clinical diagnosis in daily clinical practice.^6^ Some studies in our meta-analysis^46,68,71,75,76,77,82^ reported a subgroup of patients with TAB findings that were negative for GCA. Patients with GCA may have had TAB findings negative for GCA in other studies, but these patients were simply classified as not having GCA. Thus, the diagnostic accuracy of clinical and laboratory features might have been underestimated. The clinical diagnosis of GCA might be subjective and strongly related to the experience of the individual physician making the diagnosis. The clinical diagnosis was only ascertained by follow-up in a minority of studies. Nevertheless, we observed comparable LRs of clinical and laboratory features in studies using TAB or the clinical diagnosis as the reference standard.

A clear definition of symptoms was lacking in the studies included in our meta-analysis. This might be relevant for a symptom such as jaw claudication. Jaw claudication typically occurs after 2 to 3 minutes of chewing,^92^ but temporomandibular joint pain is common in older people and also causes pain with chewing. Lack of a clear definition of jaw claudication might possibly inflate the LR of this clinical feature, because it allows clinicians to classify aching on chewing as either jaw claudication or temporomandibular joint pain based on the clinical judgment that GCA is likely or not. Because jaw claudication is not described in any other disease and might be considered almost pathognomonic of GCA, clinicians may be reluctant to document jaw claudication unless they are fairly sure for other reasons that the patient has GCA.

Glucocorticoid treatment may be commenced immediately when GCA is suspected. This treatment could have affected index test results, particularly the laboratory tests. It was surprising that only few reports explicitly stated that the laboratory test results were obtained before treatment. Our sensitivity analysis for pretreatment laboratory measures could only be performed for an elevated CRP level and an ESR of greater than 50 mm/h. These pretreatment laboratory features tended to show better sensitivity and negative LRs than those obtained in the main study analysis.

The meta-analysis method we used required us to dichotomize continuous variables associated with GCA (age and laboratory values), which is inefficient and likely results in underestimation of diagnostic utility. However, individual patient data meta-analysis would have been needed to overcome this.

Study heterogeneity was observed for various clinical and laboratory features with relevant LRs. Additional prospective studies are needed to confirm the summary estimates of these features. We therefore recommend that complete sets of clinical and pretreatment laboratory data are reported in diagnostic cohort studies, either in summary tables or as raw data. This process would allow investigators to determine summary estimates of diagnostic accuracy parameters with more precision. Prospective studies would ideally consist of all patients who have been evaluated for GCA by every specialty or department in a hospital.^90^

## Conclusions

This systematic review and meta-analysis highlight the clinical and laboratory features that may be informative in making a diagnosis of GCA and that should be assessed when evaluating patients with suspected GCA. They should also be reported in future diagnostic cohort studies. Clinicians should obtain a comprehensive history, physical examination, and laboratory evaluation for each patient suspected of having GCA. No single symptom, physical sign, or laboratory test is sufficient to completely rule in or rule out GCA. An additional imaging test or TAB is typically needed to make a confident diagnosis of GCA. Our study could not determine whether individual LRs can be combined, or whether there is collinearity between particular features (eg, ESRs and CRP levels with constitutional symptoms). Nonetheless, this study provides important data that could inform a future bayesian probability revision approach to investigation, diagnosis, and management of suspected GCA, which would need to be prospectively validated in future studies.

## Notes

Supplement. **eTable 1.** Search Strategies **eTable 2.** Details of Included Studies **eTable 3.** Studies With TAB as Reference Standard **eTable 4.** Studies With Clinical Diagnosis as Reference Standard **eTable 5.** Study-Specific Definitions of Clinical Diagnosis **eTable 6.** Symptoms With Limited Data Available **eTable 7.** Physical Findings With Limited Data Available **eTable 8.** Sensitivity Analysis: Distinct Reference Standards **eTable 9.** Sensitivity Analysis: Prospective vs Retrospective Studies **eTable 10.** Sensitivity Analysis: Pretreatment Laboratory Tests **eAppendix.** Standard Form for Study Characteristics and QUADAS-2 Items **eFigure 1.** PRISMA Flow Diagram **eFigure 2.** Overall Summary of QUADAS-2 Items **eFigure 3.** Detailed Summary of QUADAS-2 Items **eFigure 4.** Forests Plots **eFigure 5.** HSROC Curves **eFigure 6.** Funnel Plots **eReferences.**
