# Supplementary material for: Diagnostic Accuracy of Symptoms, Physical Signs, and Laboratory Tests for Giant Cell Arteritis: A Systematic Review and Meta-analysis
Source: JAMA Intern Med. 2020 Aug 17;180(10):1295–304. doi: 10.1001/jamainternmed.2020.3050 (PMC7432275; doi:10.1001/jamainternmed.2020.3050)
Supplement: Supplement. — eTable 1. Search Strategies eTable 2. Details of Included Studies eTable 3. Studies With TAB as Reference Standard eTable 4. Studies With Clinical Diagnosis as Reference Standard eTable 5. Study-Specific Definitions of Clinical Diagnosis eTable 6. Symptoms With Limited Data Available eTable 7. Physical Findings With Limited Data Available eTable 8. Sensitivity Analysis: Distinct Reference Standards eTable 9. Sensitivity Analysis: Prospective vs Retrospective Studies eTable 10. Sensitivity Analysis: Pretreatment Laboratory Tests eAppendix. Standard Form for Study Characteristics and QUADAS-2 Items eFigure 1. PRISMA Flow Diagram eFigure 2. Overall Summary of QUADAS-2 Items eFigure 3. Detailed Summary of QUADAS-2 Items eFigure 4. Forests Plots eFigure 5. HSROC Curves eFigure 6. Funnel Plots eReferences. [file jamainternmed-e203050-s001.pdf]

Supplementary Online Content

Van der Geest KSM, Sandovici M, Brouwer E, Mackie SL. Diagnostic accuracy of symptoms, physical signs, and laboratory tests for giant cell arteritis: a systematic review and meta-analysis. Published online August 17, 2020. *JAMA Intern Med*. doi:10.1001/jamainternmed.2020.3050

eTable 1. Search Strategies

eTable 2. Details of Included Studies

eTable 3. Studies With TAB as Reference Standard

eTable 4. Studies With Clinical Diagnosis as Reference Standard

eTable 5. Study-Specific Definitions of Clinical Diagnosis

eTable 6. Symptoms With Limited Data Available

eTable 7. Physical Findings With Limited Data Available

eTable 8. Sensitivity Analysis: Distinct Reference Standards

eTable 9. Sensitivity Analysis: Prospective vs Retrospective Studies

eTable 10. Sensitivity Analysis: Pretreatment Laboratory Tests

eAppendix. Standard Form for Study Characteristics and QUADAS-2 Items

eFigure 1. PRISMA Flow Diagram

eFigure 2. Overall Summary of QUADAS-2 Items

eFigure 3. Detailed Summary of QUADAS-2 Items

eFigure 4. Forests Plots

eFigure 5. HSROC Curves

eFigure 6. Funnel Plots

eReferences.

This supplementary material has been provided by the authors to give readers additional information about their work.

©2020 American Medical Association. All rights reserved.

eTable 1. Search Strategies

A. Search Strategy in Pubmed	
Date	April 5, 2020
Search strategy	("Giant Cell Arteritis"[Mesh] OR Giant Cell Arteritis [tiab] OR temporal arteritis [tiab] OR hortons disease [tiab] OR horton's disease [tiab])<br>AND<br>("Physical Examination"[Mesh] OR physical exam* [tiab] OR "Medical History Taking"[Mesh] OR "Sensitivity and Specificity"[Mesh] OR sensitiv* [tiab] OR specificit* [tiab] OR accura* [tiab] OR patient medical record* [tiab] OR clinical sign* [tiab] OR clinical symptom* [tiab] OR "Diagnostic Imaging"[Mesh] OR ultrasound* [tiab] OR ultrasonograph* [tiab] OR ct [tiab] OR pet [tiab] OR mri [tiab] OR mra [tiab] OR magnetic resonance [tiab] OR computed tomograph* [tiab] OR positron emission tomograph* [tiab] OR positron-emission tomograph* [tiab] OR artery biops* [tiab])<br>NOT ("Case Reports" [Publication Type] OR case report* [tiab])
Limits	English language
Number of hits	1347

B. Search Strategy in Embase		
Search number	Search strategy (performed on April 5, 2020)	Number of hits
#1	('giant cell arteritis'/exp OR 'giant cell arteritis':ab,ti OR 'temporal arteritis':ab,ti OR 'hortons disease':ab,ti) AND (('physical examination'/exp OR 'anamnesis'/exp OR 'sensitivity and specificity'/exp OR 'diagnostic procedure'/exp OR 'physical exam*':ab,ti OR sensitiv*:ab,ti OR specificit*) AND ab,ti OR accura*:ab,ti OR 'patient medical record*':ab,ti OR 'clinical sign*':ab,ti OR 'clinical symptom*':ab,ti OR ultrasound*:ab,ti OR ultrasonograph*:ab,ti OR ct:ab,ti OR pet:ab,ti OR mri:ab,ti OR mra:ab,ti OR 'magnetic resonance':ab,ti OR 'computed tomograph*':ab,ti OR 'positron emission tomograph*':ab,ti OR 'positron- emission tomograph*':ab,ti OR 'artery biops*':ab,ti) NOT ('case report'/exp OR 'case report*':ab,ti) NOT [medline]/lim	1064
#2	('giant cell arteritis'/exp OR 'giant cell arteritis':ab,ti OR 'temporal arteritis':ab,ti OR 'hortons disease':ab,ti) AND (('physical examination'/exp OR 'anamnesis'/exp OR 'sensitivity and specificity'/exp OR 'diagnostic procedure'/exp OR 'physical exam*':ab,ti OR sensitiv*:ab,ti OR specificit*) AND ab,ti OR accura*:ab,ti OR 'patient medical record*':ab,ti OR 'clinical sign*':ab,ti OR 'clinical symptom*':ab,ti OR ultrasound*:ab,ti OR ultrasonograph*:ab,ti OR ct:ab,ti OR pet:ab,ti OR mri:ab,ti OR mra:ab,ti OR 'magnetic resonance':ab,ti OR 'computed tomograph*':ab,ti OR 'positron emission tomograph*':ab,ti OR 'positron- emission tomograph*':ab,ti OR 'artery biops*':ab,ti) NOT ('case report'/exp OR 'case report*':ab,ti) NOT [medline]/lim AND [conference abstract]/lim	877
#3	#1 NOT #2	187
#4	#1 NOT #2 AND [english]/lim	133

C. Search Strategy in the Cochrane Database of Systematic Reviews	
Date	April 5, 2020
Search strategy	'Giant cell arteritis'
Limits	None
Number of hits	1

©2020 American Medical Association. All rights reserved.

eTable 2. Details of Included Studies

Authors / year (No. of patients)	Journal	Period of patient inclusion	Study design	Hospital setting	Specialty identifying patients	Specialty referring patients	Included patients	Consecutive patients	Avoiding case control	Lab results reported before treatment	Type of reference standard	Test performed in every patient with clinical diagnosis	Focus of diagnostic testing
Hop et al. 2020 (n = 113)	Rheumatology	January 2013 to November 2017	Retrospective	Academic	Central imaging registry	Unclear	Patients undergoing ultrasound	Yes	Yes	Unclear	Clinical diagnosis	None	Cranial and systemic arteries
Van der Geest et al. 2020 (n = 89)	Annals of the Rheumatic Diseases	June 2010 to December 2013	Prospective	Academic	Rheumatology department	Unclear	Patients referred for clinical evaluation	Yes	Yes	(NA)	Clinical diagnosis	TAB and US	Cranial and systemic arteries
Imfeld et al. 2020 (n =102)	Rheumatology	December 2006 to August 2012	Prospective	Academic	Multiple hospital departments	Unclear	Patients undergoing ultrasound and PET/CT	Yes	Yes	(NA)	Clinical diagnosis	Ultrasound and PET/CT	Cranial and systemic arteries
Mukhtyar et al. 2020 (n = 23)	Clinical Rheumatology	March 2013 to (?)	Retrospective	Academic	Multiple hospital departments	Unclear	Patients undergoing TAB and ultrasound	Yes	Yes	Yes	Clinical diagnosis (+TAB) (+US)	TAB and US	Cranial and systemic arteries
Sammel et al. 2019 (n = 58)	Rheumatology	May 2016 to December 2017	Prospective	Academic	Rheumatology department	Unclear	Patients referred for clinical evaluation	Yes	Yes	(NA)	Clinical diagnosis (+TAB)	TAB	Cranial arteries
Nielsen et al. 2019 (n = 90)	Rheumatology	October 2014 to June 2018	Prospective	Academic	Rheumatology department	Unclear	Patients referred for clinical evaluation	Yes	Yes	(NA)	Clinical diagnosis	TAB and PET/CT	Cranial and systemic arteries
Sundholm et al. 2019 (n = 75)	Rheumatology Advances in Practice	Augustus 2015 to May 2018	Prospective	Academic	Central pathology/surgery registry ('Vascular surgery unit')	Unclear	Patients undergoing TAB	Yes	Yes	No	Clinical diagnosis (+TAB)	TAB	Cranial and systemic arteries
Sommer et al. 2019 (n = 68)	Clinical and Experimental Ophthalmology	2015 to 2017	Retrospective	Academic	Ophthalmology department	Hospital departments	Patients undergoing TAB	Yes	Yes	Unclear	TAB	(NA)	Cranial arteries
Gospe et al. 2019 (n = 13)	Journal of Neuro-Ophthalmology	January 2009 to October 2014	Retrospective	Academic	Central pathology/surgery registry and central imaging registry	Unclear	Patients undergoing TAB and MRI	Yes	Yes	Unclear	TAB	(NA)	Cranial arteries
Ing et al. 2019 (n = 1833)	Clinical Ophthalmology	January 2006 to June 2018	Retrospective	Non-academic and Academic	Central pathology/surgery registry	Primary care and hospital departments	Patients undergoing TAB	Yes	Yes	(NA)	TAB	(NA)	Cranial arteries
Oiwa et al. 2019 (n = 29)	Internal Medicine	April 2009 to October 2018	Retrospective	Non-academic	Central pathology/surgery registry	Unclear	Patients undergoing TAB	Yes	Yes	Unclear	Clinical diagnosis (+ TAB)	TAB	Cranial arteries
Conway et al. 2019 (n = 162)	Clinical and Experimental Rheumatology	August 2011 to December 2016	Prospective	Academic	Rheumatology department	Unclear	Patients referred for clinical evaluation	Yes	Yes	(NA)	Clinical diagnosis	TAB and US	Cranial arteries
Chan et al. 2019 (n = 270)	BMC Rheumatology	January 2011 to December 2014	Retrospective	Academic	Central pathology/surgery registry	Unclear	Patients undergoing TAB	Yes	Yes	Unclear	Clinical diagnosis	TAB	Cranial arteries
Hay et al. 2019 (n = 63)	Annals of Nuclear Medicine	January 2007 to January 2017	Retrospective	Academic	Central imaging registry	Unclear	Patients undergoing PET-CT (with TAB neg)	Yes	Yes	(NA)	Clinical diagnosis	TAB and PET-CT	Systemic arteries
Oh et al. 2018 (n = 537)	Internal Medicine Journal	January 1992 to December 2015	Retrospective	Non-academic and Academic	Central pathology/surgery registry	Unclear	Patients undergoing TAB	Yes	Yes	Unclear	TAB	(NA)	Cranial arteries

©2020 American Medical Association. All rights reserved.

Ing et al. 2018 (n = 109)	Canadian Journal of Ophthalmology	March 2015 to April 2017	Prospective	Academic	Ophthalmology department	Primary care and hospital departments	Patients undergoing TAB	Yes	Yes	(NA)	TAB	(NA)	Cranial arteries
Bilyk et al. 2018 (n = 71)	Transactions of the American Ophthalmological Society	14 months period'	Prospective	Academic	Ophthalmology department	Unclear	Patients referred for clinical evaluation	Yes	Yes	Unclear	TAB	(NA)	Cranial arteries
Monti et al. 2017 (n = 202)	Rheumatology	July 2014 to September 2016	Retrospective	Academic	Rheumatology department	Primary care and hospital departments	Patients referred for clinical evaluation	Yes	Yes	No	Clinical diagnosis	US	Cranial and systemic arteries
Czihal et al. 2017 (n = 92)	Clinical and Experimental Rheumatology	October 2014 to October 2015	Retrospective	Academic	Multiple hospital departments	Unclear	Patients undergoing ultrasound	Yes	Yes	No	Clinical diagnosis	None	Cranial and systemic arteries
Roncato et al. 2017 (n = 42)	Clinical and Experimental Rheumatology	April 2009 to March 2014	Retrospective	Non-academic	Central pathology/surgery registry and central imaging registry	Unclear	Patients undergoing TAB and ultrasound	Yes	Yes	(NA)	Clinical diagnosis	TAB and US	Cranial and systemic arteries
Toren et al. 2016 (n = 250)	Canadian Journal of Ophthalmology	"a 3 year period"	Prospective	Academic	Ophthalmology department	Primary care and hospital departments	Patients undergoing TAB	Yes	Yes	No	TAB	(NA)	Cranial arteries
Grossman et al. 2016 (n = 224)	Clinical Rheumatology	2000 to 2014	Retrospective	Academic	Central pathology/surgery registry	Unclear	Patients undergoing TAB	Yes	Yes	Unclear	Clinical diagnosis (+ TAB)	TAB	Cranial arteries
Luqmani et al. 2016 (n = 381)	Health Technology Assessment	June 2010 to December 2013	Prospective	Non-academic and Academic	Multiple hospital departments	Unclear	Patients undergoing TAB	Yes	Yes	Yes	Clinical diagnosis	TAB and US	Cranial and systemic arteries
Lariviere et al. 2016 (n = 24)	Medicine	November 2013 to August 2015	Prospective	Academic	Central pathology/surgery registry	Unclear	Patients undergoing TAB	Yes	Yes	Unclear	Clinical diagnosis	TAB, PET/CT and CTA	Cranial and systemic arteries
De Lott et al. 2015 (n = 404)	JAMA Ophthalmology	January 2007 to April 2012	Retrospective	Non-academic and Academic	Ophthalmology department	Unclear	Patients undergoing TAB	Yes	Yes	Unclear	TAB	(NA)	Cranial arteries
Stacy et al. 2015 (n = 58)	Journal of Neuro-Ophthalmology	January 2008 to April 2013	Retrospective	Academic	Ophthalmology department	Unclear	Patients undergoing TAB	Yes	Yes	(NA)	TAB	(NA)	Cranial arteries
El-Dairi et al. 2015 (n = 192)	Journal of Neuro-Ophthalmology	2000 to 2009	Retrospective	Academic	Central pathology/surgery registry	Unclear	Patients undergoing TAB	Yes	Yes	No	TAB	(NA)	Cranial arteries
Croft et al. 2015 (n = 87)	Journal of the Royal College of Physicians of Edinburgh	January 2005 to January 2014	Retrospective	Academic	Central imaging registry	Unclear	Patients undergoing ultrasound	Yes	Yes	(NA)	Clinical diagnosis	US	Cranial and systemic arteries
Aschwanden et al. 2015 (n = 60)	Clinical and Experimental Rheumatology	October 2011 to December 2012	Prospective	Academic	Multiple hospital departments	Unclear	Patients referred for clinical evaluation	Yes	Yes	(NA)	Clinical diagnosis	None	Cranial arteries
Knecht et al. 2015 (n = 31)	Eye	October 2007 to March 2009	Prospective	Academic	Ophthalmology department	Unclear	Patients undergoing TAB	Yes	Yes	(NA)	TAB	(NA)	Cranial arteries
Diamantopoulos et al. 2014 (n = 88)	Arthritis Care & Research	April 2010 to October 2012	Retrospective	Non-academic	Rheumatology department	Primary care and hospital departments	Patients referred for clinical evaluation	Yes	Yes	(NA)	Clinical diagnosis	None	Cranial and systemic arteries
Gonzalez-Lopez et al. 2013 (n = 335)	Acta Ophthalmologica	January 2001 to December 2010	Retrospective	Academic	Central pathology/surgery registry	Unclear	Patients undergoing TAB	Yes	Yes	(NA)	TAB	(NA)	Cranial arteries
Aschwanden et al. 2013 (n = 80)	Ultraschall in der Medizin	March 2009 to September 2011	Prospective	Academic	Multiple hospital departments	Unclear	Patients undergoing ultrasound	Yes	Yes	(NA)	Clinical diagnosis	None	Cranial arteries
Black et al. 2013 (n = 46)	International Journal of Rheumatic Diseases	September 2003 to September 2011	Retrospective	Academic	Central imaging registry	Primary care and hospital departments	Patients undergoing ultrasound	Yes	Yes	(NA)	Clinical diagnosis	US	Cranial arteries

©2020 American Medical Association. All rights reserved.

Suelves et al. 2013 (n = 38)	Archivos de la Sociedad Española de Oftalmología	April 2004 to January 2010	Retrospective	Academic	Ophthalmology department	Unclear	Patients undergoing TAB	Yes	Yes	Unclear	TAB	(NA)	Cranial arteries
Kermani et al. 2012 (n = 764)	Seminars in Arthritis and Rheumatism	January 2000-December 2008	Retrospective	Academic	Central pathology/surgery registry	Unclear	Patients undergoing TAB	Yes	Yes	No	TAB	(NA)	Cranial arteries
Quinn et al. 2012 (n = 172)	Annals of Vascular Surgery	January 1990 to December 2010	Retrospective	Academic	Central pathology/surgery registry	Unclear	Patients undergoing TAB	Yes	Yes	(NA)	TAB	(NA)	Cranial arteries
Habib et al. 2012 (n = 32)	Clinical Rheumatology	January 2008 to January 2010	Prospective	Academic	Multiple hospital departments	Unclear	Patients referred for clinical evaluation	Yes	Yes	Unclear	Clinical diagnosis	TAB and US	Cranial arteries
Lugo et al. 2011 (n = 138)	Journal of Surgical Research	2002 to 2009	Retrospective	Academic	Central pathology/surgery registry	Unclear	Patients undergoing TAB	Yes	Yes	Unclear	TAB	(NA)	Cranial arteries
Walvick et al. 2011 (n = 2441)	Ophthalmology	1997 to 2006	Retrospective	Unclear (Medical consortium group)	Central pathology/surgery registry	Unclear	Patients undergoing TAB	Yes	Yes	Unclear	TAB	(NA)	Cranial arteries
Mari et al. 2009 (n = 278)	European Journal of Internal Medicine	January 1989 to December 2007	Retrospective	Non-academic	Central pathology/surgery registry	Unclear	Patients undergoing TAB	Yes	Yes	Yes	TAB	(NA)	Cranial arteries
Ghinoi et al. 2008 (n = 20)	Clinical and Experimental Rheumatology	April 2005 to April 2006	Prospective	Academic	Rheumatology department	Unclear	Patients referred for clinical evaluation	Yes	Yes	(NA)	TAB	(NA)	Cranial arteries
Moutray et al. 2008 (n = 50)	Canadian Journal of Ophthalmology	1995 to 2001	Retrospective	Academic	Ophthalmology department	Hospital department	Patients undergoing TAB	Yes	Yes	(NA)	TAB	(NA)	Cranial arteries
Bley et al. 2008 (n = 59)	Arthritis & Rheumatism	May 2003 to February 2007	Retrospective	Academic	Central imaging registry	Unclear	Patients undergoing MRI and ultrasound	Yes	Yes	(NA)	Clinical diagnosis	None	Cranial arteries
Hautzel et al. 2008 (n = 23)	Journal of Nuclear Medicine	October 2003 to August 2007	Retrospective	Academic	Central imaging registry	Unclear	Patients undergoing PET	Yes	Yes	Unclear	Clinical diagnosis	None	Cranial and systemic arteries
Rodriguez-Pla et al. 2007 (n = 125)	Scandinavian Journal of Rheumatology	January 1997 to March 2002	Retrospective	Academic	Central pathology/surgery registry	Primary	Patients undergoing TAB	Yes	Yes	Unclear	TAB	(NA)	Cranial arteries
Karahaliou et al. 2006 (n = 55)	Arthritis Research & Therapy	2000 to 2004	Prospective	Academic	Multiple hospital departments	Unclear	Patients referred for clinical evaluation	Yes	Yes	Unclear	Clinical diagnosis	None	Cranial arteries
Bley et al. 2005 (n = 21)	Arthritis & Rheumatism	Unclear (1 year time span)	Prospective	Academic	Central imaging registry	Unclear	Patients undergoing MRA	Yes	Yes	Yes	Clinical diagnosis	None	Cranial arteries
Younge et al. 2004 (n = 1113)	Mayo Clinic Proceedings	January 1988 to December 1997	Retrospective	Academic	Central pathology/surgery registry	Unclear	Patients undergoing TAB	Yes	Yes	No	TAB	(NA)	Cranial arteries
Varma et al. 2004 (n = 53)	Eye	January 1995 to December 2000	Retrospective	Academic	Ophthalmology department	Unclear	Patients undergoing TAB	Yes	Yes	Unclear	TAB	(NA)	Cranial arteries
Hall et al. 2003 (n = 181)	Ophthalmology	January 1990 to January 2001	Retrospective	Academic	Multiple hospital departments	Unclear	Patients undergoing TAB	Yes	Yes	Unclear	TAB	(NA)	Cranial arteries
Foroozan et al. 2002 (n = 91)	Ophthalmology	January 1992 to December 1999	Retrospective	Academic	Ophthalmology department	Unclear	Patients undergoing TAB	Yes	Yes	No	TAB	(NA)	Cranial arteries
Mohamed et al. 2002 (n = 50)	Annals of The Royal College of Surgeons of England	January 1988 to December 1997	Retrospective	Non-academic	Central pathology/surgery registry	Unclear	Patients undergoing TAB	Yes	Yes	Unclear	TAB	(NA)	Cranial arteries
Grosser et al. 1999 (n = 120)	Neuro-Ophthalmology	1986 to 1995	Retrospective	Academic	Central pathology/surgery registry	Unclear	Patients undergoing TAB	Yes	Yes	(NA)	TAB	(NA)	Cranial arteries
Hayreh et al. 1997 (n = 363)	American Journal of Ophthalmology	1973 to 1994	Prospective	Academic	Central pathology/surgery registry	Hospital departments	Patients undergoing TAB	Yes	Yes	Unclear	TAB	(NA)	Cranial arteries

©2020 American Medical Association. All rights reserved.

Gabriel et al. 1995 (n = 525)	Journal of Rheumatology	January 1988 to December 1991	Retrospective	Academic	Central pathology/surgery registry	Unclear	Patients undergoing TAB	Yes	Yes	(NA)	TAB	(NA)	Cranial arteries
Skaug et al. 1995 (n = 98)	Acta Ophthalmologica Scandinavica	January 1984 to December 1992	Retrospective	Academic	Ophthalmology department	Primary care and hospital departments	Patients undergoing TAB	Yes	Yes	Unclear	Clinical diagnosis (+ TAB)	TAB	Cranial arteries
Chmelewski et al. 1992 (n = 98)	Archives of Internal Medicine	January 1985 to March 1990	Retrospective	Academic	Central pathology/surgery registry	Unclear	Patients undergoing TAB	Yes	Yes	Unclear	TAB	(NA)	Cranial arteries
Brittain et al. 1991 (n = 31)	British Journal of Ophthalmology	Unclear (54-month period)	Prospective	Academic	Ophthalmology department	Unclear	Patients undergoing TAB	Yes	Yes	Yes	TAB	(NA)	Cranial arteries
Kent et al. 1989 (n = 70)	The American Surgeon	January 1980 to January 1985	Retrospective	Non-academic	Central pathology/surgery registry	Unclear	Patients undergoing TAB	Yes	Yes	(NA)	TAB	(NA)	Cranial arteries
Stuart 1989 (n = 75)	New Zealand Medical Journal	1983 to 1987	Retrospective	Academic	Central pathology/surgery registry	Primary care and hospital departments	Patients undergoing TAB	Yes	Yes	Unclear	TAB	(NA)	Cranial arteries
Fernandez-Herlihy 1988 (n = 107)	Journal of Rheumatology	October 1982 to September 1987	Retrospective	Non-academic	Central pathology/surgery registry	Unclear	Patients undergoing TAB	Yes	Yes	Unclear	TAB	(NA)	Cranial arteries
Wells et al. 1989 (n = 100)	Ophthalmology	January 1985 to June 1987	Retrospective	Academic	Ophthalmology department	Unclear	Patients undergoing TAB	Yes	Yes	(NA)	TAB	(NA)	Cranial arteries
Vilaseca et al. 1987 (n = 103)	Annals of the Rheumatic Diseases	1970 to 1984	Retrospective	Academic	Central pathology/surgery registry	Unclear	Patients undergoing TAB	Yes	Yes	Unclear	TAB	(NA)	Cranial arteries
Roth et al. 1984 (n = 51)	Archives of Ophthalmology	Unclear (last 16 years)	Retrospective	Academic	Central pathology/surgery registry	Unclear	Patients undergoing TAB	Yes	Yes	(NA)	Clinical diagnosis (+ TAB)	TAB	Cranial arteries
Hall et al. 1983 (n = 134)	The Lancet	January 1965 to December 1980	Retrospective	Academic	Central pathology/surgery registry	Unclear	Patients undergoing TAB	Yes	Yes	(NA)	TAB	(NA)	Cranial arteries
Hedges et al. 1983 (n = 91)	Archives of Ophthalmology	January 1968 to December 1978	Retrospective	Academic	Central pathology/surgery registry	Unclear	Patients undergoing TAB	Yes	Yes	Unclear	Clinical diagnosis	TAB	Cranial arteries
Eshagian et al. 1980 (n = 66)	Ophthalmology	Unclear	Retrospective	Academic	Unclear	Unclear	Patients undergoing TAB	Yes (likely)	Yes (likely)	Yes	TAB	(NA)	Cranial arteries

Information is provided for all 68 included studies [1-68]. TAB = temporal artery biopsy. US = ultrasound. (NA) = not applicable.

©2020 American Medical Association. All rights reserved.

eTable 3. Studies With TAB as Reference Standard

TAB	No. of studies (% of studies)
**Bilaterally performed**	
0-24% of patients	14 (37%)
25-49% of patients	4 (11%)
50-74% of patients	2 (5%)
75-100% of patients	2 (5%)
Unclear	16 (42%)
**Mean or median TAB length > 1 cm**	
No	1 (3%)
Yes	23 (60%)
Unclear	14 (37%)

Additional information on the 38 studies with TAB as the reference standard.

©2020 American Medical Association. All rights reserved.

eTable 4. Studies With Clinical Diagnosis as Reference Standard

**Clinical diagnosis**	**No, of studies (% of studies)**
**Follow-up performed**	
No	8 (27%)
At least 3 months	4 (13%)
At least 6 months	12 (40%)
Unclear	6 (20%)
**Test performed in every patient**	
None	9 (30%)
TAB	8 (27%)
Ultrasound	3 (10%)
TAB + ultrasound	6 (20%)
TAB + PET/CT and/or CTA	3 (10%)
Ultrasound and PET/CT	1 (3%)

Additional information on the 30 studies with clinical diagnosis as reference standard.

©2020 American Medical Association. All rights reserved.

eTable 5. Study-Specific Definitions of Clinical Diagnosis

Study	Definition of clinical diagnosis	Test result known for every patient
Hop et al. 2020 [32]	Two clinical experts independently assessed whether or not GCA was the final clinical diagnosis ***after at least 6 months*** in all included patients. They reviewed the complete history of the patients, including cranial and systemic symptoms, laboratory findings, findings at additional diagnostic tests (TAB, FDG-PET/CT or MRI) and response to glucocorticoids, but not the CDU data.	none
Imfeld et al. 2020 [33]	The final diagnosis of GCA was made either if temporal artery biopsy was positive, if patients fulfilled the 1990 ACR criteria or if they fulfilled at least two out of five ACR criteria in combination with typical 'vasculitic' US findings in accordance to the OMERACT definition or vasculitic findings in PET/CT or MRI.	Ultrasound and PET/CT
van der Geest et al. 2020 [63]	The final clinical diagnosis was made by the treating physician ***after 6 months follow-up***. The clinical diagnosis was guided by the ACR 1990 classification criteria for GCA (including the temporal artery biopsy result), the development of complications consistent with GCA during follow-up, and the emergence of another disease explaining the symptoms.	TAB and ultrasound
Mukhtyar et al. 2020 [47]	Clinical decisions were recorded as GCA if clinicians chose to treat patients with the hospital-approved Norwich regimen for prednisolone. The final diagnosis was determined ***after 100 weeks of follow-up***.	TAB and ultrasound
Chan et al. 2019 [8]	Final clinical diagnosis was at the discretion of the treating physician and in biopsy negative cases these were made based upon suggestive clinical features and clinical response to glucocorticoid therapy. The diagnosis was reviewed after treatment and ***follow up period of at least 3 months***.	TAB
Conway et al. 2019 [10]	Consultant rheumatologist diagnosis ***at 6 months following initial presentation*** was considered as the reference standard for the diagnosis of GCA for the purposes of this study.	TAB and ultrasound
Hay et al. 2019 [29]	The final diagnosis of GCA was established during follow-up based on the presence of clinical symptoms, laboratory results and imaging data (CT, MRI, 18F-FDG PET-CT) compatible with GCA, good response to corticosteroid therapy (from a clinical and biological point of view), and no differential diagnosis after a ***follow-up of at least 18 months***.	TAB and PET/CT
Nielsen et al. 2019 [48]	An experienced rheumatologist performed a pre-treatment clinical evaluation to confirm eligibility criteria and establish the clinical diagnosis. The evaluation included history taking, physical examination, extensive laboratory screening, the FDG PET/CT report and TAB. US was not considered for establishing the clinical diagnosis. A clinical GCA diagnosis could be dismissed, even in patients fulfilling ACR criteria, if another more reasonable diagnosis was established. The clinical diagnosis of GCA was confirmed at a ***6-month follow-up*** visit in 51/56 patients. Five patients were lost to follow-up, all of whom were TAB positive.	TAB and PET/CT
Oiwa et al. 2019 [50]	The inclusion criteria for the GCA group were 1) new-onset cases with a clinical diagnosis of GCA and 2) no change in the diagnosis of GCA until the final observation. All of the cases that did not meet these criteria were defined as the non-GCA group.	TAB
Sammel et al. 2019 [55]	The clinical diagnosis was confirmed after a ***minimum of 6 months of follow-up*** based on the biopsy, corticosteroid dose at 3 months, treating clinician and expert reviewer diagnoses. Clinicians and the expert reviewers were blinded to PET/CT vascular findings. Reviewers included five rheumatologists and one neuro-ophthalmologist not involved in the patient's care. The final diagnosis required consensus between the treating clinician and at least one of two reviewers.	TAB
Sundholm et al. 2019 [61]	Patient records were screened for a ***period of 6 months after biopsy***. The final diagnosis was set by rheumatologists based on symptoms, clinical signs, laboratory findings, biopsy, evidence of large vessel vasculitis on PET CT or MRI, treatment response and, in the case of an uncertain diagnosis, a careful differential diagnostic work-up during follow-up.	TAB

©2020 American Medical Association. All rights reserved.

Czihal et al. 2017 [12]	The final clinical diagnosis was based on the American College of Rheumatology classification criteria and/or a positive temporal artery biopsy (TAB) for cranial GCA and on non-invasive cross sectional imaging (magnetic resonance imaging or positron emission tomography) for extracranial GCA.	none
Monti et al. 2017 [45]	Expert rheumatologist made a final clinical decision. In cases with both negative ultrasound and TAB, the diagnosis was based on clinical presentation, laboratory results, response to treatment and, when available, other imaging modalities (mainly PET-CT).	ultrasound
Roncato et al. 2017 [53]	Final diagnosis of GCA was retained for patients who improved or were cured by corticosteroid therapy according to the follow-up data. Alternative diagnoses were defined by medical re-view of patient files.	TAB and ultrasound
Grossman et al. 2016 [24]	Diagnosis of biopsy-proven GCA required the histological findings of interruption of the internal elastic laminate with infiltration of mononuclear cells into the arterial wall. Patients were diagnosed with TAB-negative GCA based on clinical judgment of the treating physician, provided the patient's symptoms and signs improved within 3 days of corticosteroid treatment (40 mg of prednisone or more), no other better alternative diagnosis could be reached after a thorough evaluation and clinical follow-up, and the patients fulfilled the ACR 1990 classification criteria for GCA	TAB
Lariviere et al. 2016 [40]	The American College of Rheumatology (ACR) 1990 GCA criteria were used for classification. The diagnosis of GCA was established on an individual basis by experienced clinicians. Of note, clinical judgment included short-term outcome following corticosteroid therapy, such as rapid and dramatic improvement of clinical symptoms and normalization of the C-reactive protein (CRP) blood level. TAB was performed in all patients. In patients with negative TAB results, the clinical diagnosis was considered final if no diagnosis other than GCA was provided at the end of a ***follow-up period of >6 months***.	TAB, PET/CT and CTA
Luqmani et al. 2016 [42]	For the purposes of the study, a partially independent approach was used, which combined elements of a clinician's final diagnosis, the ACR classification criteria (incorporating the biopsy result), the emergence of complications consistent with GCA during follow-up, the emergence of alternative vasculitis diagnoses during follow-up and expert review to determine the reference diagnosis. The process started with the clinician's final diagnosis for the patient as reported on the 6-month (or in its absence, 2-week) assessment. *Note: data on index tests was obtained with the clinical diagnosis at 2 weeks follow-up as the reference standard. For the current systematic review, only a follow-up duration of 3 months or more was considered relevant.*	TAB and ultrasound
Aschwanden et al. 2015 [2]	The diagnosis of GCA was established based on the ACR criteria.	none
Croft et al. 2015 [11]	For the purposes of this study, a clinical diagnosis of GCA made by a consultant rheumatologist, alone or in collaboration with other specialists, after a ***minimum of 3 months of follow-up***, served as the reference or 'gold' standard for a diagnosis of GCA. The American College of Rheumatology (ACR) criteria for GCA were also used to classify all cases for comparison	ultrasound
Diamantopoulos et al. 2014 [14]	As the gold diagnostic standard, we used the positive clinical evaluation for GCA at ***6 months after the initial evaluation*** performed by 3 rheumatologists. The 3 physicians made their diagnostic decision without knowing the results of the CDUS findings. Each assessed patient was mainly judged by 1 rheumatologist and not by all 3. Patients were also classified according to ACR 1990 criteria for the classification of GCA.	none
Aschwanden et al. 2013 [1]	Definite diagnosis after extensive clinical, laboratory and imaging evaluation was established by a team of independent and experienced rheumatologists/immunologists not involved in CDU according to ACR criteria	none
Black et al. 2013 [4]	Patients were divided into two groups depending on whether a diagnosis of GCA was or was not made by the treating doctor.	ultrasound
Habib et al. 2012 [25]	Regardless the TAB results, all patients had to complete a ***3-months follow-up*** to establish final diagnosis.	TAB and ultrasound

©2020 American Medical Association. All rights reserved.

Bley et al. 2008 [6]	The clinical diagnosis of GCA was established by experienced rheumatologists. The ACR 1990 GCA criteria were used for classification. The rheumatologist's final clinical diagnosis was assigned if the diagnosis of GCA was confirmed at the end of a ***follow-up period of ≥6 months***.	none
Hautzel et al. 2008 [28]	GCA was finally confirmed by an additional diagnostic work-up including biopsies, duplex sonography, and other angiographic diagnostics. In most patients with 3 or more of the ACR criteria and positive findings on duplex sonography (which further support the diagnosis of GCA), a temporal artery biopsy was not performed.	none
Karahaliou et al. 2006 [36]	Irrespectively of the biopsy results, patients had to complete a ***3-month follow-up*** to establish the final diagnosis.	none
Bley et al. 2005 [5]	Clinical diagnosis was made according to the ACR criteria for GCA.	none
Skaug et al. 1995 [56]	According to the clinical findings and the results of the biopsy, the patients were divided into three groups. Group I represents patients with histologically confirmed GCA. Group 2 included patients with a good clinical evidence of GCA despite a negative biopsy, whereas in group 3 were patients where the biopsy was negative and the clinical examination revealed either other diseases than GCA, or the etiology of the symptoms remained unclear.	TAB
Roth et al. 1984 [54]	The patients were divided into three groups depending on the biopsy results and on their response to steroid therapy. (A positive response to therapy was considered to be resolution of symptoms within 48 hours and reduction of the ESR within three weeks after the start of treatment.) Group 1 comprised those patients who had abnormal biopsy specimens and were clinically responsive to treatment. The condition of these patients was diagnosed as GCA. Group 2 consisted of those patients who had normal biopsy specimens but who were clinically responsive to treatment. Presumed GCA was diagnosed for these patients. Group 3 contained those patients with normal biopsy specimens who were clinically unresponsive to treatment. These patients were considered not to have GCA and their ultimate diagnoses were determined from the chart review.	TAB
Hedges et al. 1983 [31]	Examination of available clinical information from these cases showed that were 91 cases in which there was an adequate proof (record) of the history taken when the patient was initially seen and the physical examination, and in which **follow-up of at least two years** could be obtained by chart review and by contacting the patients, their families and their physicians.<br>[…]<br>In addition to making the diagnosis of temporal arteritis based on a positive temporal artery biopsy specimen, the diagnosis of arteritis was accepted in patients who had an initial negative biopsy specimen either on the basis of subsequent pathologic proof of the disease or when the clinical course during the follow-up period was consistent with temporal arteritis. Patients with a negative temporal artery biopsy specimen were not considered to have the disease if there was resolution of their symptoms and signs within a one-month period, or if their symptoms and signs (including abnormal laboratory test results) were subsequently found to be due to other diseases.	TAB

Details regarding the clinical diagnosis are provided for all 30 studies using this reference standard. Information on follow-up is highlighted in bold.
TAB = temporal artery biopsy.

©2020 American Medical Association. All rights reserved.

eTable 6. Symptoms With Limited Data Available

Symptom	Study (reference standard)	No. of patients (No. ref+)	Sensitivity (95% CI)	Specificity (95% CI)	LR+ (95% CI)	LR- (95% CI)
Arthritis	Gabriel et al. 1995 (TAB)	525 (172)	5.8 (2.8-10.4)	84.7 (80.5-88.3)	0.38 (0.20-0.71)	1.11 (1.05-1.18)
	Stuart et al. 1989 (TAB)	75 (14)	7.1 (0.2-33.9)	83.6 (71.9-91.9)	0.44 (0.07-2.19)	1.11 (0.81-1.31)
	Vilaseca et al. 1987 (TAB)	103 (45)	6.7 (1.4-18.3)	87.9 (76.7-95.0)	0.55 (0.16-1.84)	1.06 (0.92-1.22)
	Pooled	*703 (231)*	*6.1 (3.4-10.0)*	*85.0 (81.4-88.1)*	*0.41 (0.24-0.72)*	*1.10 (1.05-1.16)*
Cough	Nielsen et al. 2019 (Clin)	90 (56)	42.9 (29.7-56.8)	82.4 (65.5-93.2)	2.43 (1.18-5.40)	0.69 (0.52-0.92)
	Oiwa et al. 2019 (Clin)	28 (16)	31.3 (11.0-58.7)	100.0 (73.5-100.0)	8.41 (0.98-84.22)	0.69 (0.46-1.03)
	Lariviere et al. 2016 (Clin)	24 (15)	6.7 (0.2-32.0)	77.8 (40.0-97.2)	0.30 (0.04-2.07)	1.20 (0.85-2.08)
	Pooled	*(-)*	*(-)*	*(-)*	*(-)*	*(-)*
Neck pain	Bley et al. 2008 (Clin)	59 (36)	25.0 (12.1-42.2)	91.3 (72.0-98.9)	2.88 (0.80-11.29)	0.82 (0.63-1.06)
	Younge et al. 2004 (TAB)	1113 (373)	9.9 (7.1-13.4)	96.6 (95.1-97.8)	2.94 (1.80-4.78)	0.93 (0.89-0.96)
	Hayreh et al. 1997 (TAB)	363 (106)	16.0 (9.6-24.4)	95.7 (92.5-97.8)	3.75 (1.84-7.60)	0.88 (0.79-0.94)
	Pooled	*1535 (515)*	*12.2 (9.5-15.4)*	*96.3 (94.9-97.4)*	*3.15 (2.13-4.66)*	*0.91 (0.86-0.96)*
Neurological symptoms	Mohamed et al. 2002 (TAB)	50 (17)	17.7 (3.8-43.4)	66.7 (48.2-82.0)	0.53 (0.17-1.45)	1.24 (0.84-1.73)
	Chmelewski et al. 1992 (TAB)	98 (30)	16.7 (5.6-34.7)	66.2 (53.7-77.2)	0.49 (0.21-1.09)	1.26 (0.97-1.59)
	Pooled	*148 (47)*	*17.0 (7.6-30.8)*	*66.3 (56.2-75.4)*	*0.51 (0.25-1.01)*	*1.25 (1.04-1.51)*
Night sweats	Hop et al. 2020 (Clin)	113 (41)	34.2 (20.1-50.6)	91.7 (82.7-96.9)	4.10 (1.76-9.66)	0.72 (0.55-0.88)
	Sundholm et al. 2019 (Clin)	78 (36)	69.4 (51.9-83.7)	40.5 (25.6-56.7)	1.17 (0.83-1.64)	0.75 (0.40-1.37)
	Pooled	*(-)*	*(-)*	*(-)*	*(-)*	*(-)*
Tongue claudication	Van der Geest et al. 2020 (Clin)	89 (58)	3.4 (0.4-11.9)	96.8 (83.3-99.9)	1.07 (0.15-8.03)	1.00 (0.91-1.16)
	Monti et al. 2017 (Clin)	202 (110)	1.8 (0.2-6.4)	100.0 (96.1-100.0)	9.47 (0.87-104.42)	0.98 (0.94-1.03)
	Hall et al. 1983 (TAB)	134 (46)	4.4 (0.5-14.8)	100.0 (95.9-100.0)	4.19 (0.38-46.43)	0.96 (0.86-1.01)
	Pooled	*425 (214)*	*2.8 (1.0-6.0)*	*99.5 (97.4-100.0)*	*2.84 (0.58-13.82)*	*0.98 (0.95-1.01)*

Data are shown for symptoms reported by less than four studies. In this case, meta-analysis with the bivariate model could not be performed. Therefore, pooled estimates of diagnostic accuracy parameters were determined with an univariate random-effects model (DerSimonian Laird method) [69]. Pooled estimates are only shown if heterogeneity (I^2) was <75% for all diagnostic accuracy parameters [70]. Pooling of diagnostic accuracy parameters was performed with MetaDiSc 1.4. Diagnostic accuracy parameters of single studies were determined with StatsDirect 3.2.10. Clin = clinical diagnosis. TAB = temporal artery biopsy. No. ref+ = number of patients with positive reference standard for GCA. 95% CI = 95% confidence interval. (-) No pooled estimates reported due to heterogeneity ≥ 75% for at least one diagnostic accuracy parameter.

©2020 American Medical Association. All rights reserved.

eTable 7. Physical Findings With Limited Data Available

Physical finding	Study (reference standard)	No. of patients (No. ref+)	Sensitivity (95% CI)	Specificity (95% CI)	LR+ (95% CI)	LR- (95% CI)
Ocular findings	Van der Geest et al. 2020 (Clin)	89 (58)	20.7 (11.2-33.4)	77.4 (58.9-90.4)	0.92 (0.42-2.09)	1.02 (0.82-1.35)
	Gabriel et al. 1995 (TAB)	525 (172)	10.5 (6.3-16.0)	85.8 (81.8-89.3)	0.74 (0.44-1.21)	1.04 (0.97-1.11)
	Chmelewski et al. 1992 (TAB)	98 (30)	23.3 (9.9-42.3)	77.9 (66.2-87.1)	1.06 (0.48-2.22)	0.98 (0.74-1.22)
	Pooled	*712 (260)*	*14.2 (10.2-19.1)*	*84.1 (80.4-87.3)*	*0.84 (0.58-1.23)*	*1.04 (0.98-1.10)*
Ocular nerve palsy	Luqmani et al. 2016 (Clin)	381 (257)	1.2 (0.2-3.4)	100.0 (97.1-100.0)	3.39 (0.32-36.18)	0.99 (0.97-1.03)
	Moutray et al. 2008 (TAB)	50 (17)	11.8 (1.5-36.4)	93.9 (79.8-99.3)	1.94 (0.36-10.21)	0.94 (0.69-1.13)
	Pooled	*(-)*	*(-)*	*(-)*	*(-)*	*(-)*
PION	Ing et al. 2018 (TAB)	109 (19)	5.3 (0.1-26.0)	100.0 (96.0-100.0)	13.65 (1.14-162.59)	0.95 (0.76-1.01)
	Luqmani et al. 2016 (Clin)	381 (257)	1.6 (0.4-3.9)	97.6 (93.1-99.5)	0.64 (0.16-2.54)	1.01 (0.98-1.06)
	Pooled	*490 (276)*	*1.8 (0.6-4.2)*	*98.6 (96.0-99.7)*	*2.13 (0.11-39.74)*	*0.99 (0.92-1.07)*
RAPD	Bilyk et al. 2018 (TAB)	71 (14)	57.1 (28.9-82.3)	50.9 (37.3-64.4)	1.16 (0.63-1.83)	0.84 (0.40-1.46)
	Luqmani et al. 2016 (Clin)	381 (257)	5.1 (2.7-8.5)	98.4 (94.3-99.8)	3.14 (0.81-12.35)	0.96 (0.93-1.01)
	Pooled	*(-)*	*(-)*	*(-)*	*(-)*	*(-)*
Temporal artery abnormal on palpation	Mari et al. 2009 (TAB)	241 (75)	73.3 (61.9-82.9)	47.6 (39.8-55.5)	1.40 (1.14-1.70)	0.56 (0.37-0.82)
	Bley et al. 2008 (Clin)	59 (36)	83.3 (67.2-93.6)	78.3 (56.3-92.5)	3.83 (1.94-8.72)	0.21 (0.10-0.43)
	Kent et al. 1989 (TAB)	70 (8)	50.0 (15.7-84.3)	88.7 (78.1-95.3)	4.43 (1.55-10.81)	0.56 (0.24-0.90)
	Pooled	*(-)*	*(-)*	*(-)*	*(-)*	*(-)*

Data are shown for physical findings reported by less than four studies. In this case, meta-analysis with the bivariate model could not be performed. Therefore, pooled estimates of diagnostic accuracy parameters were determined with an univariate random-effects model (DerSimonian Laird method) [69]. Pooled estimates are only shown if heterogeneity (I^2) was <75% for all diagnostic accuracy parameters [70]. Pooling of diagnostic accuracy parameters was performed with MetaDiSc 1.4. Diagnostic accuracy parameters of single studies were determined with StatsDirect 3.2.10. Clin = clinical diagnosis. TAB = temporal artery biopsy. No. ref+ = number of patients with positive reference standard for GCA. 95% CI = 95% confidence interval. PION = posterior ischemic optic neuropathy. RAPD = relative afferent pupillary defect. (-) No pooled estimates reported due to heterogeneity ≥ 75% for at least one diagnostic accuracy parameter.

©2020 American Medical Association. All rights reserved.

eTable 8. Sensitivity Analysis: Distinct Reference Standards

	No. of cohorts (No. of patients)		LR+ (95% CI)		LR- (95% CI)	
	Clinical diagnosis	TAB	Clinical diagnosis	TAB	Clinical Diagnosis	TAB
Age > 60 [5,7,22,28,31,50,64]	4 (164)	4 (126)	1.28 (1.09-1.51)	1.11 (0.96-1.28)	0.17 (0.01-2.02)	0.33 (0.07-1.68)
Age > 70 [5,7,22,28,31,50,64]	4 (164)	4 (126)	1.46 (0.88-2.42)	1.50 (1.07-2.11)	0.66 (0.30-1.46)	0.36 (0.18-0.74)
Anaemia [7,9,12,17,24,28,30,43,50,52,59,61,65,68]	5 (446)	11 (2581)	1.13 (0.91-1.41)	1.21 (1.08-1.37)	0.81 (0.59-1.10)	0.85 (0.75-0.95)
Any TA abnormality [10,19,20,24,27,30,32,33,35,43,50,59,65]	4 (526)	9 (3419)	1.69 (1.20-2.39)	2.48 (1.58-3.87)	0.62 (0.42-0.92)	0.61 (0.45-0.83)
CRP elevated [5,13,15,38,40,42,43,45,47]	5 (548)	5 (1324)	1.34 (1.11-1.61)	1.30 (1.20-1.30)	0.26 (0.08-0.89)	0.42 (0.31-0.56)
Constitutional symptoms [10,15,24,29,40,43,48,62]	5 (563)	4 (935)	1.32 (1.12-1.55)	1.04 (0.76-1.43)	0.60 (0.39-0.93)	0.96 (0.70-1.33)
ESR > 50 mm/h [a] [3,5,7-9,15,16,22,32,41,43,44,49,50,52,60,61,64]	5 (508)	14 (1533)	1.51 (1.25-1.83)	1.39 (1.18-1.64)	0.54 (0.40-0.73)	0.45 (0.32-0.63)
ESR elevated [3,5,7,13,15,18,22,25,26,36,38,45,57,62,68]	4 (310)	11 (3119)	1.10 (0.88-1.36)	1.27 (1.12-1.44)	0.72 (0.36-1.41)	0.51 (0.34-0.76)
Fever [9,12,14,17,19,21,25-27,30,32,33,36,37,40,44,48,50,52,54,59,62,65]	9 (573)	14 (2467)	0.89 (0.66-1.21)*	1.41 (1.11-1.80)*	1.06 (0.90-1.25)	0.91 (0.86-0.96)
Headache [6,9,12,14,15,17,21,23-27,30,32,33,35-37,40,43-45,48,50-56,59,61,63-65,68]	17 (1421)	24 (5930)	1.35 (1.09-1.67)	1.29 (1.17-1.42)	0.61 (0.48-0.77)	0.60 (0.50-0.72)
Jaw claudication [4,9-12,14,15,17,21,23-26,30-33,35-37,40,43,45,48,50-52,54-56,59,61,63,65,68]	19 (1706)	20 (5568)	5.58 (3.44-9.05)	4.37 (3.35-5.71)	0.71 (0.66-0.76)	0.65 (0.62-0.69)
Loss of vision [17,21,23,27,33,35,36,42,48,51,53,54,56,68]	6 (717)	9 (3915)	1.90 (0.56-6.48)	1.41 (1.18-1.69)	0.90 (0.80-1.01)	0.90 (0.86-0.95)
Malaise [14,30,37,50,52,59,61-63,65]	4 (281)	7 (1064)	1.04 (0.86-1.26)	1.18 (0.99-1.42)	0.93 (0.65-1.33)	0.86 (0.75-0.99)
Male [3,4,6-15,21-23,25,27-30,32,33,35,36,40,41,43,44,48-50,52-56,59-61,63,65,68]	21 (1645)	25 (6361)	0.88 (0.76-1.01)	0.90 (0.82-1.00)	1.08 (1.00-1.18)	1.05 (1.00-1.10)
Myalgia [12,14,21,25,30,33,36,37,40,48,52,54,59,62,65]	7 (432)	8 (1372)	0.92 (0.53-1.60)	1.04 (0.90-1.22)	1.06 (0.72-1.56)	0.97 (0.88-1.08)
PMR [1,2,9-11,15,17,19,24,26,27,31,37,39,43-45,50,54,55,61,63,67]	13 (1270)	14 (1952)	1.57 (1.14-2.15)	1.07 (0.89-1.29)	0.83 (0.76-0.90)	0.97 (0.91-1.04)
Scalp tenderness [4,10,17,27,30,32,33,39,40,45,48,52,62,63,68]	7 (726)	7 (2123)	1.69 (1.11-2.56)	1.86 (1.28-2.71)	0.75 (0.66-0.86)	0.81 (0.71-0.92)
TA tenderness [25,31,36,37,42,50,52,54,62,64]	6 (638)	5 (549)	2.20 (0.97-4.98)	1.94 (1.29-2.94)	0.75 (0.64-0.88)	0.84 (0.66-1.08)
Visual disturbance [1,2,4,6,9,11,12,17,19,24,25,32,37,40,42-45,48,50,52,55,59,61,65]	16 (1654)	12 (1729)	1.34 (1.12-1.60)	1.06 (0.89-1.26)	0.88 (0.82-0.95)	0.98 (0.90-1.06)
Weight loss [1,2,11,14,17,21,32,37,40,48,50,52,58,59,61,62,65,68]	9 (646)	10 (2314)	2.03 (1.53-2.70)*	1.48 (1.24-1.77)*	0.74 (0.60-0.91)	0.83 (0.75-0.93)

Likelihood ratios of studies with a clinical diagnosis as the reference standard for GCA were compared to likelihood ratios of studies with TAB as the reference standard. [a] studies reporting an ESR ≥ 50 mm/h were also included. * considered statistically significant, as indicated by a summary estimate difference >0.5 with both estimates outside each other's 95% confidence interval (95% CI). ESR = erythrocyte sedimentation rate. LR+ = positive likelihood ratio. LR- = negative likelihood ratio. PMR = polymyalgia rheumatica. TA = temporal artery. TAB = temporal artery biopsy.

©2020 American Medical Association. All rights reserved.

eTable 9. Sensitivity Analysis: Prospective vs Retrospective Studies

	No. of cohorts (No. of patients)		LR+ (95% CI)		LR- (95% CI)	
	Prospective	Retrospective	Prospective	Retrospective	Prospective	Retrospective
Any TA abnormality [10,19,20,24,27,30,32,33,35,43,50,59,65]	4 (647)	9 (3176)	3.24 (1.00-10.53)	2.08 (1.45-3.00)	0.69 (0.49-0.97)	0.57 (0.43-0.77)
Constitutional symptoms	4 (526)	4 (748)	1.28 (0.97-1.70)	1.11 (0.83-1.48)	0.69 (0.46-1.03)	0.87 (0.49-1.56)
ESR > 50 mm/h [a] [3,5,7-9,15,16,22,32,41,43,44,49,50,52,60,61,64]	4 (198)	14 (1768)	1.70 (1.40-2.20)	1.34 (1.16-1.55)	0.42 (0.27-0.66)	0.50 (0.38-0.65)
ESR elevated [3,5,7,13,15,18,22,25,26,36,38,45,57,62,68]	6 (460)	9 (2969)	1.15 (0.93-1.42)	1.25 (1.11-1.41)	0.68 (0.41-1.11)	0.48 (0.30-0.77)
Fever [9,12,14,17,19,21,25-27,30,32,33,36,37,40,44,48,50,52,54,59,62,65]	7 (916)	16 (2175)	1.21 (0.86-1.71)	1.19 (0.96-1.48)	0.93 (0.84-1.03)	0.95 (0.90-1.00)
Headache [6,9,12,14,15,17,21,23-27,30,32,33,35-37,40,43-45,48,50-56,59,61,63-65,68]	9 (888)	27 (6030)	1.16 (0.88-1.54)	1.36 (1.22-1.52)	0.76 (0.52-1.11)	0.58 (0.50-0.69)
Jaw claudication [4,9-12,14,15,17,21,23-26,30-33,35-37,40,43,45,48,50-52,54-56,59,61,63,65,68]	10 (1053)	25 (5814)	6.99 (2.76-17.74)	4.72 (3.62-6.17)	0.65 (0.59-0.72)	0.68 (0.64-0.72)
Loss of vision [17,21,23,27,33,35,36,42,48,51,53,54,56,68]	4 (628)	10 (3957)	2.34 (0.55-10.04)	1.28 (0.93-1.75)	0.94 (0.84-1.04)	0.94 (0.86-1.01)
Malaise [14,30,37,50,52,59,61-63,65]	4 (780)	6 (487)	1.09 (0.93-1.28)	1.24 (0.96-1.59)	0.88 (0.71-1.10)	0.83 (0.68-1.01)
Male [3,4,6-15,21-23,25,27-30,32,33,35,36,40,41,43,44,48-50,52-56,59-61,63,65,68]	12 (1152)	30 (6646)	0.87 (0.73-1.04)	0.91 (0.83-0.99)	1.08 (0.98-1.18)	1.05 (1.00-1.10)
Myalgia [12,14,21,25,30,33,36,37,40,48,52,54,59,62,65]	7 (916)	8 (939)	0.74 (0.54-1.03)	1.10 (0.90-1.40)	1.25 (0.90-1.73)	0.92 (0.79-1.06)
PMR [1,2,9-11,15,17,19,24,26,27,31,37,39,43-45,50,54,55,61,63,67]	7 (555)	16 (2259)	1.14 (0.80-1.62)	1.34 (1.08-1.67)	0.93 (0.77-1.12)	0.90 (0.85-0.96)
Scalp tenderness [4,10,17,27,30,32,33,39,40,45,48,52,62,63,68]	8 (1111)	7 (1840)	1.42 (1.06-1.91)*	2.29 (1.60-3.29)*	0.83 (0.75-0.92)	0.75 (0.64-0.88)
TA tenderness [25,31,36,37,42,50,52,54,62,64]	4 (718)	6 (418)	1.71 (0.91-3.22)	2.30 (1.47-3.59)	0.80 (0.67-0.95)	0.76 (0.55-1.05)
TA thickening [1,2,11,12,42,50,52,61]	4 (596)	4 (333)	2.90 (2.00-4.30)	7.52 (2.21-25.57)	0.75 (0.69-0.82)	0.43 (0.28-0.68)
Transient vision loss [17,21,33,46,48,58,62,63,67]	4 (531)	5 (650)	3.72 (0.28-49.40)	1.53 (0.90-2.59)	0.95 (0.89-1.00)	0.94 (0.87-1.02)
Visual disturbance [1,2,4,6,9,11,12,17,19,24,25,32,37,40,42-45,48,50,52,55,59,61,65]	8 (803)	17 (2220)	1.27 (0.95-1.71)	1.19 (0.99-1.42)	0.91 (0.81-1.02)	0.92 (0.85-1.00)
Weight loss [1,2,11,14,17,21,32,37,40,48,50,52,58,59,61,62,65,68]	6 (582)	12 (2300)	2.21 (1.70-2.89)*	1.48 (1.26-1.73)*	0.67 (0.53-0.85)	0.85 (0.77-0.94)

Likelihood ratios of prospective studies were compared to likelihood ratios of retrospective studies. [a] studies reporting an ESR ≥ 50 mm/h were also included. * considered statistically significant, as indicated by a summary estimate difference >0.5 with both estimates outside each other's 95% confidence interval (95% CI). ESR = erythrocyte sedimentation rate. LR+ = positive likelihood ratio. LR- = negative likelihood ratio. PMR = polymyalgia rheumatica. TA = temporal artery.

©2020 American Medical Association. All rights reserved.

eTable 10. Sensitivity Analysis: Pretreatment Laboratory Tests

Finding	No. of cohorts (No. of patients)	Sensitivity (95% CI)	Specificity (95% CI)	LR+ (95% CI)	LR- (95% CI)
CRP elevated	4 (477)	90.1 (76.3-96.3)	26.3 (12.7-46.8)	1.22 (1.01-1.49)	0.38 (0.17-0.81)
ESR > 50 mm/h	4 (396)	87.5 (78.3-93.1)	46.5 (30.3-63.6)	1.64 (1.15-2.33)	0.27 (0.13-0.57)

The diagnostic accuracy of an elevated CRP level and ESR > 50 mm/h as indicated by studies reporting pre-treatment laboratory values. A meta-analysis could not be performed for other laboratory features due to insufficient studies (i.e. less than four studies). An elevated CRP level was defined as ≥0.5 mg/dL unless other lab-specific normal values were reported. 95% CI = 95% confidence interval. CRP = C-reactive protein. ESR = erythrocyte sedimentation rate. LR+ = positive likelihood ratio. LR- = negative likelihood ratio.

©2020 American Medical Association. All rights reserved.

eAppendix. Standard Form for Study Characteristics and QUADAS-2 Items

Name of study (first author + year):……………………………………………RETROSPECTIVE / PROSPECTIVE
Centres (city): ……………..
Recruitment period: …………………………………………….

<u>Setting and patient selection</u>

1. Setting:
    a. Non-academic hospital / Academic hospital / unclear / other ……………………..
    b. Specialty department where patients were identified
        i. One clinical specialty, i.e. ……………………………………………………
        ii. Multiple clinical specialties, i.e.:……………………………………………………………
        iii. Central pathology / surgery registry of hospital
        iv. Other:……………………………………………
    c. Patients referred by: unclear/primary/rheum/internal/ophthalm /other:……….……………………
2. Included patients:
    a. Patients referred to clinic for suspicion of GCA
    b. Patients undergoing TAB
    c. Patients undergoing imaging: US / CTA / MRA / PET-CT / PET
    d. Other: ……………………………………………..
3. Risk of bias:
    a. Consecutive or random sample of patients was enrolled: YES / NO / UNCLEAR
    b. A case control design was avoided: YES / NO / UNCLEAR
    c. The study avoided inappropriate exclusions: YES / NO / UNCLEAR; number excluded patients…………………………
4. Could the selection of patients have introduced bias: HIGH RISK / LOW RISK / UNCLEAR
5. Concern that included patients do not match the review question: HIGH / LOW / UNCLEAR

<u>Index tests</u>

1. Were the index tests interpreted without knowledge of results of reference standard: YES / NO / UNCLEAR
2. Was a pre-specified threshold used for age: NOT APPLICABLE / YES / NO / UNCLEAR
3. Was a pre-specified threshold used for lab tests: NOT APPLICABLE / YES / NO / UNCLEAR
4. Could the conduct or interpretation of the index have introduced bias: HIGH RISK / LOW RISK / UNCLEAR
5. Concern that the index test, its conduct/interpretation differ from review question: HIGH RISK / LOW RISK / UNCLEAR
6. Lab results reported for not (yet) treated patients: NOT APPLICABLE / YES / NO / UNCLEAR
7. Missing data for laboratory tests < 25%: NOT APPLICABLE / APPLICABLE
    a. Test name(s): ………………………………………………………………YES / NO / UNCLEAR
    b. Test name(s): ………………………………………………………………YES / NO / UNCLEAR

<u>Reference standard</u>

1. Is reference standard likely to classify condition correctly: YES / NO / UNCLEAR
    a. TAB →…………………………………….. patients bilateral TAB; TAB length: ………………………
    b. Imaging: US / CTA / MRA / PET-CT / PET
    c. Clinical diagnosis with FOLLOW-UP: NO / UNCLEAR / ≥ 3 MONTHS / ≥ 6 MONTHS
        i. Every patient also TAB / US / CTA / MRA / PET-CT / PET
2. Reference standard focused on: CRANIAL GCA / SYSTEMIC GCA / CRANIAL+SYSTEMIC / UNCLEAR
3. Was the reference standard interpreted without knowledge of the index test: YES / NO / UNCLEAR
4. Reference standard, its conduct/interpretation could have introduced bias: HIGH RISK / LOW RISK / UNCLEAR
5. Concern that target condition defined by reference standard does not match review question: HIGH / LOW / UNCLEAR

<u>Flow and timing:</u>

1. Was there appropriate interval between index test and reference standard: YES / NO / UNCLEAR
2. If clinical diagnosis GCA with follow-up, did non-GCA patients receive follow-up: NOT APPLICABLE / YES / NO / UNCLEAR
3. Did all patients receive the reference standard: YES / NO / UNCLEAR
4. Did patients receive the same reference standard: YES / NO / UNCLEAR
5. Were all patients included in the analysis: YES / NO / UNCLEAR; if no: ……………………………….patients lost during study
6. Could the patient flow have introduced bias: HIGH RISK / LOW RISK / UNCLEAR

©2020 American Medical Association. All rights reserved.

eFigure 1. PRISMA Flow Diagram

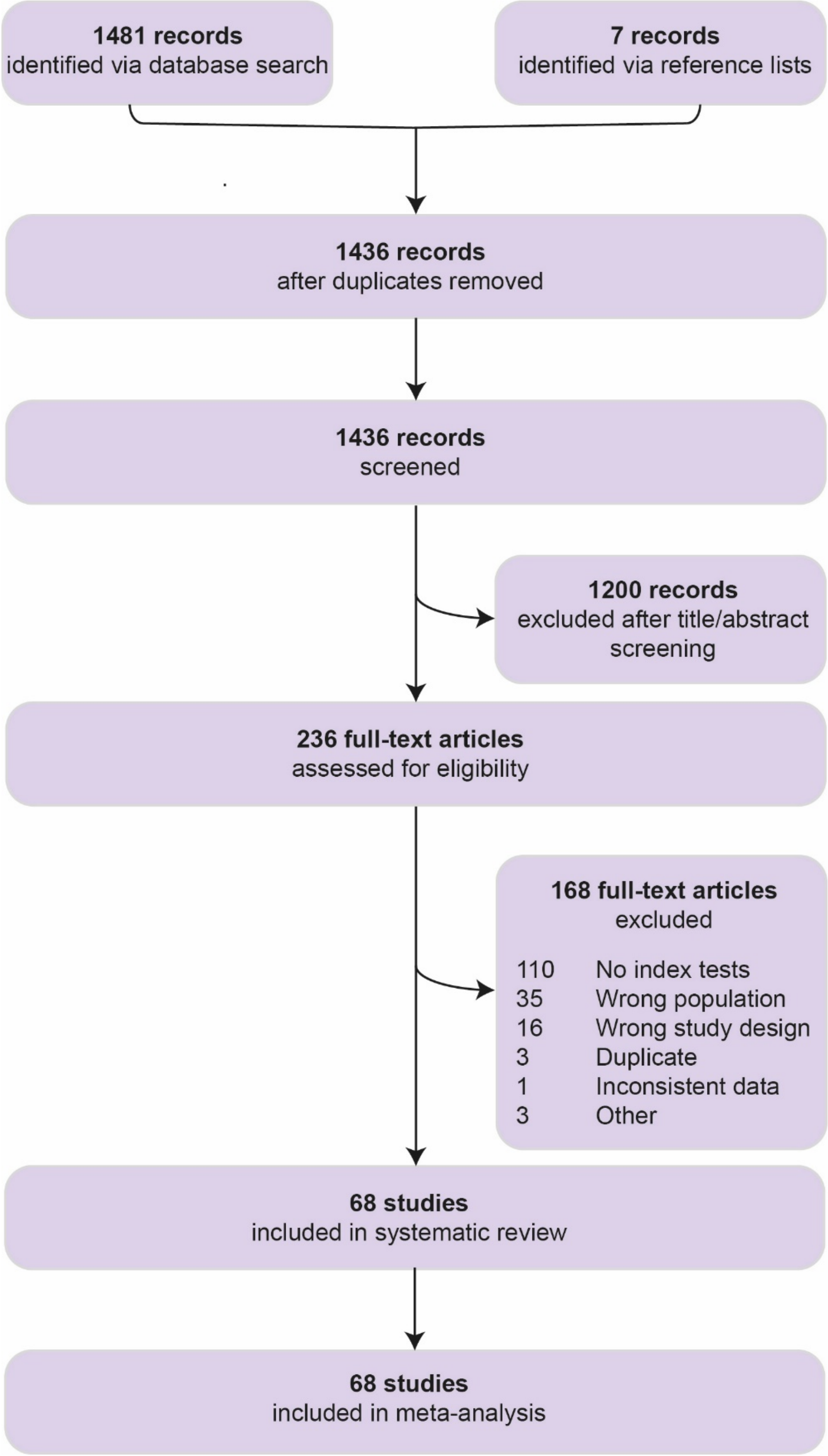

©2020 American Medical Association. All rights reserved.

eFigure 2. Overall Summary of QUADAS-2 Items

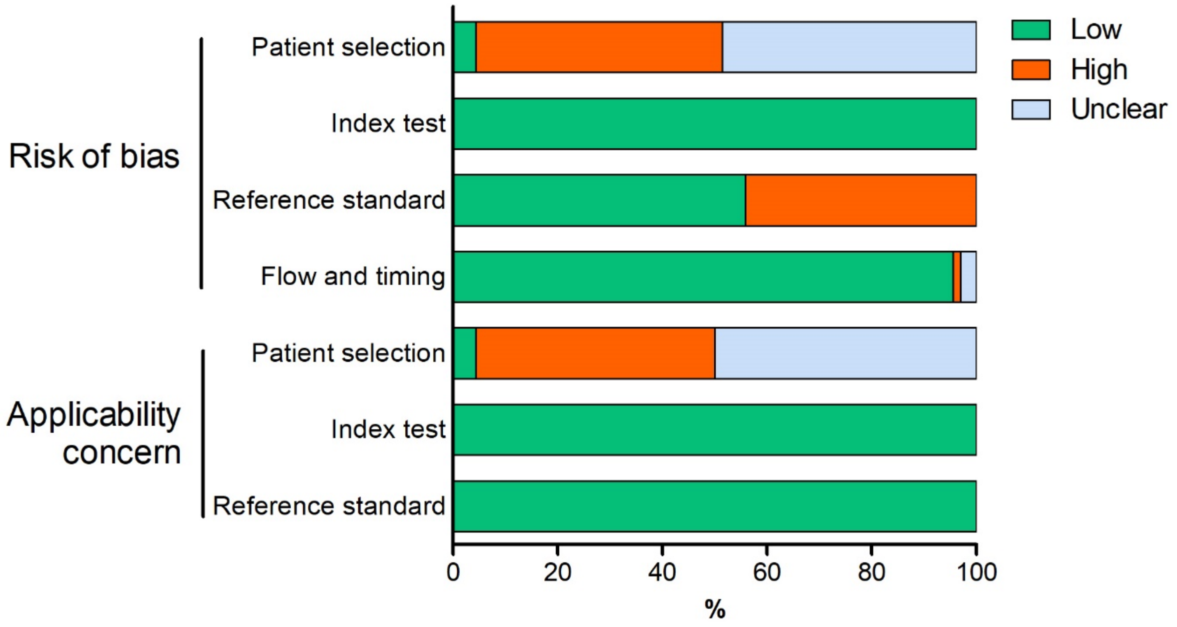

Risk of bias and concern of applicability was evaluated for the 68 studies included in the systematic review and meta-analysis.

©2020 American Medical Association. All rights reserved.

eFigure 3. Detailed Summary of QUADAS-2 Items

	RISK OF BIAS				APPLICABILITY CONCERNS		
	Patient selection	Index test	Reference standard	Flow and timing	Patient selection	Index test	Reference standard
Hop et al. 2020	☹	☺	☹	☺	☹	☺	☺
Imfeld et al. 2020	?	☺	☹	☺	?	☺	☺
van der Geest et al. 2020	?	☺	☹	☺	?	☺	☺
Mukhtyar et al. 2020	?	☺	☹	☺	?	☺	☺
Sommer et al. 2019	☹	☺	☺	☺	☹	☺	☺
Sundholm et al. 2019	?	☺	☹	☺	?	☺	☺
Nielsen et al. 2019	☹	☺	☹	☺	☹	☺	☺
Sammel et al. 2019	☹	☺	☹	☺	☹	☺	☺
Gospe et al. 2019	☹	☺	☺	☺	☹	☺	☺
Ing et al. 2019	?	☺	☺	☺	?	☺	☺
Oiwa et al. 2019	?	☺	☹	☺	?	☺	☺
Conway et al. 2019	☹	☺	☹	☺	☹	☺	☺
Chan et al. 2019	☹	☺	☹	☺	☹	☺	☺
Hay et al. 2019	☹	☺	☹	☺	☹	☺	☺
Oh et al. 2018	☹	☺	☺	☺	☹	☺	☺
Ing et al. 2018	☹	☺	☺	☺	☹	☺	☺
Bilyk et al. 2018	☹	☺	☺	☺	☹	☺	☺
Monti et al. 2017	☺	☺	☹	☹	☺	☺	☺
Czihal et al. 2017	?	☺	☹	☺	?	☺	☺
Roncato et al. 2017	☹	☺	☹	☺	☹	☺	☺
Toren et al. 2016	?	☺	☺	☺	?	☺	☺
Grossman et al. 2016	?	☺	☹	☺	?	☺	☺
Luqmani et al. 2016	☹	☺	☹	?	?	☺	☺
Lariviere et al. 2016	?	☺	☹	?	?	☺	☺
De Lott et al. 2015	☹	☺	☺	☺	☹	☺	☺
Stacy et al. 2015	☹	☺	☺	☺	☹	☺	☺
El-Dairi et al. 2015	?	☺	☺	☺	?	☺	☺
Croft et al. 2015	☹	☺	☹	☺	☹	☺	☺
Aschwanden et al. 2015	☺	☺	☹	☺	☺	☺	☺
Knecht et al. 2015	☹	☺	☺	☺	☹	☺	☺
Diamantopoulos et al. 2014	?	☺	☹	☺	?	☺	☺
Gonzalez-Lopez et al. 2013	?	☺	☺	☺	?	☺	☺
Aschwanden et al. 2013	?	☺	☹	☺	?	☺	☺
Black et al. 2013	?	☺	☹	☺	?	☺	☺
Suelves et al. 2013	?	☺	☺	☺	?	☺	☺
Kermani et al. 2012	☹	☺	☺	☺	☹	☺	☺

©2020 American Medical Association. All rights reserved.

Quinn et al. 2012	☹	☺	☺	☺	☹	☺	☺
Habib et al. 2012	?	☺	☹	☺	?	☺	☺
Lugo et al. 2011	?	☺	☺	☺	?	☺	☺
Walvick et al. 2011	?	☺	☺	☺	?	☺	☺
Mari et al. 2009	?	☺	☺	☺	?	☺	☺
Ghinoi et al. 2008	☺	☺	☺	☺	☺	☺	☺
Moutray et al. 2008	☹	☺	☺	☺	☹	☺	☺
Bley et al. 2008	☹	☺	☹	☺	☹	☺	☺
Hautzel et al. 2008	☹	☺	☹	☺	☹	☺	☺
Rodriguez-Pla et al. 2007	?	☺	☺	☺	?	☺	☺
Karahaliou et al. 2006	?	☺	☹	☺	?	☺	☺
Bley et al. 2005	?	☺	☹	☺	?	☺	☺
Younge et al. 2004	☹	☺	☺	☺	☹	☺	☺
Varma et al. 2004	?	☺	☺	☺	?	☺	☺
Hall et al. 2003	?	☺	☺	☺	?	☺	☺
Foroozan et al. 2002	☹	☺	☺	☺	☹	☺	☺
Mohamed et al. 2002	?	☺	☺	☺	?	☺	☺
Grosser et al. 1999	☹	☺	☺	☺	☹	☺	☺
Hayreh et al. 1997	☹	☺	☺	☺	☹	☺	☺
Gabriel et al. 1995	?	☺	☺	☺	?	☺	☺
Skaug et al. 1995	☹	☺	☹	☺	☹	☺	☺
Chmelewski et al. 1992	?	☺	☺	☺	?	☺	☺
Brittain et al. 1991	☹	☺	☺	☺	☹	☺	☺
Kent et al. 1989	?	☺	☺	☺	?	☺	☺
Stuart 1989	?	☺	☺	☺	?	☺	☺
Fernandez-Herlihy 1988	?	☺	☺	☺	?	☺	☺
Wells et al. 1989	☹	☺	☺	☺	☹	☺	☺
Vilaseca 1987	?	☺	☺	☺	?	☺	☺
Roth et al. 1984	☹	☺	☹	☺	☹	☺	☺
Hall et al. 1983	?	☺	☺	☺	?	☺	☺
Hedges et al. 1983	☹	☺	☹	☺	☹	☺	☺
Eshagian et al. 1980	☹	☺	☺	☺	☹	☺	☺

☺	☹	?
Low risk	High risk	Unclear

Risk of bias and concern of applicability was evaluated for the 68 studies included in the systematic review and meta-analysis [1-68].

©2020 American Medical Association. All rights reserved.

eFigure 4. Forest Plots

Age > 60 (Sensitivity)

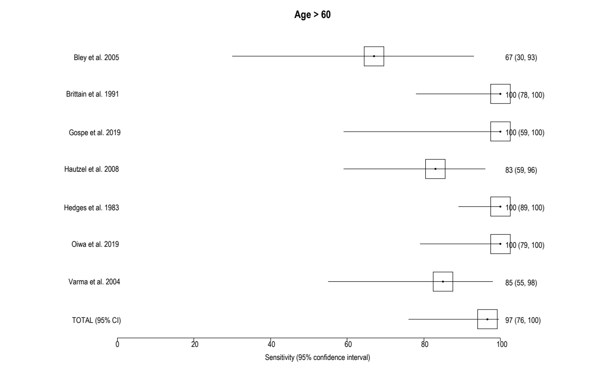

Age > 60 (Specificity)

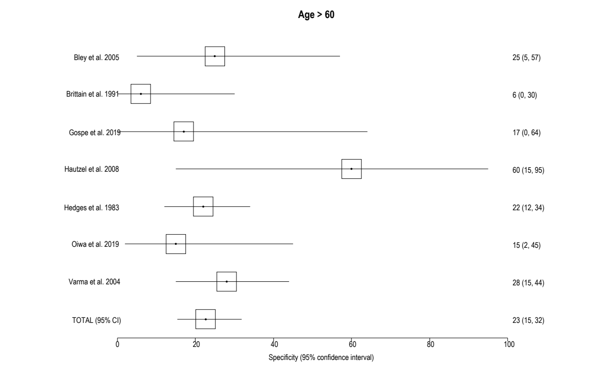

Age > 70 (Sensitivity)

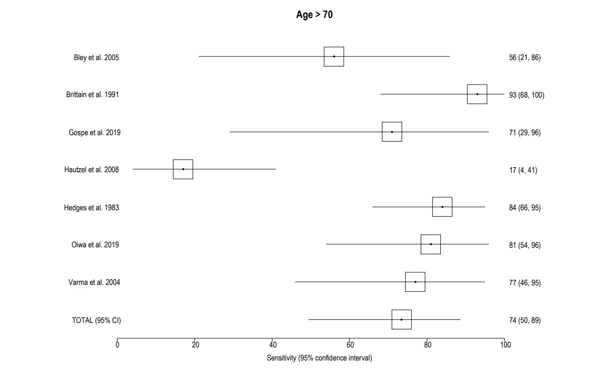

Age > 70 (Specificity

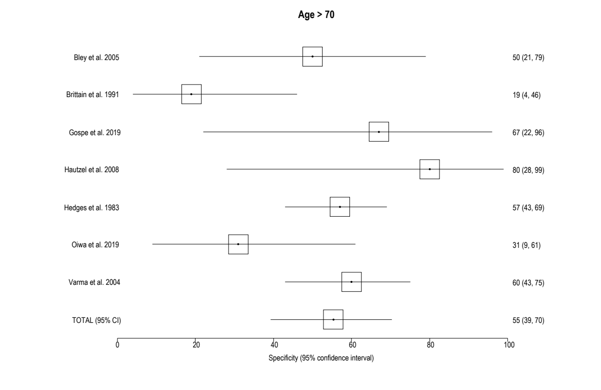

Age > 80 (Sensitivity)

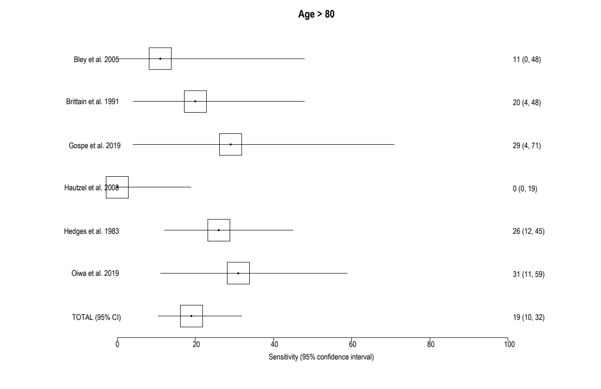

Age > 80 (Specificity)

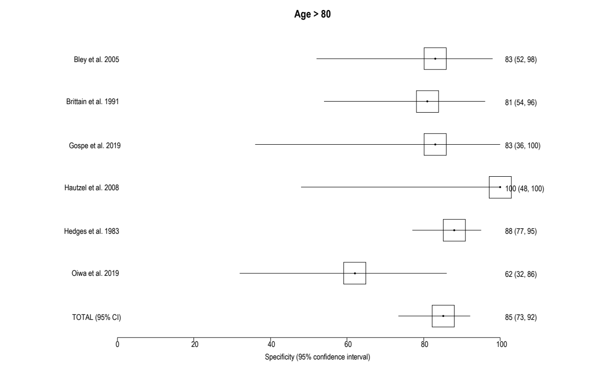

AION (Sensitivity)

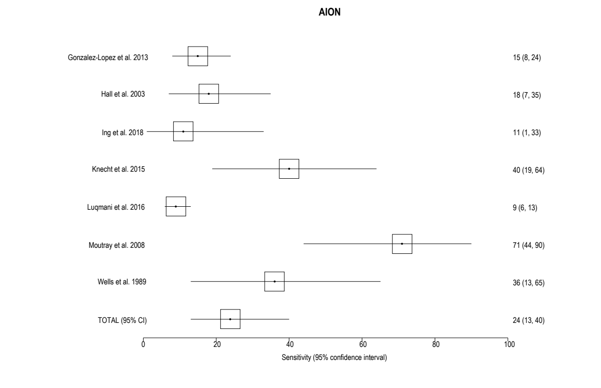

AION (Specificity)

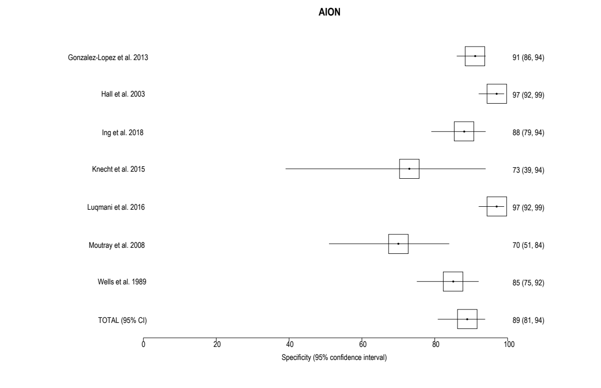

©2020 American Medical Association. All rights reserved.

Anaemia (Sensitivity)

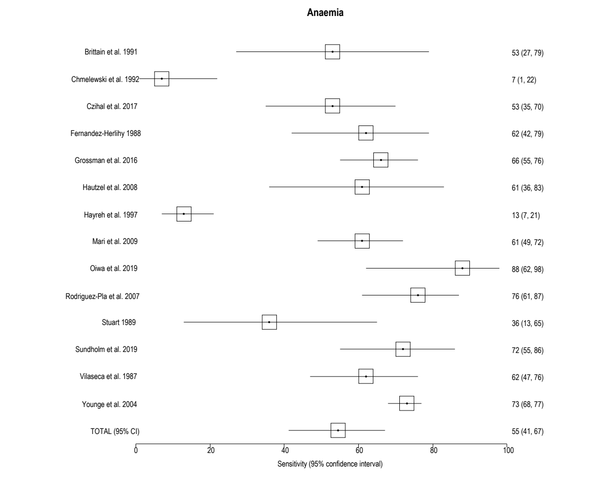

Anaemia (Specificity)

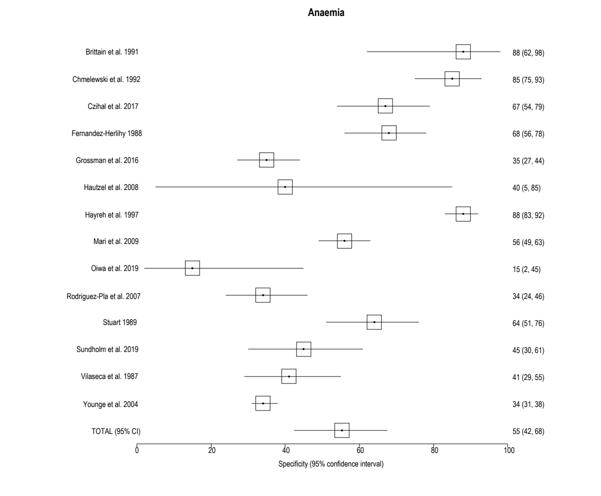

Anorexia (Sensitivity)

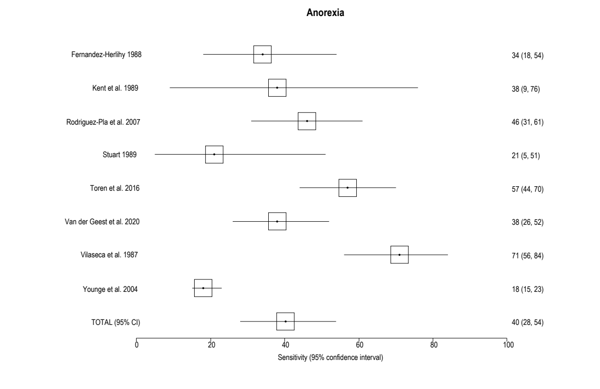

Anorexia (Specificity)

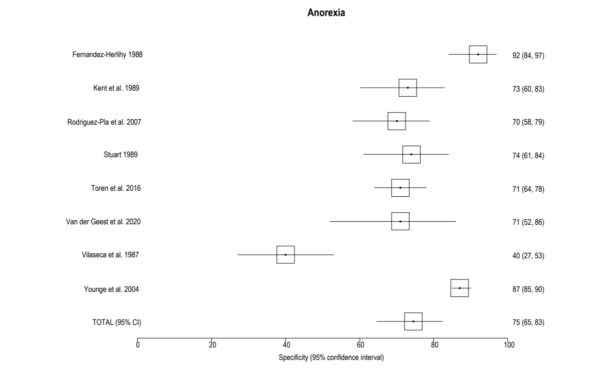

Any TA abnormality (Sensitivity)

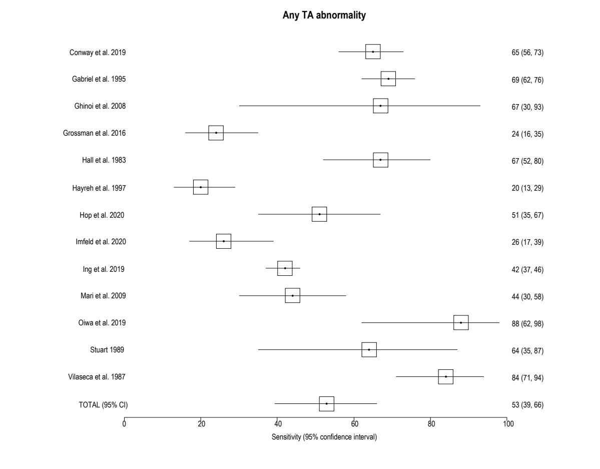

Any TA abnormality (Specificity)

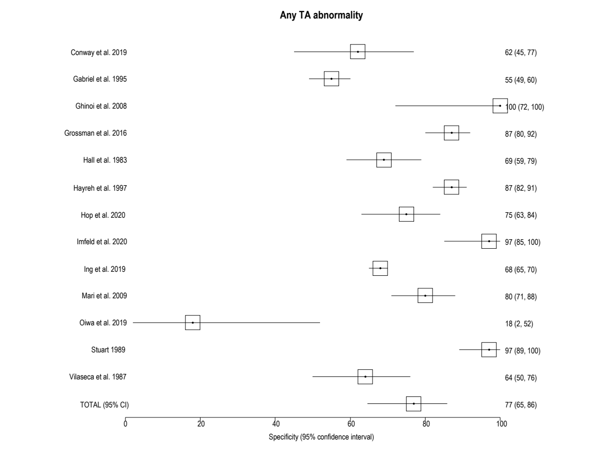

©2020 American Medical Association. All rights reserved.

Arthralgia (Sensitivity)

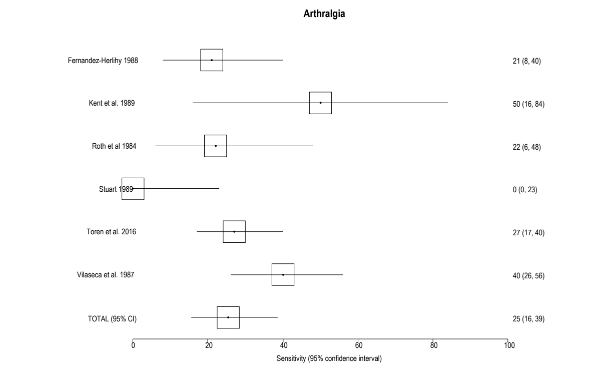

Arthralgia (Specificity)

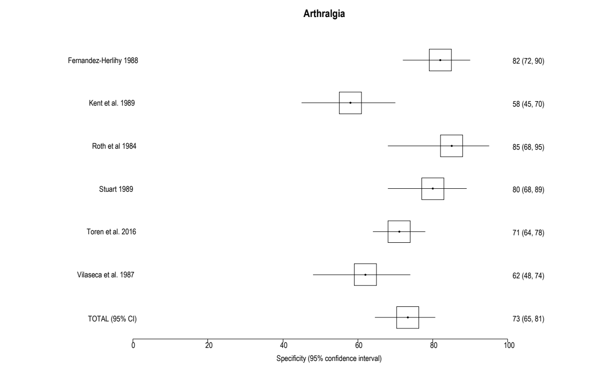

Cerebrovascular accident (Sensitivity)

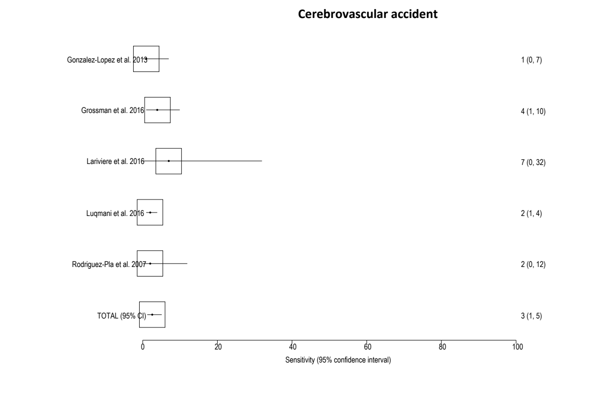

Cerebrovascular accident (Specificity)

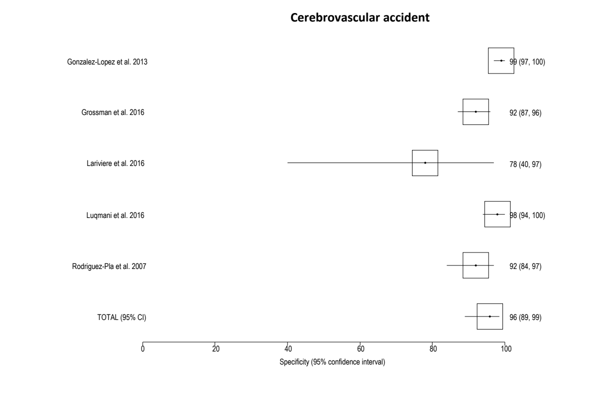

Constitutional symptoms (Sensitivity)

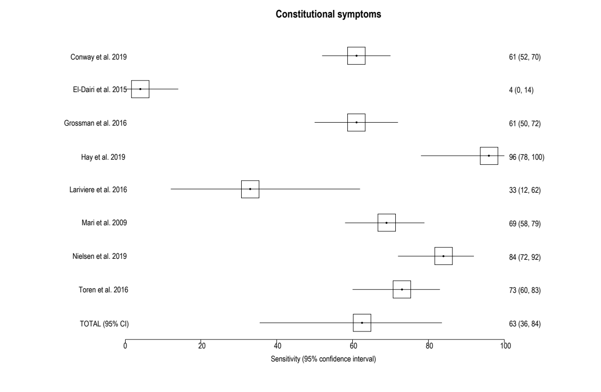

Constitutional symptoms (Specificity)

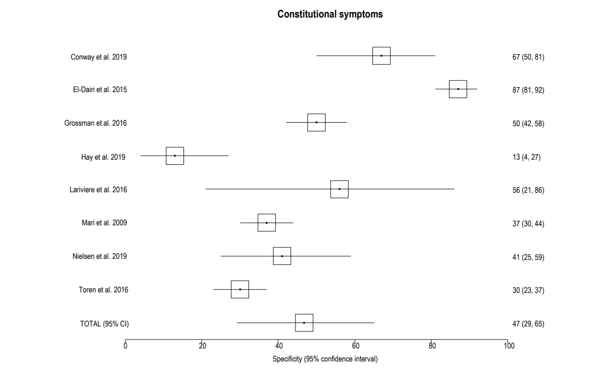

CRAO (Sensitivity)

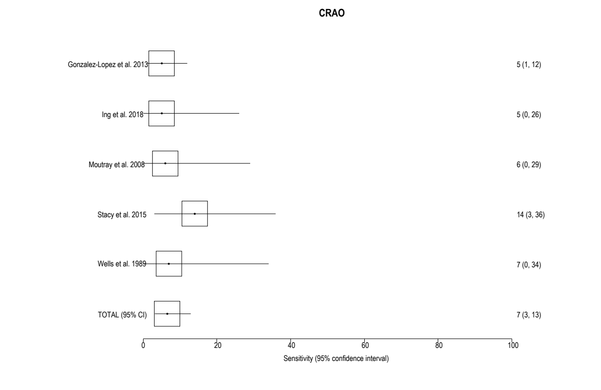

CRAO (Specificity)

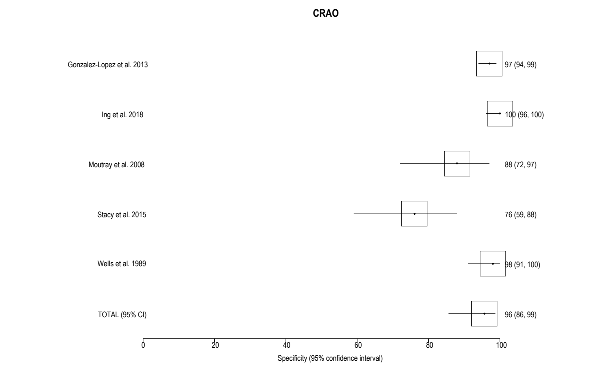

©2020 American Medical Association. All rights reserved.

CRP elevated (Sensitivity)

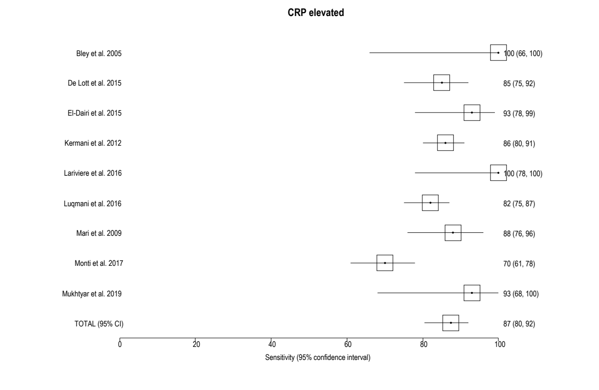

CRP elevated (Specificity)

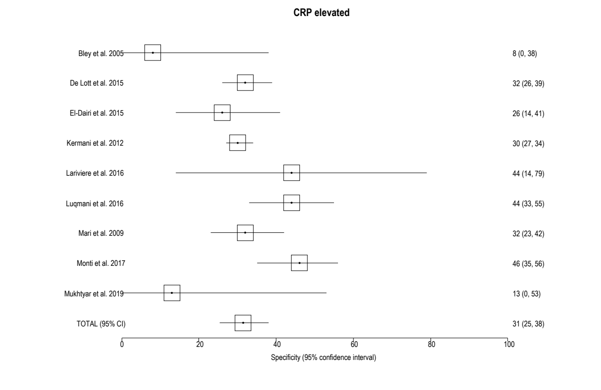

CRP ≥ 2.5 mg/dL (Sensitivity)

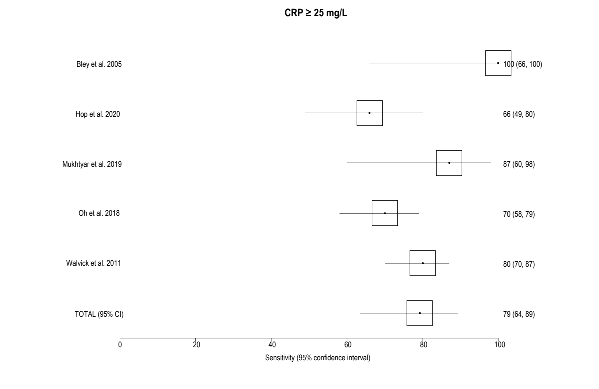

CRP ≥ 2.5 mg/dL (Specificity)

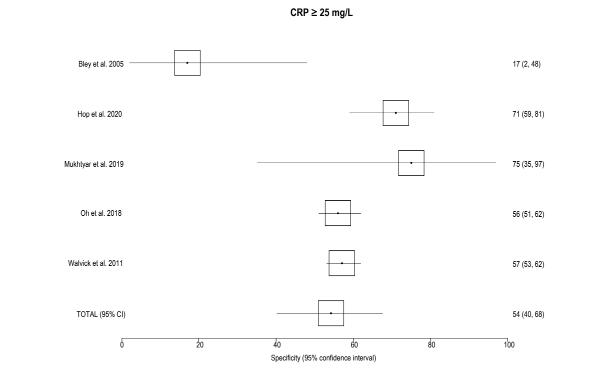

Double vision (Sensitivity)

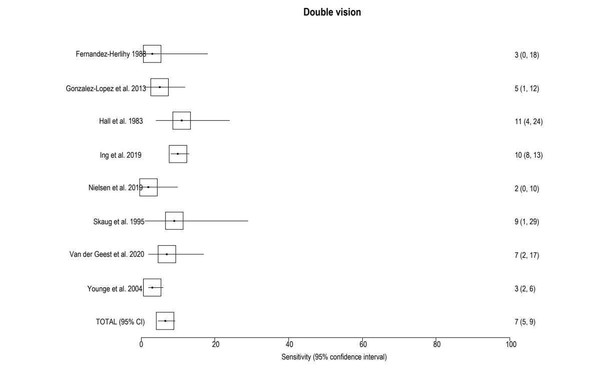

Double vision (Specificity)

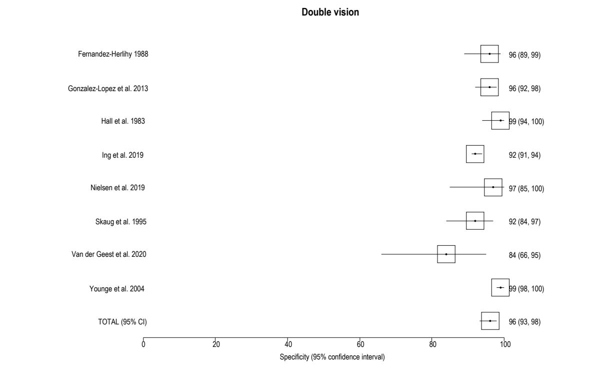

ESR elevated (Sensitivity)

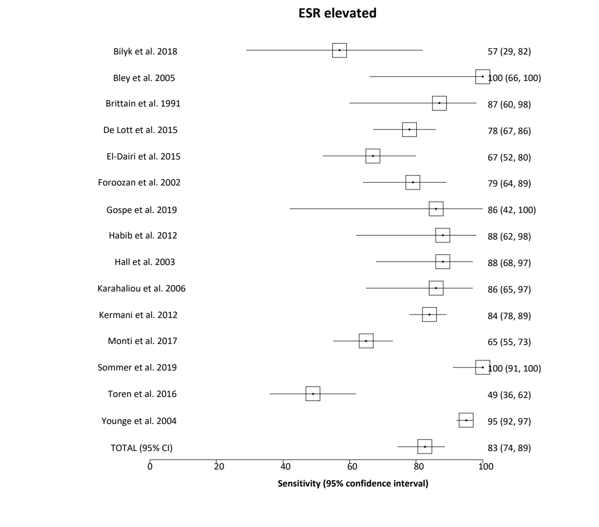

ESR elevated (Specificity)

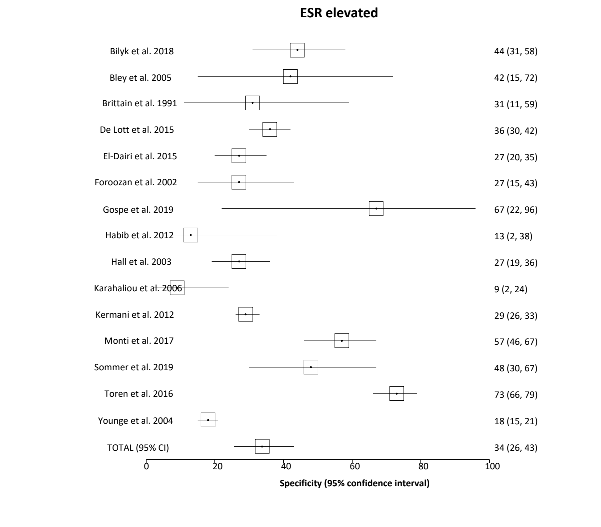

©2020 American Medical Association. All rights reserved.

ESR > 40 mm/h (Sensitivity)

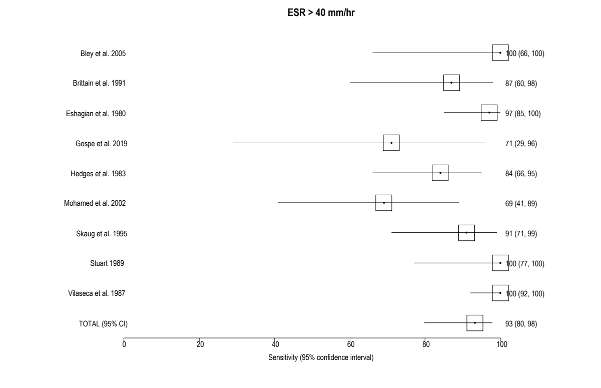

ESR > 40 mm/h (Specificity)

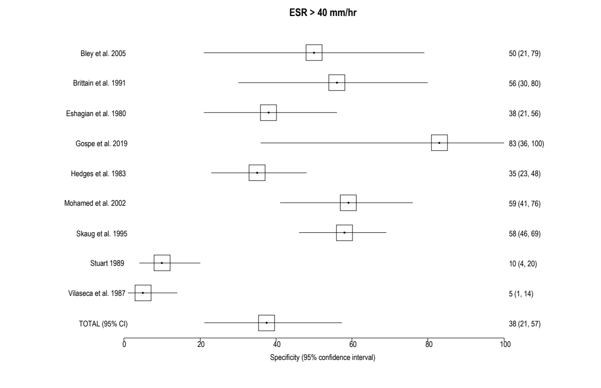

ESR > 50 mm/h (Sensitivity) [a]

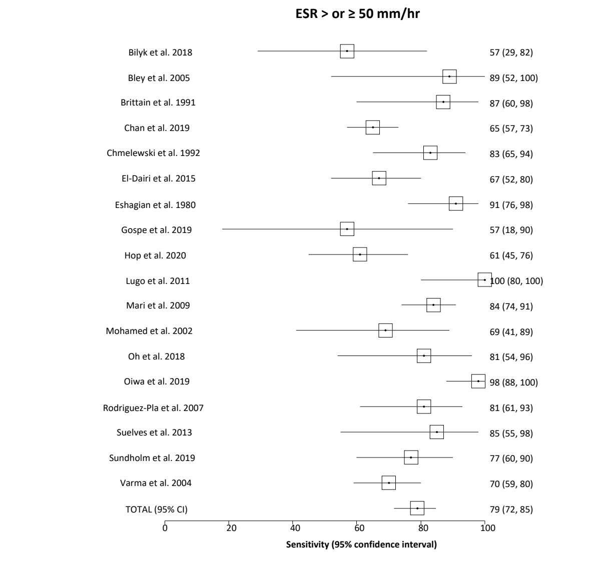

ESR > 50 mm/h (Specificity) [a]

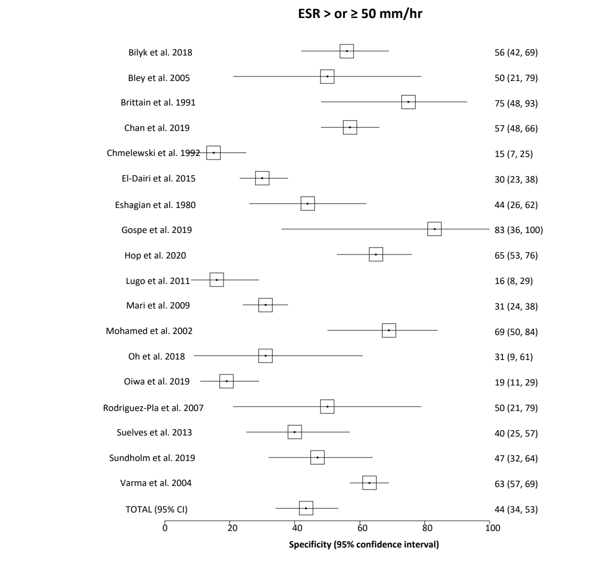

ESR > 60 mm/h (Sensitivity)

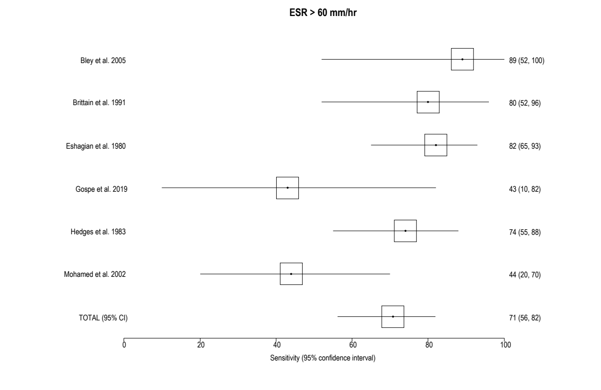

ESR > 60 mm/h (Specificity)

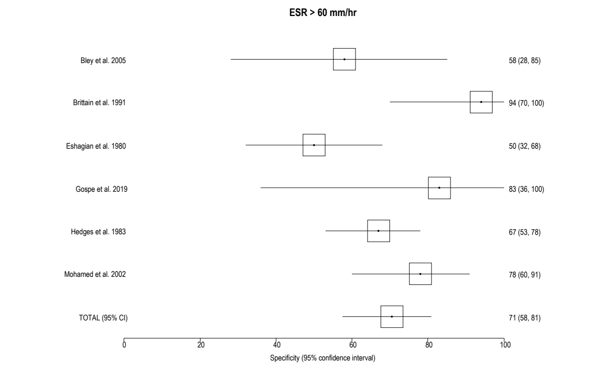

©2020 American Medical Association. All rights reserved.

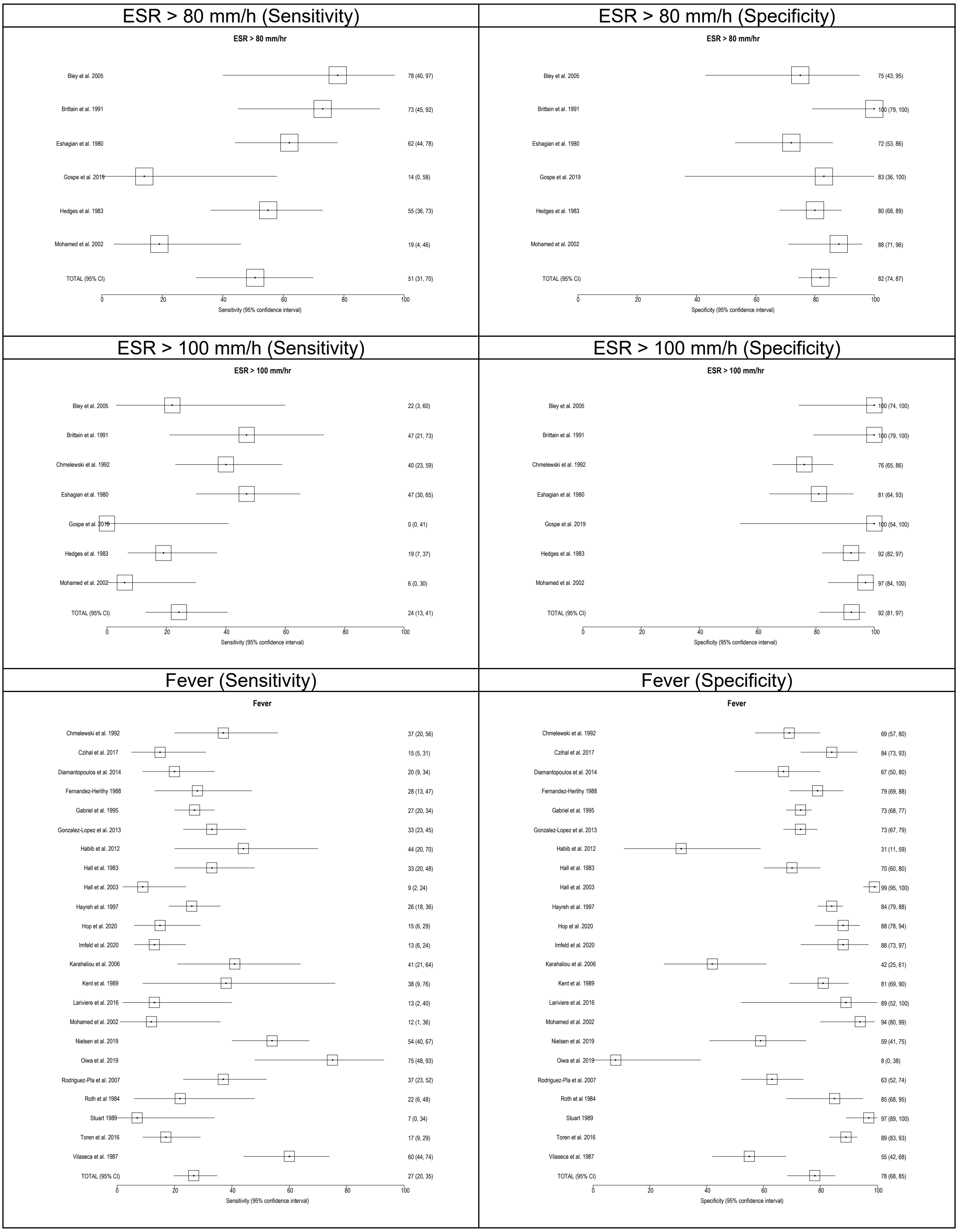

©2020 American Medical Association. All rights reserved.

Headache (Sensitivity)

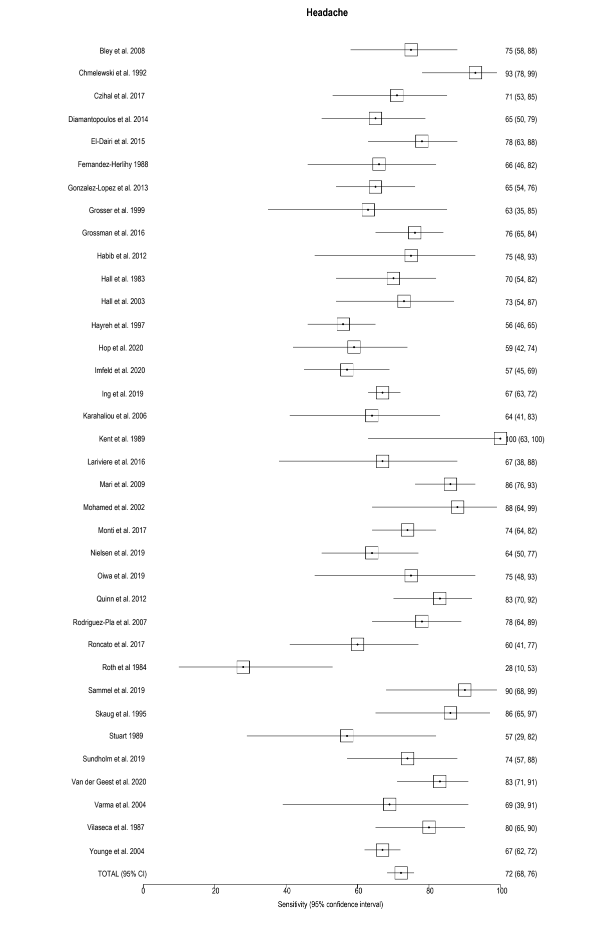

Headache (Specificity)

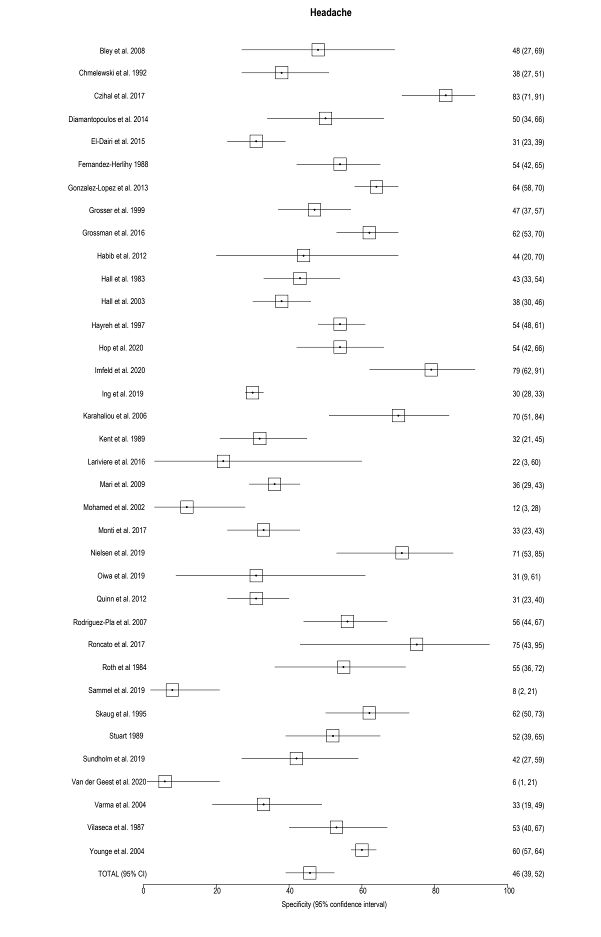

Ischemic optic neuropathy (Sensitivity)

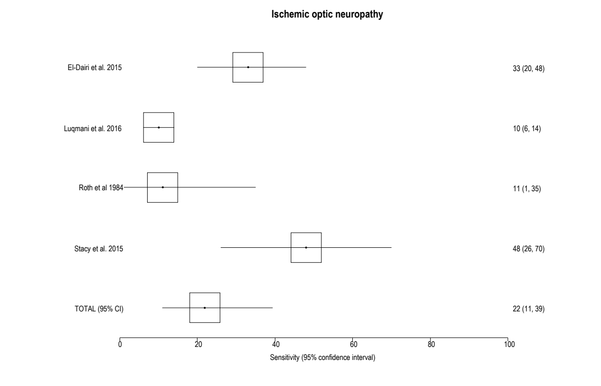

Ischemic optic neuropathy (Specificity)

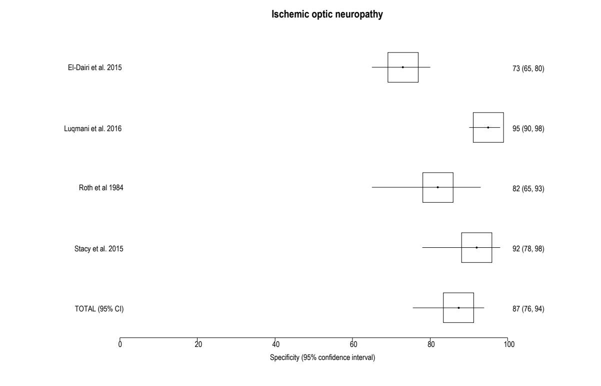

©2020 American Medical Association. All rights reserved.

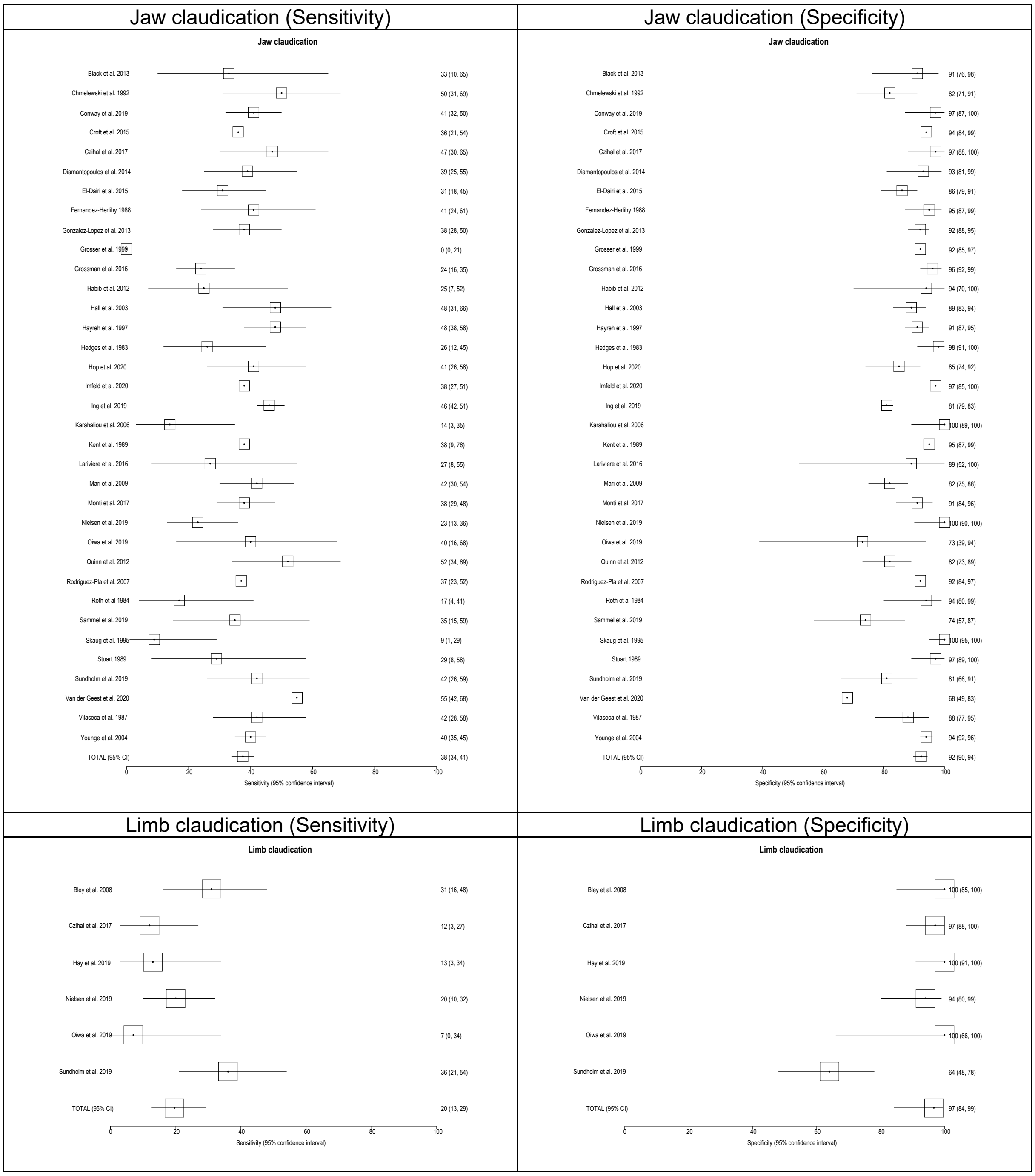

©2020 American Medical Association. All rights reserved.

Loss of vision (Sensitivity)

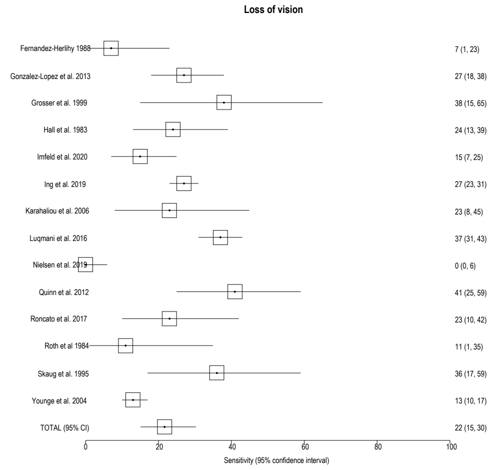

Loss of vision (Specificity)

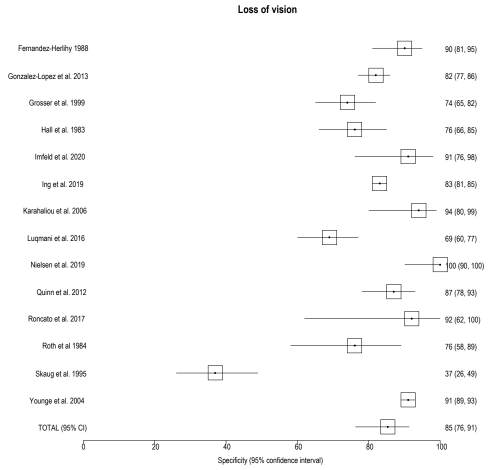

Malaise (Sensitivity)

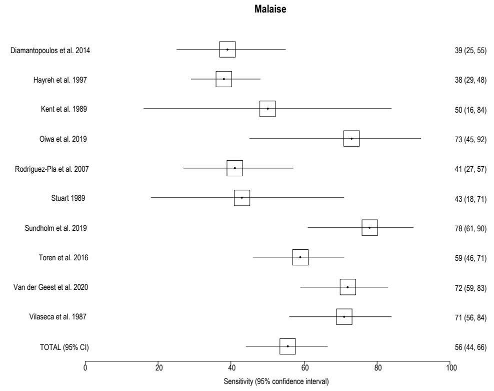

Malaise (Specificity)

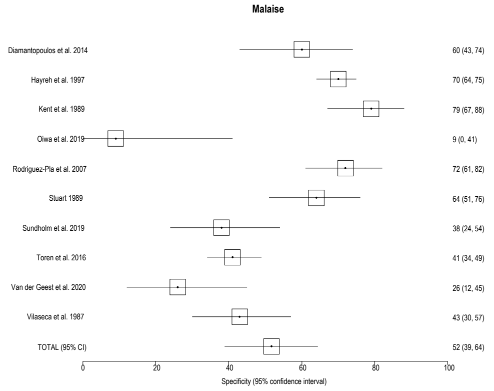

©2020 American Medical Association. All rights reserved.

Male (Sensitivity)

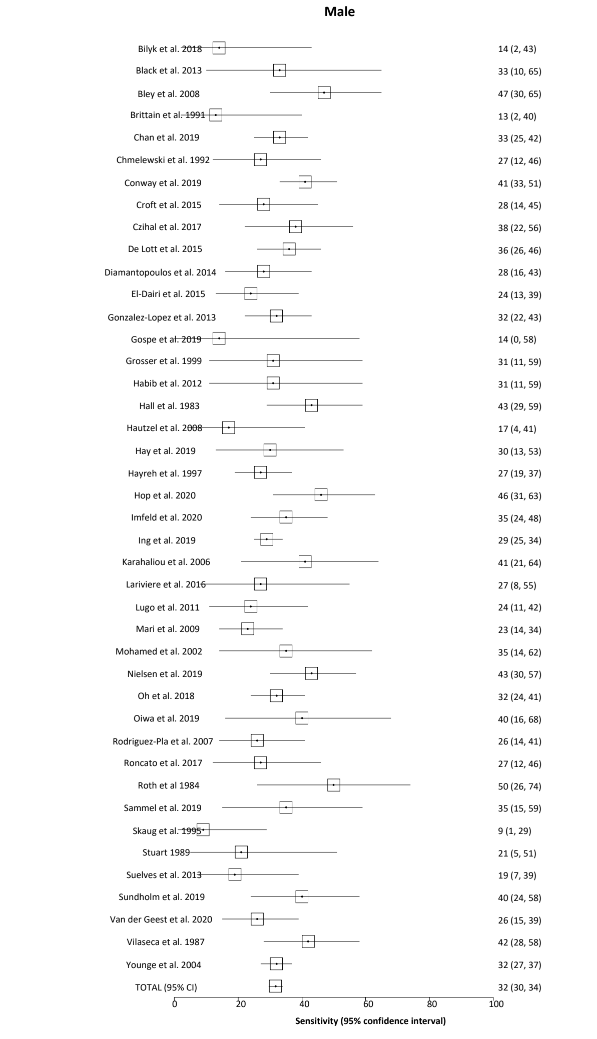

Male (Specificity)

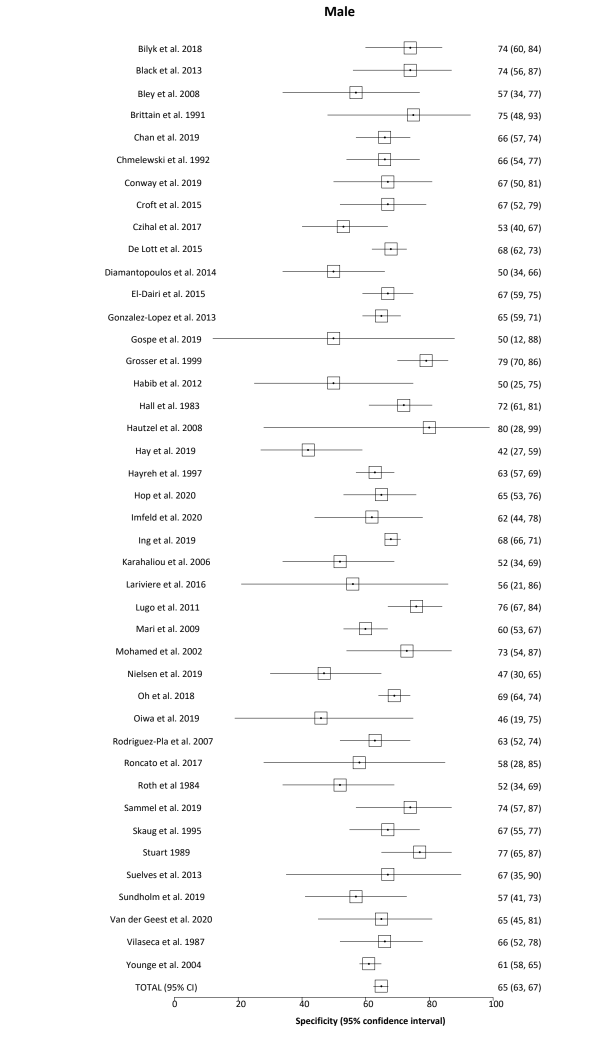

Myalgia (Sensitivity)

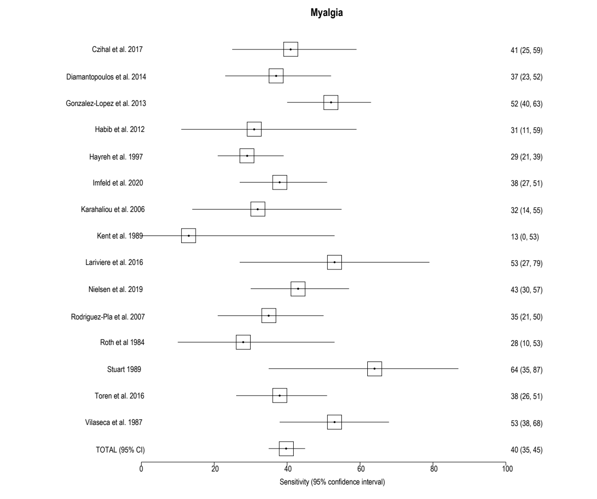

Myalgia (Specificity)

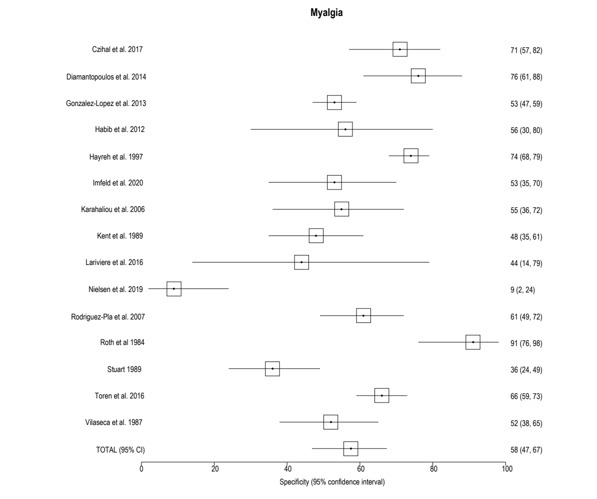

©2020 American Medical Association. All rights reserved.

Platelets > 400 *10^3/μL (Sensitivity) [a]

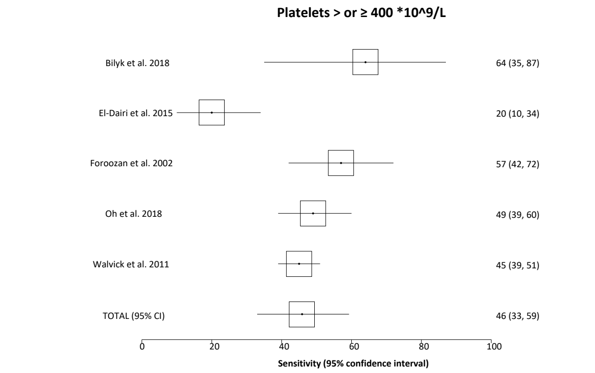

Platelets > 400 *10^3/μL (Specificity) [a]

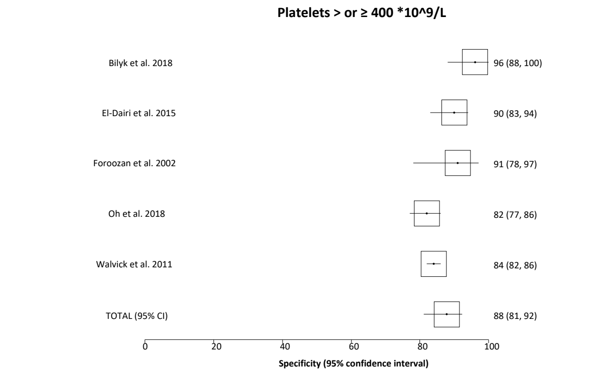

PMR (Sensitivity)

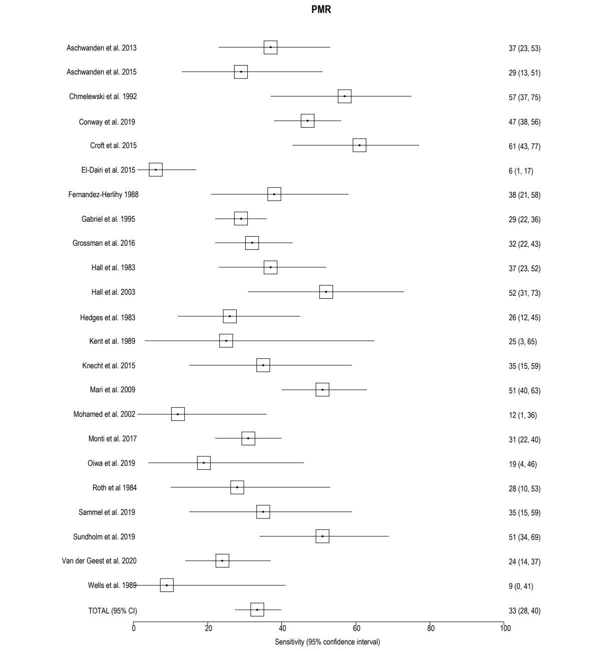

PMR (Specificity)

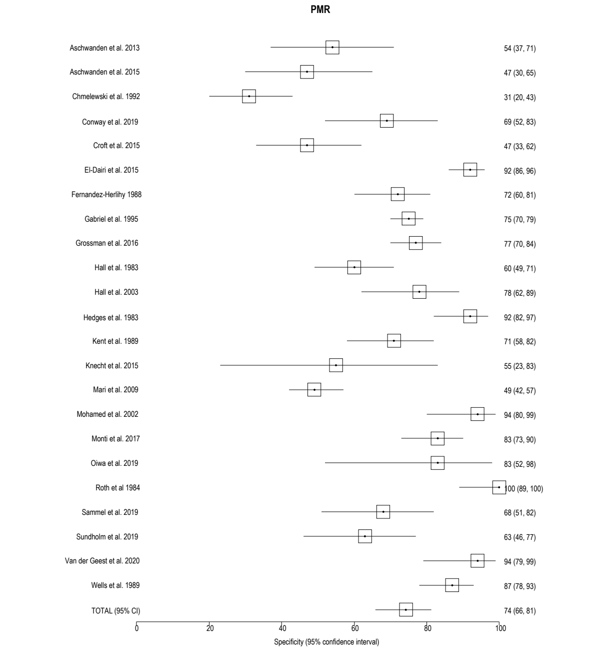

Previous PMR (Sensitivity)

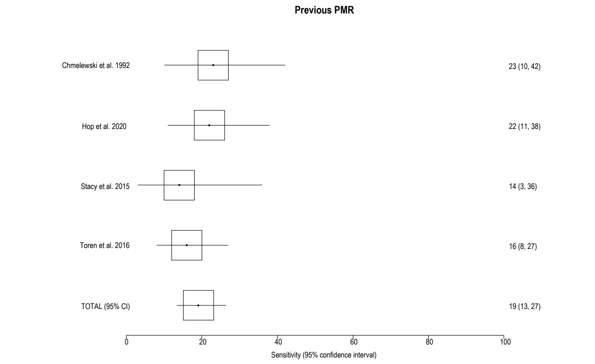

Previous PMR (Specificity)

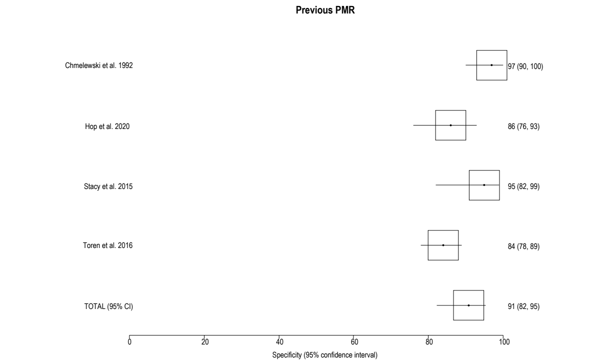

©2020 American Medical Association. All rights reserved.

Scalp tenderness (Sensitivity)

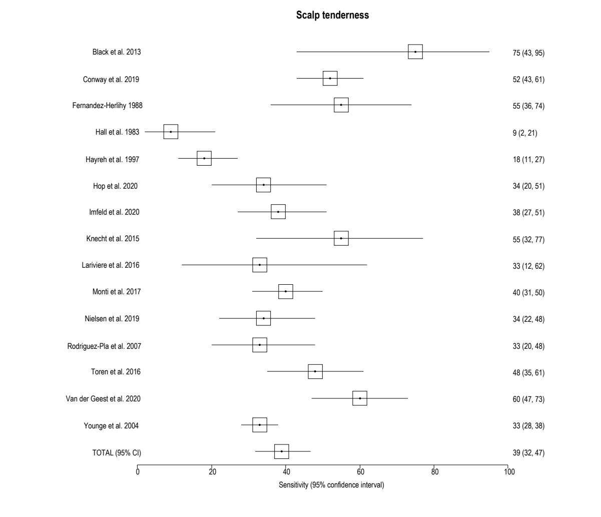

Scalp tenderness (Specificity)

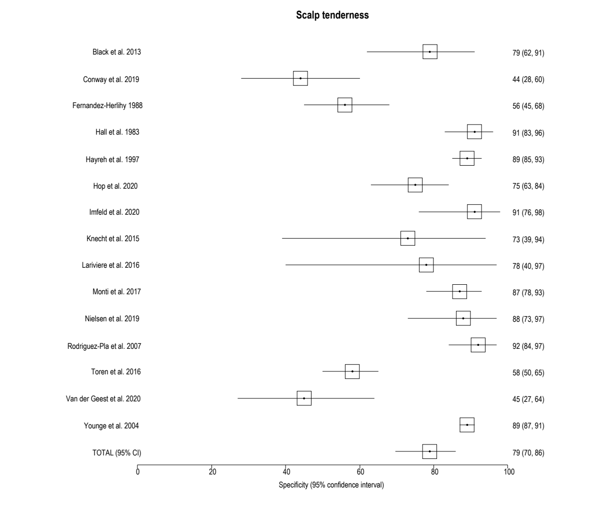

TA loss of pulse (Sensitivity)

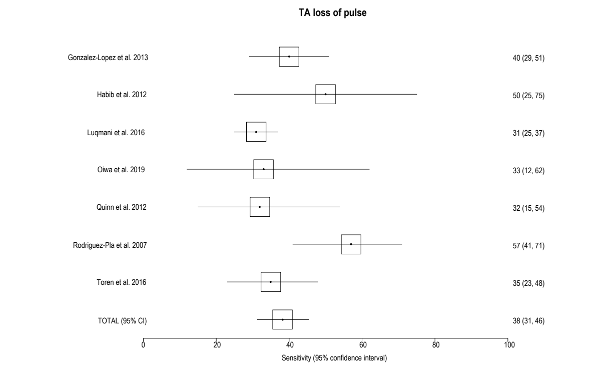

TA loss of pulse (Specificity)

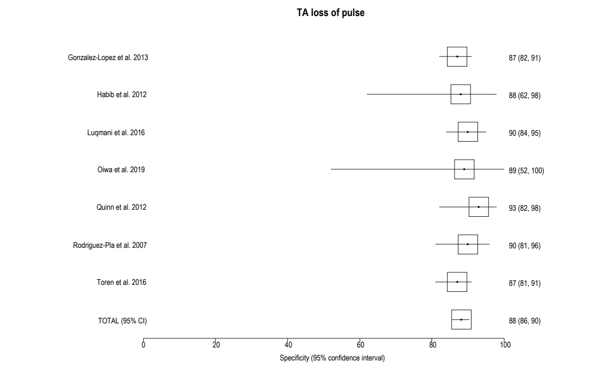

TA tenderness (Sensitivity)

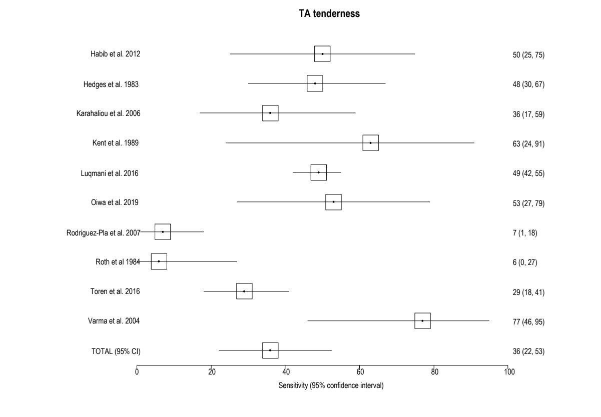

TA tenderness (Specificity)

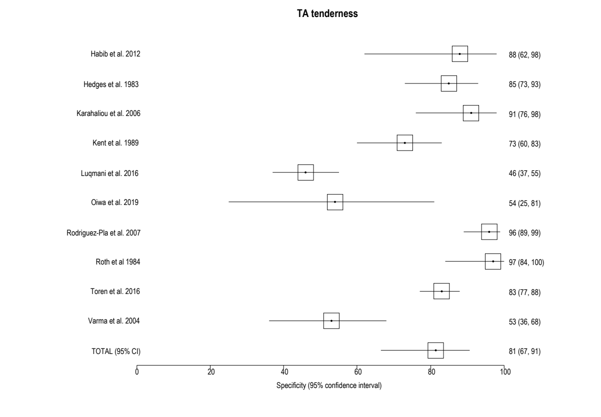

TA thickening (Sensitivity)

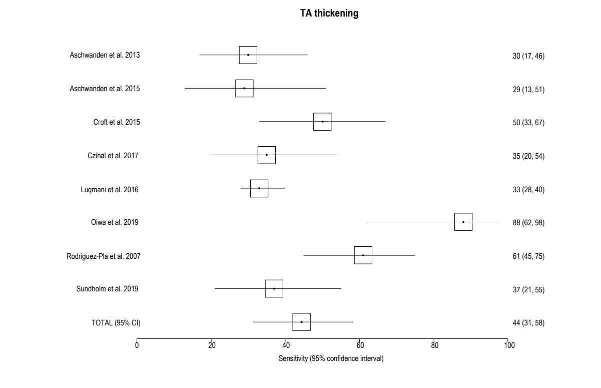

TA thickening (Specificity)

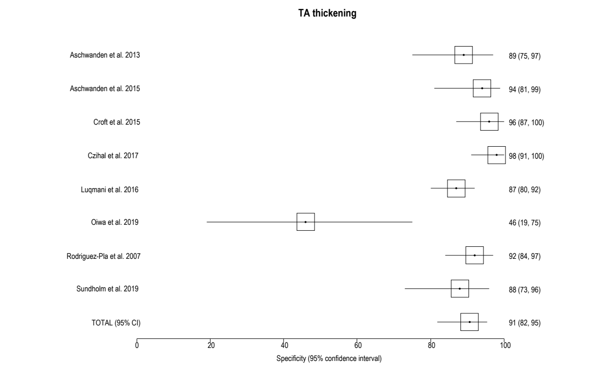

©2020 American Medical Association. All rights reserved.

Temporal headache (Sensitivity)	Temporal headache (Specificity)

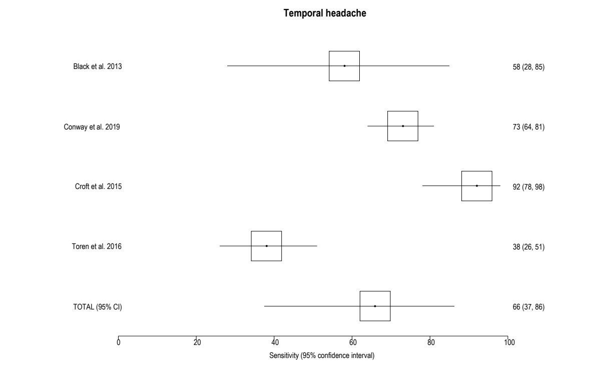

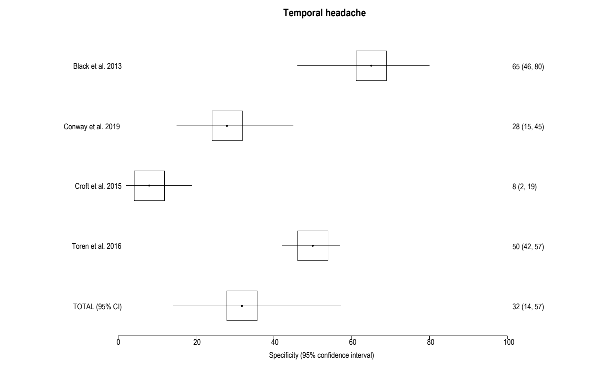

Temporal tenderness (Sensitivity)	Temporal tenderness (Specificity)

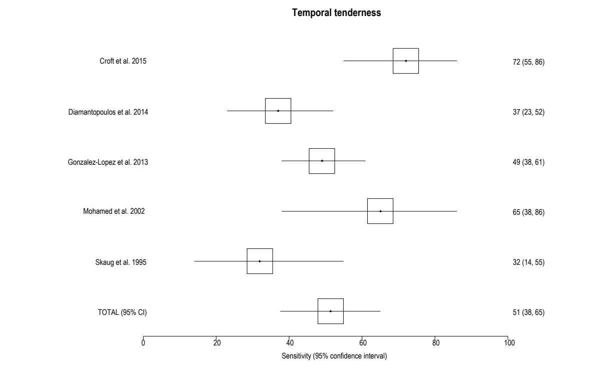

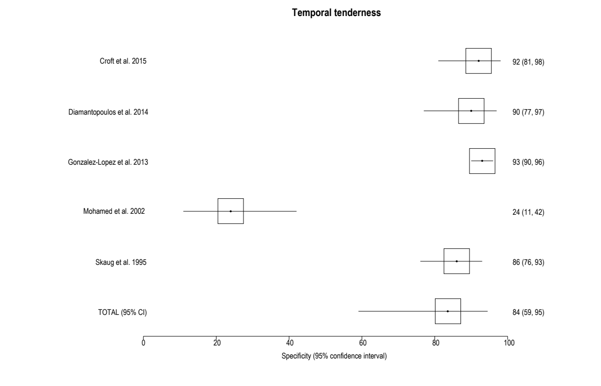

Transient loss of vision (Sensitivity)	Transient loss of vision (Specificity)

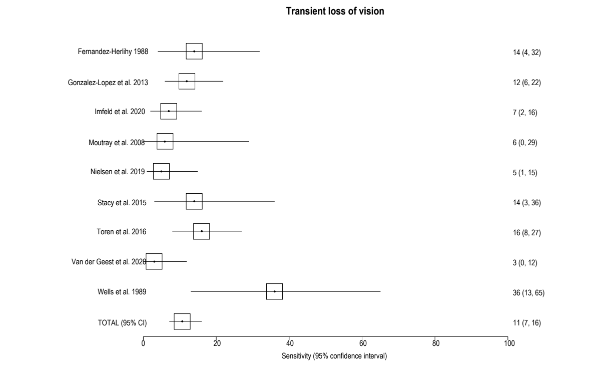

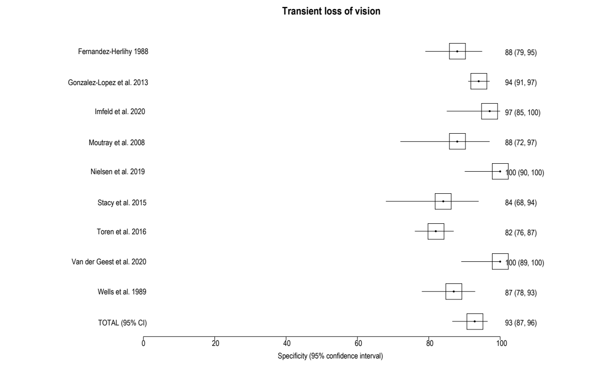

©2020 American Medical Association. All rights reserved.

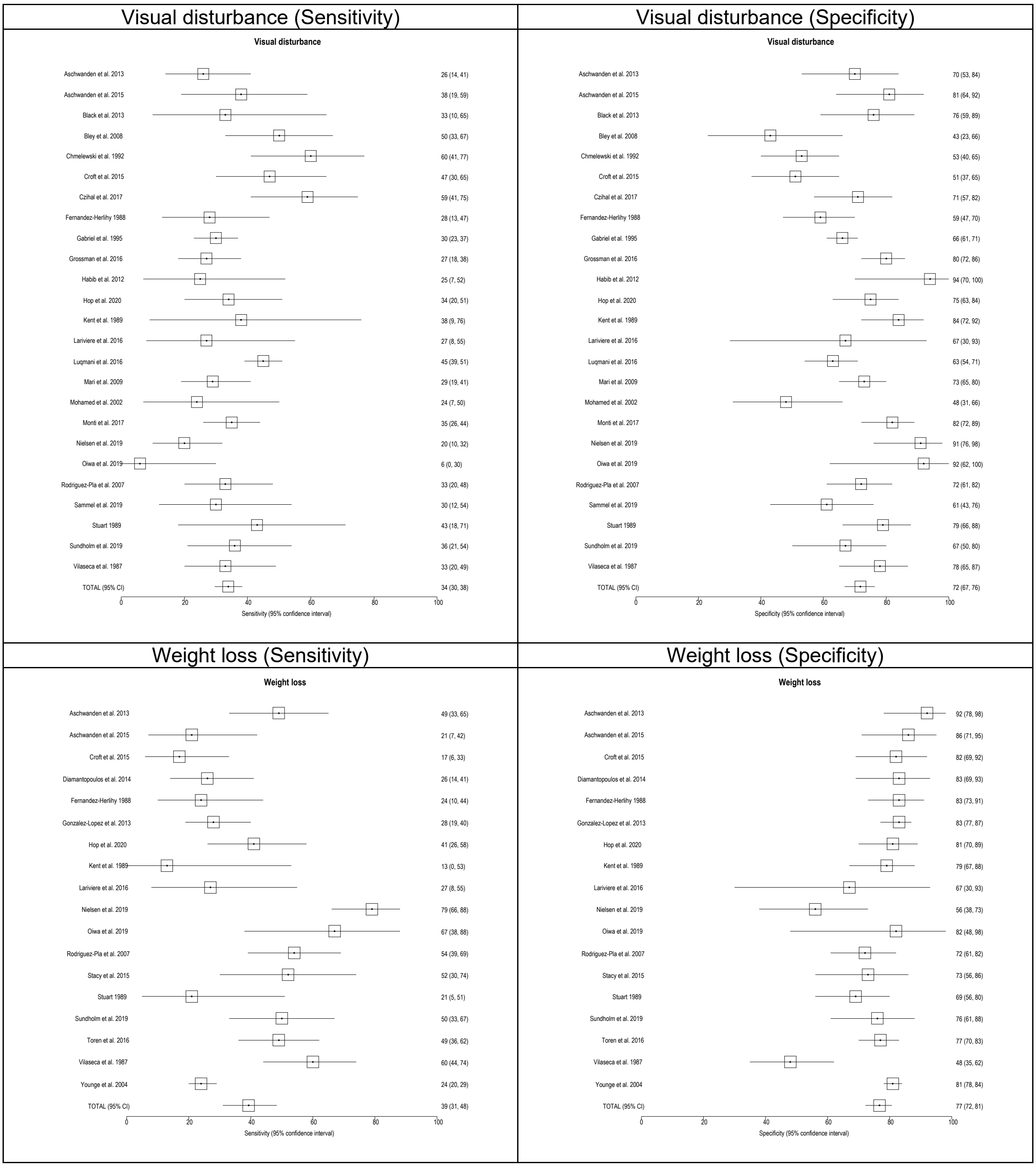

Forest plots are shown in alphabetical order for all demographic features, symptoms, physical findings and laboratory findings reported in Table 2-3. [a] studies reporting an ESR ≥ 50 mm/h or platelet count ≥ 400 $*10^3/\mu L$ were also included.

©2020 American Medical Association. All rights reserved.

eFigure 5. HSROC Curves

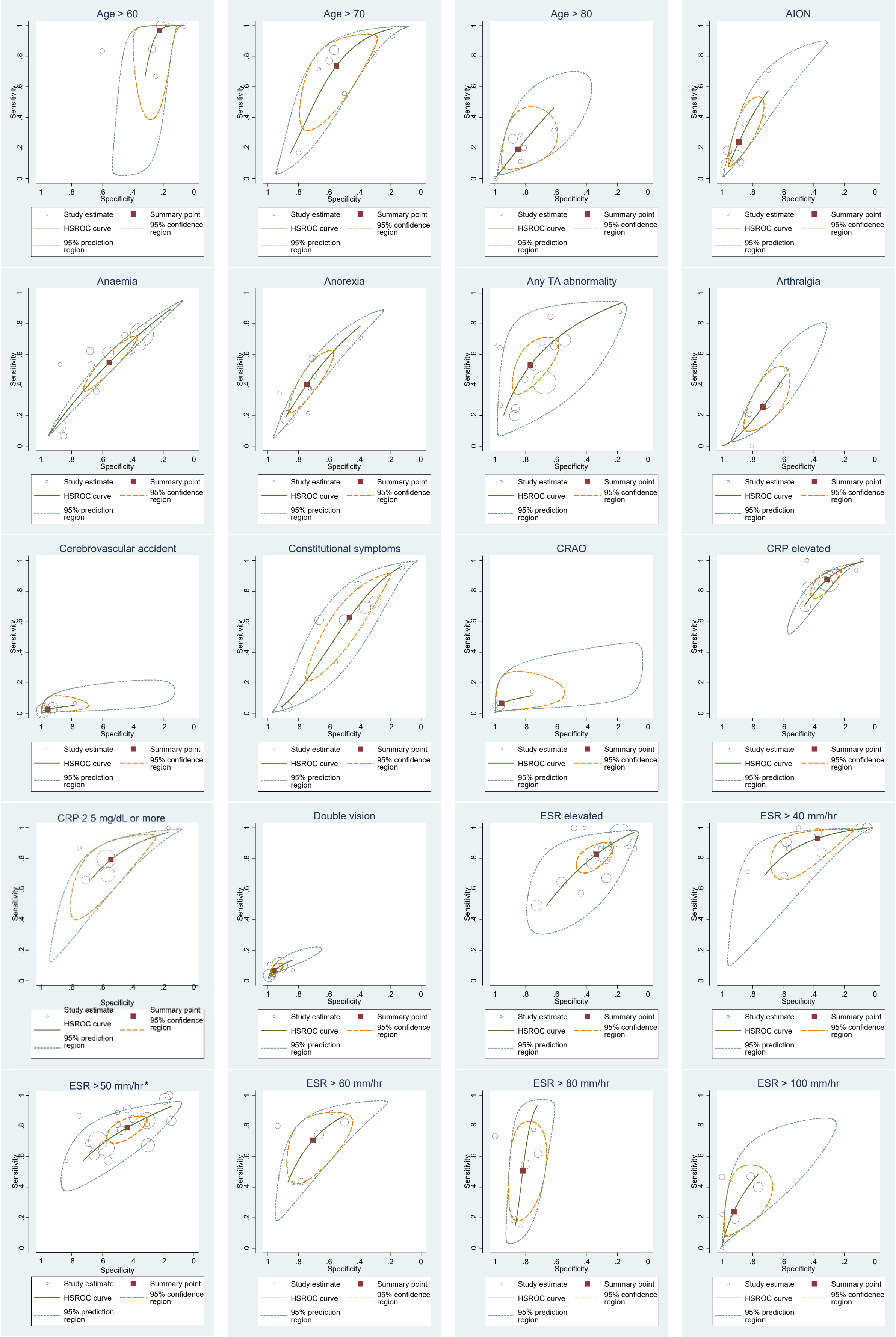

©2020 American Medical Association. All rights reserved.

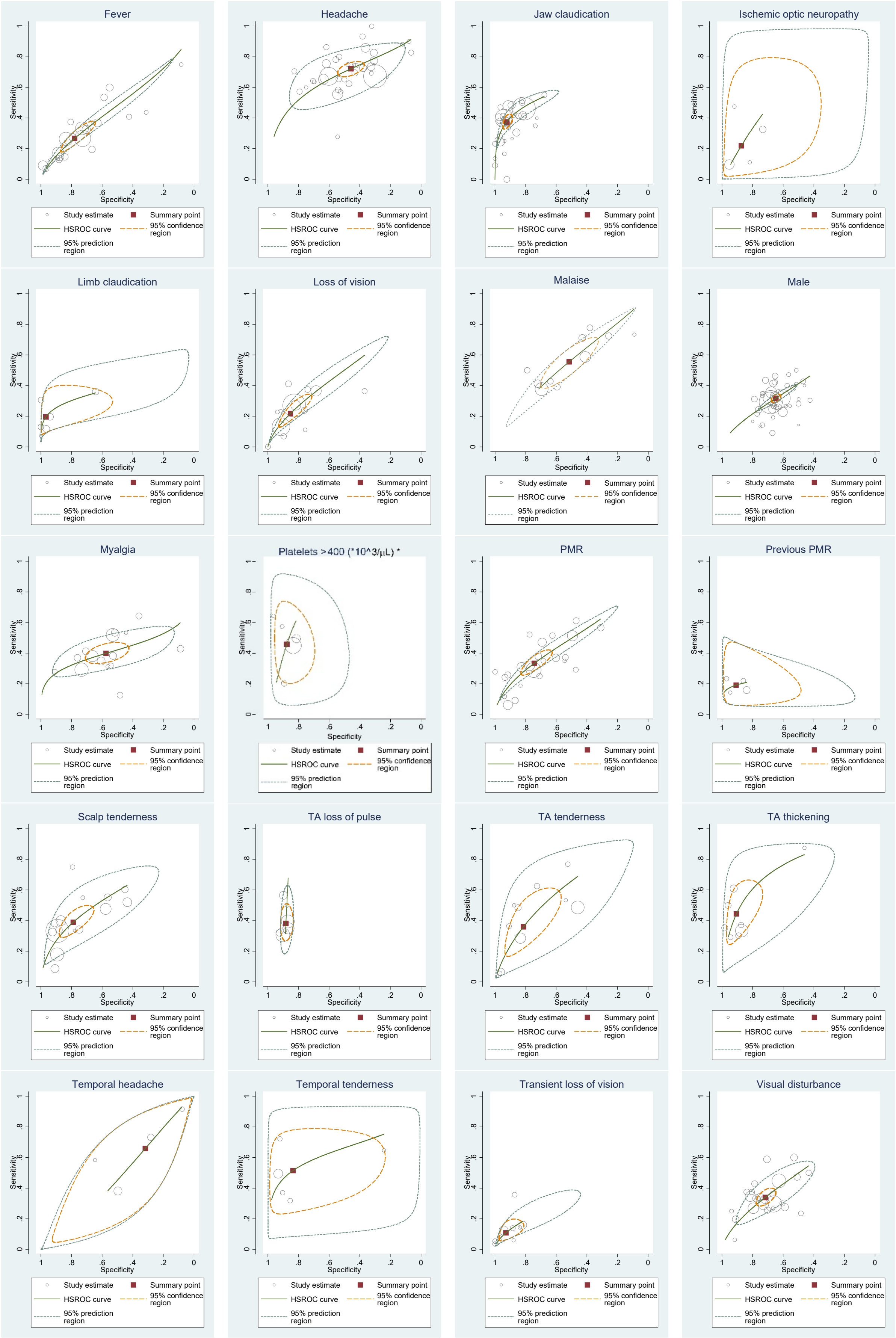

©2020 American Medical Association. All rights reserved.

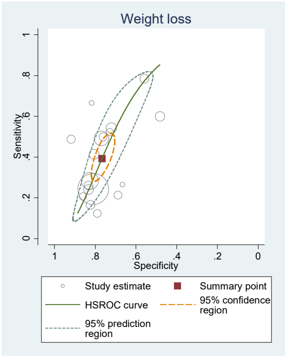

HSROC curves are shown in alphabetical order for all demographic features, symptoms, physical findings and laboratory findings reported in Table 2-3. * studies reporting an ESR ≥ 50 mm/h or platelet count ≥ 400 *10^3/μL were also included.

©2020 American Medical Association. All rights reserved.

eFigure 6. Funnel Plots

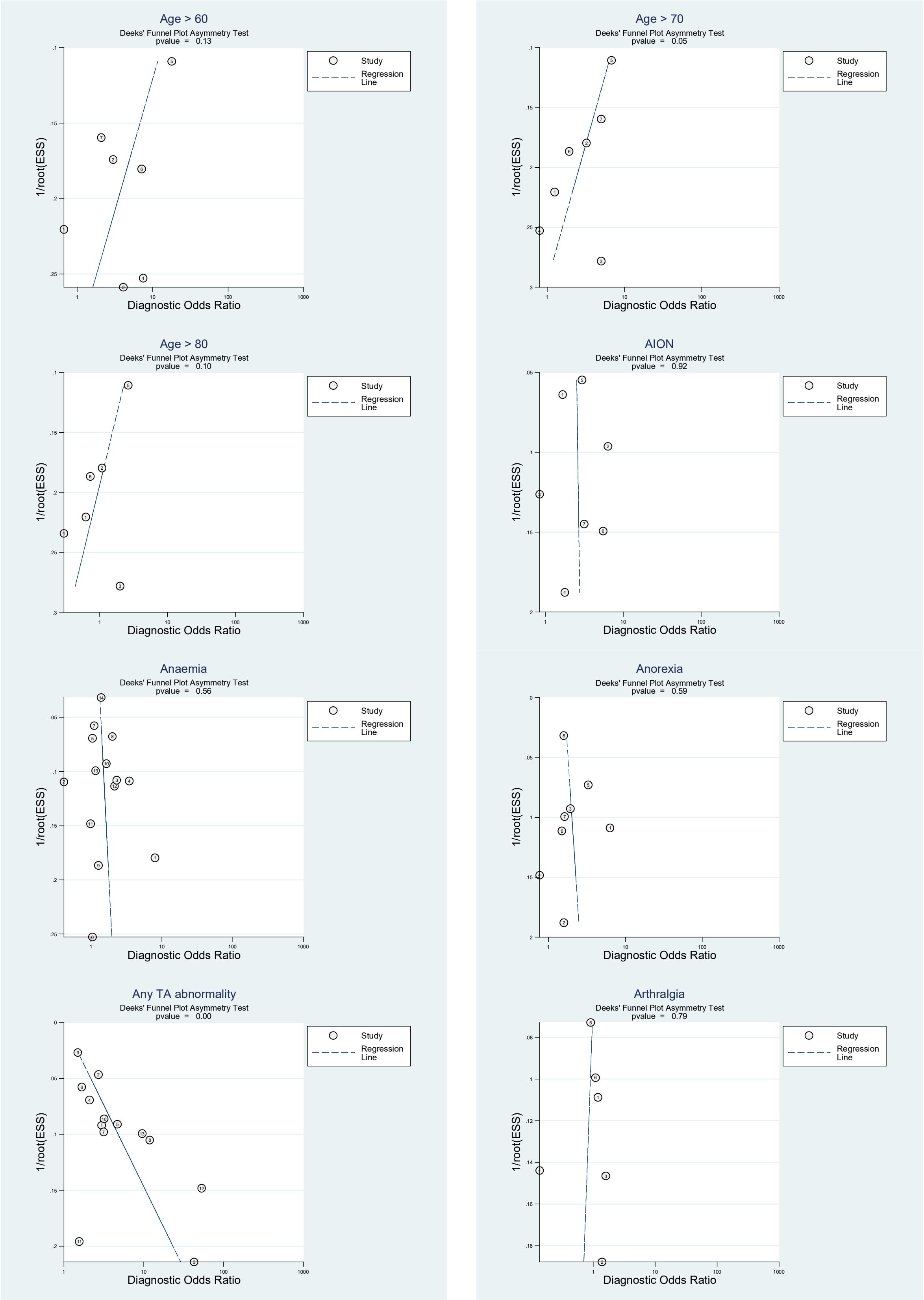

©2020 American Medical Association. All rights reserved.

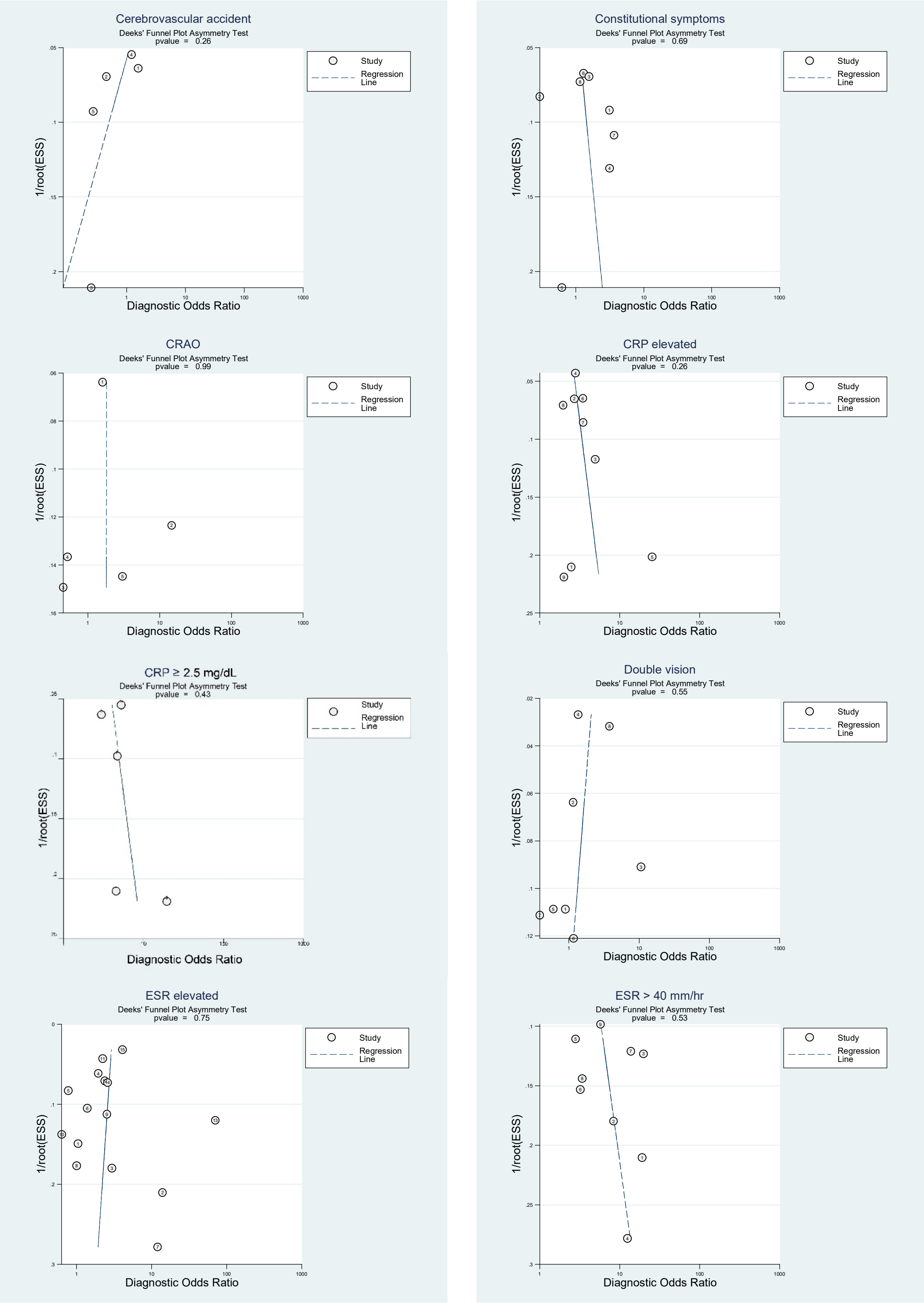

©2020 American Medical Association. All rights reserved.

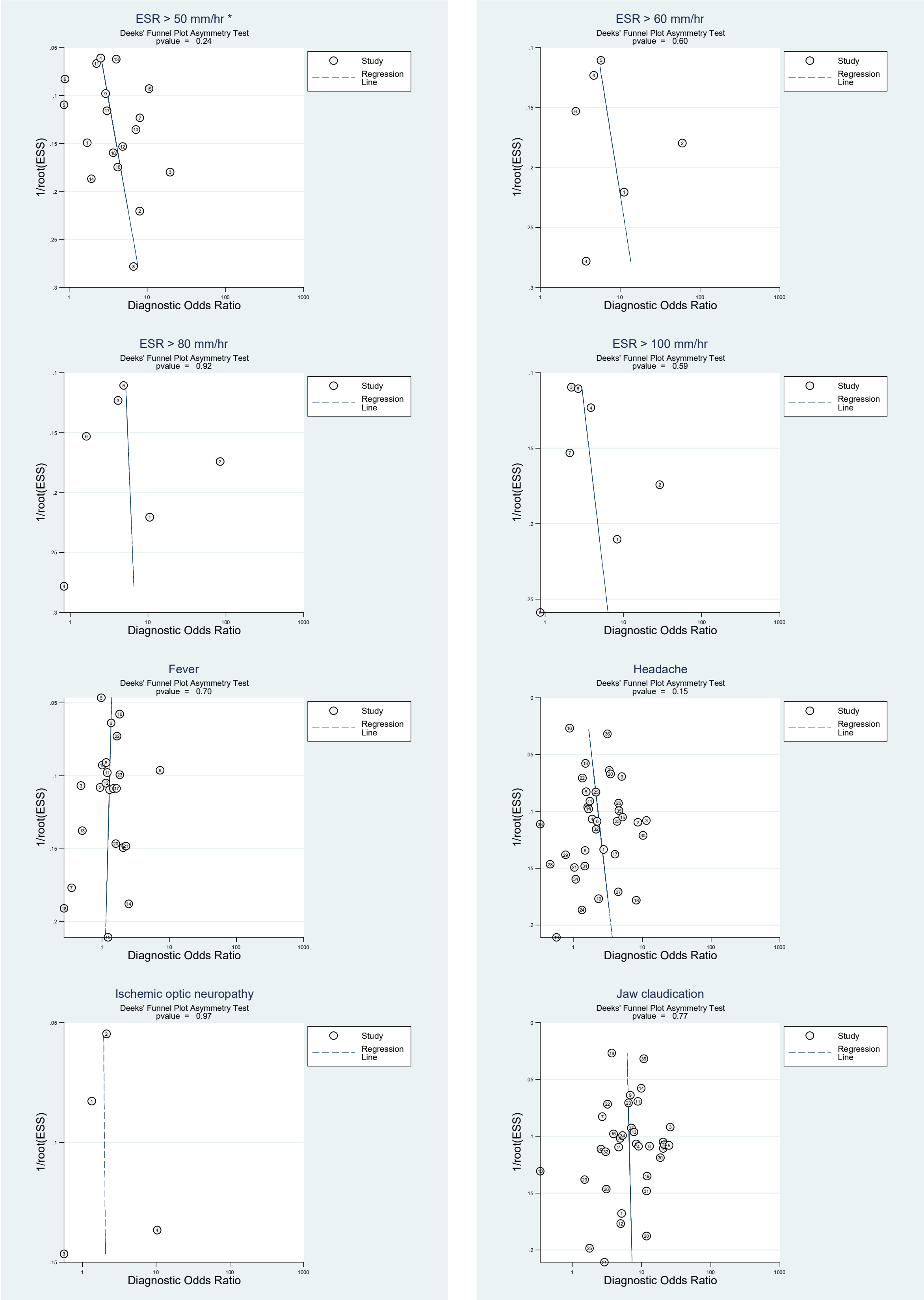

©2020 American Medical Association. All rights reserved.

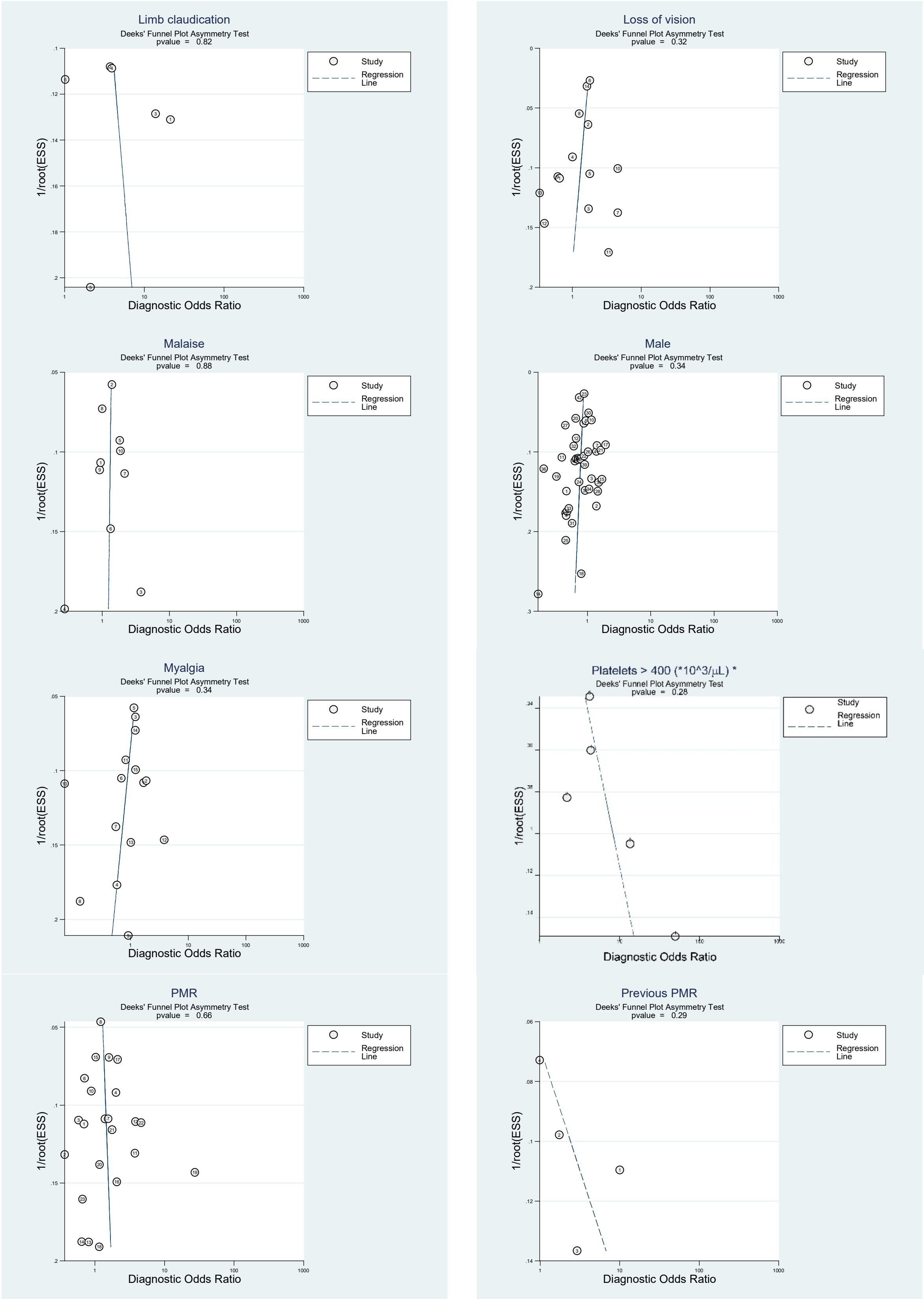

©2020 American Medical Association. All rights reserved.

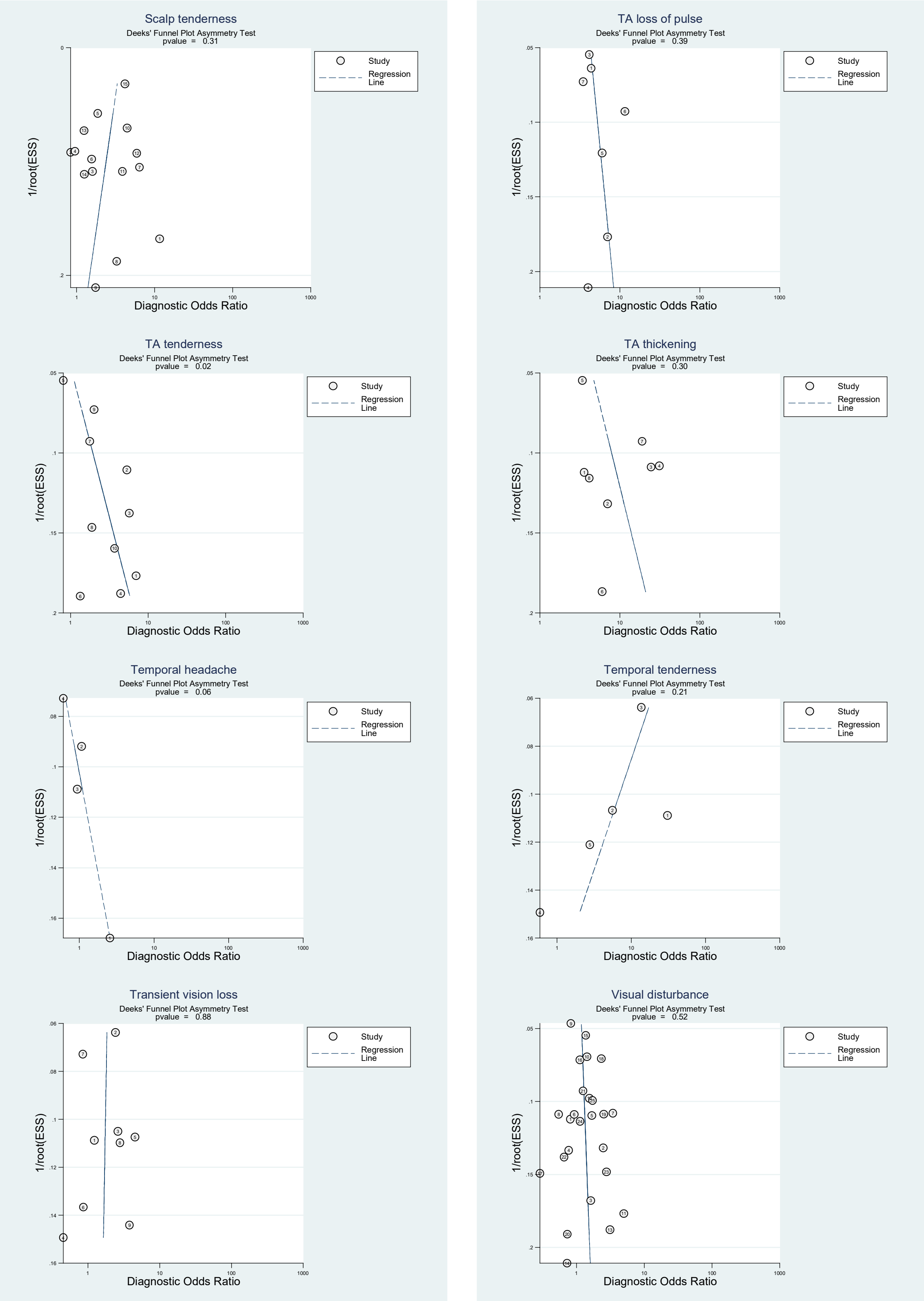

©2020 American Medical Association. All rights reserved.

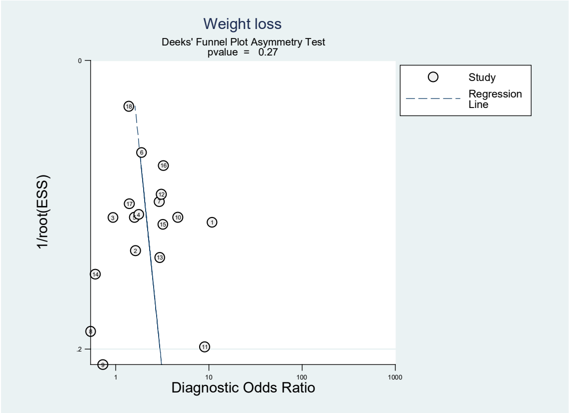

Effective sample size (ESS) funnel plots and the associated regression test of asymmetry, as reported by Deeks et al. [71], are shown. Plots are shown in alphabetical order for all demographic features, symptoms, physical findings and laboratory findings reported in Table 2-3. A *p* value < 0.10 was considered evidence of asymmetry and potential publication bias.

©2020 American Medical Association. All rights reserved.

eReferences

1. Aschwanden M, Daikeler T, Kesten F, et al. Temporal artery compression sign--a novel ultrasound finding for the diagnosis of giant cell arteritis. *Ultraschall Med*. 2013;34(1):47-50.
2. Aschwanden M, Imfeld S, Staub D, et al. The ultrasound compression sign to diagnose temporal giant cell arteritis shows an excellent interobserver agreement. *Clin Exp Rheumatol*. 2015;33(2 Suppl 89):S-113-5.
3. Bilyk JR, Murchison AP, Leiby BT, et al. The utility of color duplex ultrasonography in the diagnosis of giant cell arteritis: A prospective, masked study. (an american ophthalmological society thesis). *Trans Am Ophthalmol Soc*. 2018;115:T9.
4. Black R, Roach D, Rischmueller M, Lester SL, Hill CL. The use of temporal artery ultrasound in the diagnosis of giant cell arteritis in routine practice. *Int J Rheum Dis*. 2013;16(3):352-357.
5. Bley TA, Weiben O, Uhl M, et al. Assessment of the cranial involvement pattern of giant cell arteritis with 3T magnetic resonance imaging. *Arthritis Rheum*. 2005;52(8):2470-2477.
6. Bley TA, Reinhard M, Hauenstein C, et al. Comparison of duplex sonography and high-resolution magnetic resonance imaging in the diagnosis of giant cell (temporal) arteritis. *Arthritis Rheum*. 2008;58(8):2574-2578.
7. Brittain GP, McIlwaine GG, Bell JA, Gibson JM. Plasma viscosity or erythrocyte sedimentation rate in the diagnosis of giant cell arteritis? *Br J Ophthalmol*. 1991;75(11):656-659.
8. Chan FLY, Lester S, Whittle SL, Hill CL. The utility of ESR, CRP and platelets in the diagnosis of GCA. *BMC Rheumatol*. 2019;3:14-019-0061-z. eCollection 2019.
9. Chmelewski WL, McKnight KM, Agudelo CA, Wise CM. Presenting features and outcomes in patients undergoing temporal artery biopsy. A review of 98 patients. *Arch Intern Med*. 1992;152(8):1690-1695.
10. Conway R, O'Neill L, McCarthy GM, et al. Performance characteristics and predictors of temporal artery ultrasound for the diagnosis of giant cell arteritis in routine clinical practice in a prospective cohort. *Clin Exp Rheumatol*. 2019;37 Suppl 117(2):72-78.
11. Croft AP, Thompson N, Duddy MJ, et al. Cranial ultrasound for the diagnosis of giant cell arteritis. A retrospective cohort study. *J R Coll Physicians Edinb*. 2015;45(4):268-272.
12. Czihal M, Schrottle A, Baustel K, et al. B-mode sonography wall thickness assessment of the temporal and axillary arteries for the diagnosis of giant cell arteritis: A cohort study. *Clin Exp Rheumatol*. 2017;35 Suppl 103(1):128-133.
13. De Lott LB, Burke JF, Michigan Neuro-Ophthalmology Research Consortium. Use of laboratory markers in deciding whether to perform temporal artery biopsy. *JAMA Ophthalmol*. 2015;133(5):605-606.
14. Diamantopoulos AP, Haugeberg G, Hetland H, Soldal DM, Bie R, Myklebust G. Diagnostic value of color doppler ultrasonography of temporal arteries and large vessels in giant cell arteritis: A consecutive case series. *Arthritis Care Res (Hoboken)*. 2014;66(1):113-119.
15. El-Dairi MA, Chang L, Proia AD, Cummings TJ, Stinnett SS, Bhatti MT. Diagnostic algorithm for patients with suspected giant cell arteritis. *J Neuroophthalmol*. 2015;35(3):246-253.
16. Eshaghian J, Goeken JA. C-reactive protein in giant cell (cranial, temporal) arteritis. *Ophthalmology*. 1980;87(11):1160-1166.
17. Fernandez-Herlihy L. Temporal arteritis: Clinical aids to diagnosis. *J Rheumatol*. 1988;15(12):1797-1801.
18. Foroozan R, Danesh-Meyer H, Savino PJ, Gamble G, Mekari-Sabbagh ON, Sergott RC. Thrombocytosis in patients with biopsy-proven giant cell arteritis. *Ophthalmology*. 2002;109(7):1267-1271.
19. Gabriel SE, O'Fallon WM, Achkar AA, Lie JT, Hunder GG. The use of clinical characteristics to predict the results of temporal artery biopsy among patients with suspected giant cell arteritis. *J Rheumatol*. 1995;22(1):93-96.

©2020 American Medical Association. All rights reserved.

20. Ghinoi A, Zuccoli G, Nicolini A, et al. 1T magnetic resonance imaging in the diagnosis of giant cell arteritis: Comparison with ultrasonography and physical examination of temporal arteries. *Clin Exp Rheumatol*. 2008;26(3 Suppl 49):S76-80.
21. Gonzalez-Lopez JJ, Gonzalez-Moraleja J, Burdaspal-Moratilla A, Rebolleda G, Nunez-Gomez-Alvarez MT, Munoz-Negrete FJ. Factors associated to temporal artery biopsy result in suspects of giant cell arteritis: A retrospective, multicenter, case-control study. *Acta Ophthalmol*. 2013;91(8):763-768.
22. Gospe SM,3rd, Amrhein TJ, Malinzak MD, Bhatti MT, Mettu P, El-Dairi MA. Magnetic resonance imaging abnormalities of the optic nerve sheath and intracranial internal carotid artery in giant cell arteritis. *J Neuroophthalmol*. 2019;doi: 10.1097/WNO. 0000000000000860 [Epub ahead of print].
23. Grosser SJ, Reddy RK, Tomsak RL, Katzin WE. Temporal arteritis in african americans. *Neuro-Ophthalmology*. 1999;21(1):25-31.
24. Grossman C, Barshack I, Koren-Morag N, Ben-Zvi I, Bornstein G. Baseline clinical predictors of an ultimate giant cell arteritis diagnosis in patients referred to temporal artery biopsy. *Clin Rheumatol*. 2016;35(7):1817-1822.
25. Habib HM, Essa AA, Hassan AA. Color duplex ultrasonography of temporal arteries: Role in diagnosis and follow-up of suspected cases of temporal arteritis. *Clin Rheumatol*. 2012;31(2):231-237.
26. Hall JK, Volpe NJ, Galetta SL, Liu GT, Syed NA, Balcer LJ. The role of unilateral temporal artery biopsy. *Ophthalmology*. 2003;110(3):543-8; discussion 548.
27. Hall S, Persellin S, Lie JT, O'Brien PC, Kurland LT, Hunder GG. The therapeutic impact of temporal artery biopsy. *Lancet*. 1983;2(8361):1217-1220.
28. Hautzel H, Sander O, Heinzel A, Schneider M, Muller HW. Assessment of large-vessel involvement in giant cell arteritis with 18F-FDG PET: Introducing an ROC-analysis-based cutoff ratio. *J Nucl Med*. 2008;49(7):1107-1113.
29. Hay B, Mariano-Goulart D, Bourdon A, et al. Diagnostic performance of (18)F-FDG PET-CT for large vessel involvement assessment in patients with suspected giant cell arteritis and negative temporal artery biopsy. *Ann Nucl Med*. 2019;33(7):512-520.
30. Hayreh SS, Podhajsky PA, Raman R, Zimmerman B. Giant cell arteritis: Validity and reliability of various diagnostic criteria. *Am J Ophthalmol*. 1997;123(3):285-296.
31. Hedges TR,3rd, Gieger GL, Albert DM. The clinical value of negative temporal artery biopsy specimens. *Arch Ophthalmol*. 1983;101(8):1251-1254.
32. Hop H, Mulder DJ, Sandovici M, et al. Diagnostic value of axillary artery ultrasound in patients with suspected giant cell arteritis. *Rheumatology (Oxford)*. 2020;keaa102:https://doi-org.proxy-ub.rug.nl/10.1093/rheumatology/keaa102.
33. Imfeld S, Aschwanden M, Rottenburger C, et al. 18F]FDG positron emission tomography and ultrasound in the diagnosis of giant cell arteritis: Congruent or complementary imaging methods? *Rheumatology (Oxford)*. 2020;59(4):772-778.
34. Ing E, Pagnoux C, Tyndel F, et al. Lower ocular pulse amplitude with dynamic contour tonometry is associated with biopsy-proven giant cell arteritis. *Can J Ophthalmol*. 2018;53(3):215-221.
35. Ing EB, Miller NR, Nguyen A, et al. Neural network and logistic regression diagnostic prediction models for giant cell arteritis: Development and validation. *Clin Ophthalmol*. 2019;13:421-430.
36. Karahaliou M, Vaiopoulos G, Papaspyrou S, Kanakis MA, Revenas K, Sfikakis PP. Colour duplex sonography of temporal arteries before decision for biopsy: A prospective study in 55 patients with suspected giant cell arteritis. *Arthritis Res Ther*. 2006;8(4):R116.
37. Kent RB,3rd, Thomas L. Temporal artery biopsy. *Am Surg*. 1990;56(1):16-21.
38. Kermani TA, Schmidt J, Crowson CS, et al. Utility of erythrocyte sedimentation rate and C-reactive protein for the diagnosis of giant cell arteritis. *Semin Arthritis Rheum*. 2012;41(6):866-871.

©2020 American Medical Association. All rights reserved.

39. Knecht PB, Bachmann LM, Thiel MA, Landau K, Kaufmann C. Ocular pulse amplitude as a diagnostic adjunct in giant cell arteritis. *Eye (Lond)*. 2015;29(7):860-5; quiz 866.
40. Lariviere D, Benali K, Coustet B, et al. Positron emission tomography and computed tomography angiography for the diagnosis of giant cell arteritis: A real-life prospective study. *Medicine (Baltimore)*. 2016;95(30):e4146.
41. Lugo JZ, Deitch JS, Yu A, et al. Demographic and laboratory data may predict positive temporal artery biopsy. *J Surg Res*. 2011;170(2):332-335.
42. Luqmani R, Lee E, Singh S, et al. The role of ultrasound compared to biopsy of temporal arteries in the diagnosis and treatment of giant cell arteritis (TABUL): A diagnostic accuracy and cost-effectiveness study. *Health Technol Assess*. 2016;20(90):1-238.
43. Mari B, Monteagudo M, Bustamante E, et al. Analysis of temporal artery biopsies in an 18-year period at a community hospital. *Eur J Intern Med*. 2009;20(5):533-536.
44. Mohamed MS, Bates T. Predictive clinical and laboratory factors in the diagnosis of temporal arteritis. *Ann R Coll Surg Engl*. 2002;84(1):7-9.
45. Monti S, Floris A, Ponte CB, et al. The proposed role of ultrasound in the management of giant cell arteritis in routine clinical practice. *Rheumatology (Oxford)*. 2018;57(1):112-119.
46. Moutray TN, Williams MA, Best JL. Suspected giant cell arteritis: A study of referrals for temporal artery biopsy. *Can J Ophthalmol*. 2008;43(4):445-448.
47. Mukhtyar C, Myers H, Scott DGI, Misra A, Jones C. Validating a diagnostic GCA ultrasonography service against temporal artery biopsy and long-term clinical outcomes. *Clin Rheumatol*. 2020;39(4):1325-1329.
48. Nielsen BD, Hansen IT, Keller KK, Therkildsen P, Gormsen LC, Hauge EM. Diagnostic accuracy of ultrasound for detecting large-vessel giant cell arteritis using FDG PET/CT as the reference. *Rheumatology (Oxford)*. 2019;kez568:https://doi-org.proxy-ub.rug.nl/10.1093/rheumatology/kez568.
49. Oh LJ, Wong E, Andrici J, McCluskey P, Smith JEH, Gill AJ. Full blood count as an ancillary test to support the diagnosis of giant cell arteritis. *Intern Med J*. 2018;48(4):408-413.
50. Oiwa H, Ichimura K, Hosokawa Y, et al. Diagnostic performance of a temporal artery biopsy for the diagnosis of giant cell arteritis in japan-A single-center retrospective cohort study. *Intern Med*. 2019;58(17):2451-2458.
51. Quinn EM, Kearney DE, Kelly J, Keohane C, Redmond HP. Temporal artery biopsy is not required in all cases of suspected giant cell arteritis. *Ann Vasc Surg*. 2012;26(5):649-654.
52. Rodriguez-Pla A, Rossello-Urgell J, Bosch-Gil JA, Huguet-Redecilla P, Vilardell-Tarres M. Proposal to decrease the number of negative temporal artery biopsies. *Scand J Rheumatol*. 2007;36(2):111-118.
53. Roncato C, Allix-Beguec C, Brottier-Mancini E, Gombert B, Denis G. Diagnostic performance of colour duplex ultrasonography along with temporal artery biopsy in suspicion of giant cell arteritis. *Clin Exp Rheumatol*. 2017;35 Suppl 103(1):119-122.
54. Roth AM, Milsow L, Keltner JL. The ultimate diagnoses of patients undergoing temporal artery biopsies. *Arch Ophthalmol*. 1984;102(6):901-903.
55. Sammel AM, Smith S, Nguyen K, et al. Assessment for varicella zoster virus in patients newly suspected of having giant cell arteritis. *Rheumatology (Oxford)*. 2019;kez556:https://doi-org.proxy-ub.rug.nl/10.1093/rheumatology/kez556.
56. Skaug TR, Midelfart A, Jacobsen G. Clinical usefulness of biopsy in giant cell arteritis. *Acta Ophthalmol Scand*. 1995;73(6):567-570.
57. Sommer F, Sporl E, Herber R, Pillunat LE, Terai N. Predictive value of positive temporal artery biopsies in patients with clinically suspected giant cell arteritis considering temporal artery ultrasound findings. *Graefes Arch Clin Exp Ophthalmol*. 2019;257(10):2279-2284.
58. Stacy RC, Gilbert AL, Rizzo JF,3rd. Correlation of clinical profile and specific histopathological features of temporal artery biopsies. *J Neuroophthalmol*. 2015;35(2):127-133.

©2020 American Medical Association. All rights reserved.

59. Stuart RA. Temporary artery biopsy in suspected temporal arteritis: A five year survey. *N Z Med J*. 1989;102(874):431-433.
60. Suelves AM, Espana-Gregori E, Avino J, Rohrweck S, Diaz-Llopis M. Analysis of factors that determine the diagnostic yield of temporal artery biopsy. *Arch Soc Esp Oftalmol*. 2013;88(4):127-129.
61. Sundholm JKM, Pettersson T, Paetau A, Alback A, Sarkola T. Diagnostic performance and utility of very high-resolution ultrasonography in diagnosing giant cell arteritis of the temporal artery. *Rheumatol Adv Pract*. 2019;3(2):rkz018.
62. Toren A, Weis E, Patel V, Monteith B, Gilberg S, Jordan D. Clinical predictors of positive temporal artery biopsy. *Can J Ophthalmol*. 2016;51(6):476-481.
63. van der Geest KSM, Borg F, Kayani A, et al. Novel ultrasonographic halo score for giant cell arteritis: Assessment of diagnostic accuracy and association with ocular ischaemia. *Ann Rheum Dis*. 2020;79(3):393-399.
64. Varma D, O'Neill D. Quantification of the role of temporal artery biopsy in diagnosing clinically suspected giant cell arteritis. *Eye (Lond)*. 2004;18(4):384-388.
65. Vilaseca J, Gonzalez A, Cid MC, Lopez-Vivancos J, Ortega A. Clinical usefulness of temporal artery biopsy. *Ann Rheum Dis*. 1987;46(4):282-285.
66. Walvick MD, Walvick MP. Giant cell arteritis: Laboratory predictors of a positive temporal artery biopsy. *Ophthalmology*. 2011;118(6):1201-1204.
67. Wells KK, Folberg R, Goeken JA, Kemp JD. Temporal artery biopsies. correlation of light microscopy and immunofluorescence microscopy. *Ophthalmology*. 1989;96(7):1058-1064.
68. Younge BR, Cook BE,Jr, Bartley GB, Hodge DO, Hunder GG. Initiation of glucocorticoid therapy: Before or after temporal artery biopsy? *Mayo Clin Proc*. 2004;79(4):483-491.
69. Takwoingi Y, Guo B, Riley RD, Deeks JJ. Performance of methods for meta-analysis of diagnostic test accuracy with few studies or sparse data. *Stat Methods Med Res*. 2017;26(4):1896-1911.
70. Higgins JP, Thompson SG, Deeks JJ, Altman DG. Measuring inconsistency in meta-analyses. *BMJ*. 2003;327(7414):557-560.
71. Deeks JJ, Macaskill P, Irwig L. The performance of tests of publication bias and other sample size effects in systematic reviews of diagnostic test accuracy was assessed. *J Clin Epidemiol*. 2005;58(9):882-893.

©2020 American Medical Association. All rights reserved.
